# Design, Synthesis, and Biological Evaluation of Sulfonamide Methoxypyridine Derivatives as Novel PI3K/mTOR Dual Inhibitors

**DOI:** 10.3390/ph16030461

**Published:** 2023-03-20

**Authors:** Haotian Gao, Zaolin Li, Kai Wang, Yuhan Zhang, Tong Wang, Fang Wang, Youjun Xu

**Affiliations:** 1 Key Laboratory of Structure-Based Drug Design and Discovery (Ministry of Education), School of Pharmaceutical Engineering, Shenyang Pharmaceutical University, Shenyang 110016, China; 2 Faculty of Functional Food and Wine, Shenyang Pharmaceutical University, Shenyang 110016, China

**Keywords:** PI3K/mTOR, inhibitor, anti-proliferation, apoptosis, cancer

## Abstract

Phosphatidylinositol 3-kinase (PI3K) plays an important role in cell proliferation, survival, migration, and metabolism, and has become an effective target for cancer treatment. Meanwhile, inhibiting both PI3K and mammalian rapamycin receptor (mTOR) can simultaneously improve the efficiency of anti-tumor therapy. Herein, a series of 36 sulfonamide methoxypyridine derivatives with three different aromatic skeletons were synthesized as novel potent PI3K/mTOR dual inhibitors based on a scaffold hopping strategy. Enzyme inhibition assay and cell anti-proliferation assay were employed to assess all derivatives. Then, the effects of the most potent inhibitor on cell cycle and apoptosis were performed. Furthermore, the phosphorylation level of AKT, an important downstream effector of PI3K, was evaluated by Western blot assay. Finally, molecular docking was used to confirm the binding mode with PI3Kα and mTOR. Among them, **22c** with the quinoline core showed strong PI3Kα kinase inhibitory activity (IC_50_ = 0.22 nM) and mTOR kinase inhibitory activity (IC_50_ = 23 nM). **22c** also showed a strong proliferation inhibitory activity, both in MCF-7 cells (IC_50_ = 130 nM) and HCT-116 cells (IC_50_ = 20 nM). **22c** could effectively cause cell cycle arrest in G0/G1 phase and induce apoptosis of HCT-116 cells. Western blot assay showed that **22c** could decrease the phosphorylation of AKT at a low concentration. The results of the modeling docking study also confirmed the binding mode of **22c** with PI3Kα and mTOR. Hence, **22c** is a promising PI3K/mTOR dual inhibitor, which is worthy of further research in the area.

## 1. Introduction

Phosphatidylinositol 3-kinase (PI3K), a lipid kinase activated by several transmembrane receptors including receptor tyrosine kinases (RTKs) and G protein coupled receptors, generates phosphatidylinositol-3,4,5-triphosphate (PIP3) from phosphatidylinositol 4,5-diphosphate (PIP2), and leads to complex downstream signaling protein activities and secondary messenger functions inside the cell [1,2]. As a central intersection signaling kinase, PI3K plays an essential role in cell proliferation, survival, migration, and metabolism [3]. PI3Ks have three classes (Class IA/B, II, III), with Class IA PI3Ks being the most prevalent type that plays a role in lymphocyte signaling [4]. In mammals, class I PI3Ks contains four homologous enzymes: PI3K α, β, γ, and δ, all of which are heterodimers consisting of a regulatory subunit in complex with a 110 kDa catalytic subunit called p110α (PI3Kα), β (PI3Kβ), γ (PI3Kγ), or δ (PI3Kδ) [5]. Although P110α and β are distributed widely in mammals, P110γ and δ are enormously enriched in all kinds of leukocytes. When PI3K is abnormally expressed in cancer, increased AKT (protein kinase B, PKB) phosphorylation contributes to cancer progression [6]. Mammalian target of rapamycin (mTOR) is an atypia of serine/threonine kinase that regulates signaling and metabolic pathways through two distinct protein complexes, mTOR complex 1 (mTORC1) and mTOR complex 2 (mTORC2) [7]. As a result, maintaining strict control of cell growth and proliferation depends on the regulation of mTOR interactions, or mTOR activity. Cancer genome analyses have found that many cancers have mutations affecting the PI3K/Akt/mTOR signaling pathway [8]. The oncogenic PI3K/AKT pathway is negatively regulated by PTEN (phosphatase and tensin homolog deleted on chromosome ten), which dephosphorylates phosphoinositide-3,4,5-triphosphate (PIP3) [9]. Overall, the disruption of PI3K/AKT/mTOR signaling has been implicated in cancer, immunological disorders, diabetes, and cardiovascular disease, and led to the development of therapies targeted at PI3K pathways.

Even though PI3K is required for PI3K-mediated activation of AKT in a multitude of studies, there are reports suggesting that AKT can be activated independently by PI3K binding with phosphoinositide [10]. The inhibition of mTOR kinase lowers feedback inhibition of RTKs, allowing PI3K to activate and re-phosphorylate AKT T308 sufficiently to reactivate AKT activity [6]. Thus, one strategy to prevent feedback loop activation of AKT is to inhibit mTOR complex 1 and 2 at the same time [11]. In chronic lymphocytic leukemia patients, the inhibition of mTOR can enhance AKT signaling, which can be overcome by dual PI3K/mTOR inhibitors [12]. These studies indicate that the combination therapy strategy of simultaneously blocking AKT activation and inhibiting mTOR activity may improve the efficiency of anti-tumor therapy.

So far, more than 40 inhibitors of PI3K/AKT/mTOR pathway have been approved for clinical research, but only two mTOR inhibitors (Temsirolimus and Everolimus) and five PI3K inhibitors (Idelalisib, Copanlisib, Alpelisib, Duvelisb, and Umbralisib) have been approved for listing. The development and progress of PI3K inhibitors is still challenging due to poor drug tolerance, acquired drug resistance, and the hindrance of PI3K inhibitors’ compensatory mechanism.

**Figure 1:**
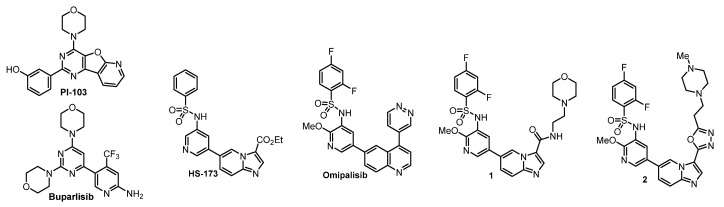
The chemical structures of some representative PI3K/mTOR dual inhibitors.

Inhibitors of PI3K are classified as the dual PI3K/mTOR, pan-PI3K, and isoform-specific [13]. There are several novel PI3K/mTOR inhibitors that have entered clinical trials, of which both pan-specific PI3K/mTOR and pan-PI3K inhibitors have the highest potential for broad therapies (as shown in Figure 1). For example, Omipalisib shows extremely strong inhibitory activity (PI3Kα Ki = 0.019 nM; PI3Kβ Ki = 0.13 nM; PI3Kγ Ki = 0.024 nM; PI3Kδ Ki =0.06 nM; mTORC1 Ki = 0.18 nM; mTORC2 Ki = 0.3 nM) [14], and has entered phase I clinical trials for lymphoma, solid tumors, and idiopathic pulmonary fibrosis. In brief, the use of PI3K/mTOR dual inhibitors can fully inhibit the abnormal activation of PI3K/AKT/mTOR signaling pathway, and block the compensatory activation of the AKT/mTOR pathway. However, most of them are also associated with the highest toxicity burden [1], as no PI3K/mTOR dual inhibitor has been approved yet by the FDA. Hence, the urgent problem is to balance the efficacy, toxicity, and side effects in the area herein.

Kim et al. [15] reported **HS-173**, a new PI3K inhibitor of imidazo[1,2-a]pyridine, which showed excellent enzyme inhibition at about 0.8 nM. Fan et al. [16] synthesized compound **1** with an amide moiety to significantly improve the metabolic stability while retaining the inhibitory activity. Yu et al. [17] further improved the design and reported compound **2** containing a hetero-linker in the structure with an increased activity of up to an IC_50_ value of 0.2 nM for PI3K, and it showed inhibition in HCT-116 cells at 0.01 μM.

In this paper, we designed and synthesized three series of compounds as potent PI3K/mTOR dual inhibitors containing fragments of benzo[4,5]thiopheno[3,2-*d*]pyrimidine, pyridine[2,3-*d*]pyrimidine, and quinoline. This was expected to obtain potent PI3K/mTOR dual inhibitors among them and summarize the structure–activity relationship in order to lay a foundation for further research in the field of treatment of malignant tumors.

## 2. Results and Discussion

### 2.1. Optimization Strategy

**Figure 2:**
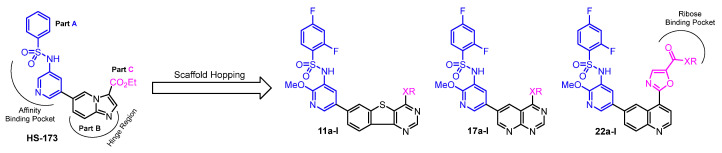
Design strategy of sulfonamide methoxypyridine derivatives as PI3K/mTOR dual inhibitors.

Based on the co-crystal structures of the lipid kinase PI3Kα and structures of some known PI3K/mTOR dual inhibitors, it could be found that the structures mainly consisted of three parts (as shown in Figure 2): part A for the affinity binding pocket, part B for the hinge binding pocket, and part C for the ribose binding pocket. Parts A and B were essential to the activity, and the optimization of part C could improve the metabolic stability and oral bioavailability. The available results suggested [14] that when the structure of part A was 2,4-difluoro-*N*-(2-methoxypyridin-3-yl) benzenesulfonamide, it had the strongest PI3K inhibitory activity. Therefore, the optimization was mainly in parts B and part C.

In previous studies [15,16,17], the structures of part B were diverse, but they all contained *N* heteroatoms, which interacted with the key amino acid residue Val851 in the hinge region. In this paper, in order to enhance the binding ability with the hinge region, extended aromatic skeletons and more *N* heteroatoms were used in the optimization strategy. Unfortunately, the experimental results show that this strategy was not ideal. Compounds with the ever-reported dominant side chain substituents still showed poor enzyme and cell inhibitory activities. It could be concluded that the change of aromatic skeleton would significantly affect the binding ability with receptors and further affected the biological activity of compounds. Therefore, in the subsequent structural optimization, the quinoline skeleton was selected from Omipalisib and used for the following research.

According to reported research [15,16,17], it could be concluded that the introduction of amides and aromatic heterocycles can improve the ligand affinity with receptors. Preliminary calculation research also showed that part C possessed a combining cavity with moderate volume and length, which could accommodate an aromatic heterocycle with an amide. In this paper, the oxazole group with a carboxylic acid ester was creatively introduced as the main fragment at part C. It could effectively occupy the ribose binding pocket and formed a π−π interaction with the amino acids, which may further enhance the affinity with the receptor. In addition, the introduced carboxylic acid ester could be easily converted into various amides as our target compounds. As expected, the hydrophilic group of the introduced amide could not only effectively enhance water solubility and metabolic stability, but also enrich the diversity of the compounds. Herein, a series of new compounds were synthesized.

### 2.2. Structure−Activity Relationship (SAR)

ADP-Glo^TM^ kinase assay and cell viability assay were employed to screen our target compounds. Compared with HS-173 or Omipalisib, the **11a–l** and **17a–l** compounds showed poor PI3Kα enzyme inhibitory activity and cell proliferation inhibitory activity (as shown in Table 1 and Table 2).

The negative screening results revealed that the strategies of extension of the aromatic skeleton, as in **11a–l**, and the skeleton with more nitrogen atoms, as in **17a–l**, should be tentatively used in this study.

For **22a–l**, the substituent at the fifth position of oxazole performed crucial effect on the biological activity (as shown in Table 3). The ester showed poor enzyme inhibitory activity while the amides performed enzyme inhibitory activity, which indicated that amide substituents were beneficial to ligand–receptor interaction. Compounds with *N*-alkyl amides of moderate volume showed ideal inhibitory activity, with isopropyl group as the best, followed by cyclopropyl group. Too small or large alkyl groups would lead to poor inhibitory activity. It was indicated that the ribose binding pocket could not hold a large volume of substituents, and the small volume of alkyl substituents could not fully fill it either, leading to a significant decline in enzyme inhibitory activity. Compared with HS-173 or Omipalisib, **22c** also performed potent enzyme inhibitory activity against mTOR (as shown in Table 4). Most compounds had the same inhibition tendency for the two cell lines with PIK3CA mutation (MCF-7 and HCT-116). Furthermore, most compounds had ClogP values of 4–6, indicating ideal lipid water partition coefficients in order to facilitate cell uptake.

### 2.3. Biological Evaluation

**Figure 3:**
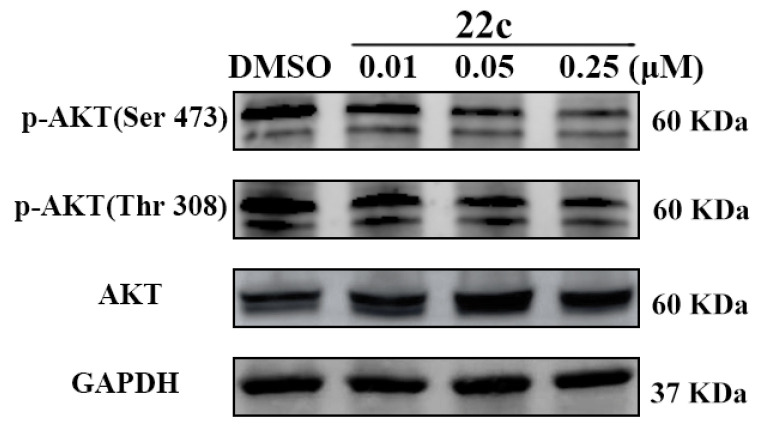
Compound **22c** inhibited phosphorylation of PI3K downstream effectors AKT in HCT-116 cells.

**Figure 4:**
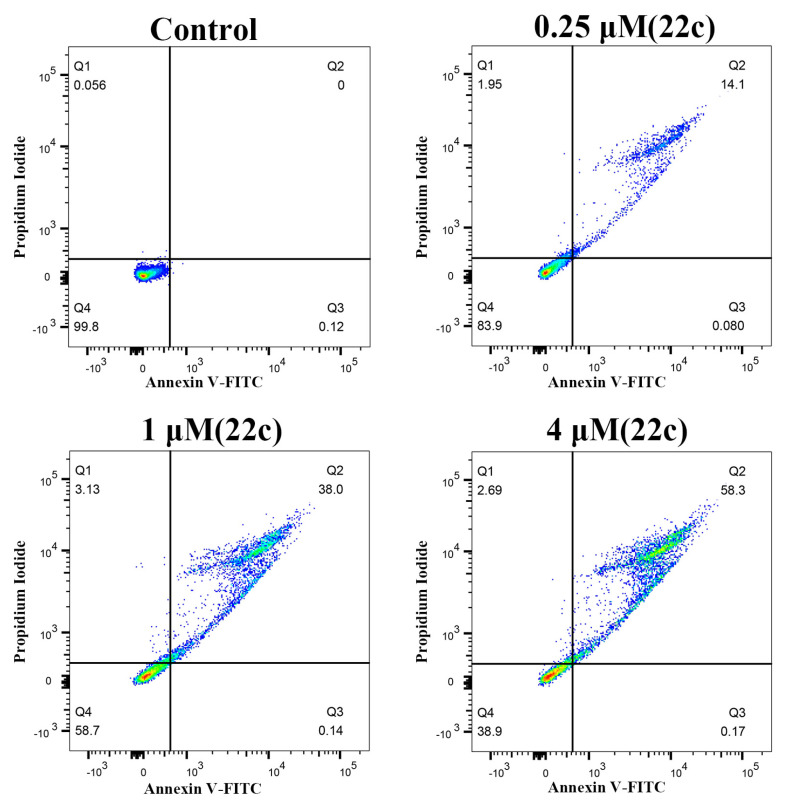
Flow cytometry analysis of apoptosis via Annexin V-FITC/PI staining after treatment with compound **22c** at indicated concentrations for 24 h in HCT−116 cells.

**Figure 5:**
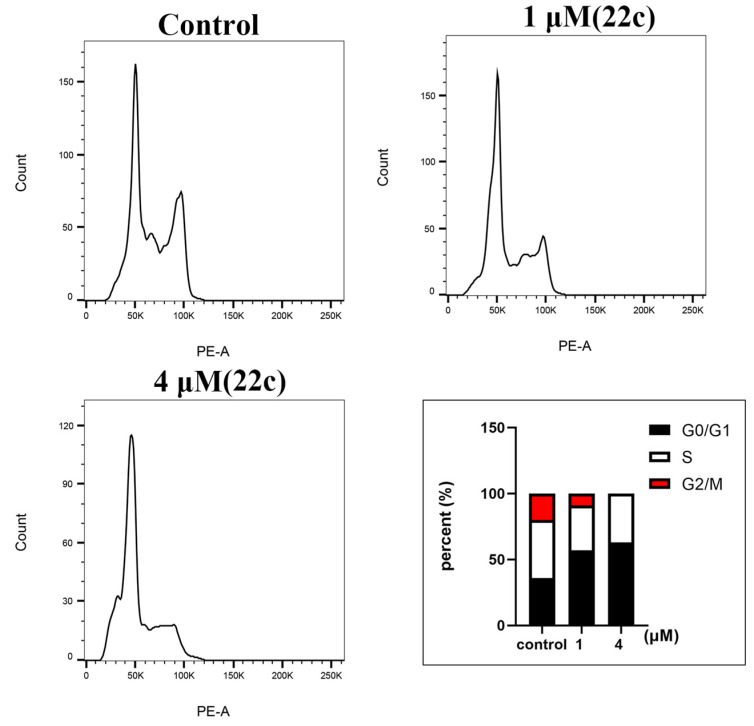
Flow cytometry analysis of cell cycle arrest of HCT-116 cells via PI staining after treatment with compound **22c** at indicated concentration for 24 h.

**Figure 6:**
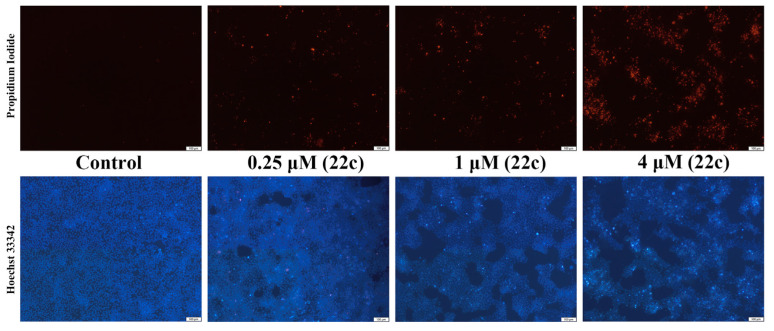
Analysis of the survival and apoptosis of HCT-116 cells induced by **22c** via Hoechst 33342/PI staining. Normal cells show weak red fluorescence and weak blue fluorescence, while apoptotic cells show weak red fluorescence and strong blue fluorescence, the necrotic cells show strong red fluorescence and strong blue fluorescence.

In order to further clarify its anti-tumor mechanism, Western blot analysis was used to confirm the anti-tumor mechanism of **22c**. As shown in Figure 3, **22c** could block the phosphorylation process of AKT at low concentrations, thereby blocking the PI3K/AKT/mTOR signal pathway efficiently. Next, the cell cycle arrest and apoptosis were analyzed by flow cytometry as shown in Figure 4 and Figure 5. **22c** could induce apoptosis and inhibited the cell cycle in G0/G1 phase of HCT-116 cells in a dose-dependent manner. Finally, Hoechst33342/PI staining was used to confirm that **22c** could significantly affect apoptosis and necrosis of HCT-116 cells (as shown in Figure 6). The above experiments proved that **22c** could exert anti-tumor effect by blocking PI3K/AKT/mTOR signal pathway.

## 3. Chemistry

The target compounds could be formed via the Suzuki coupling of the borate of part A and the bromo compound consisting of parts B and C for the sake of the construction of the key carbon–carbon bond. For the synthesis of the borate, 2,4-difluorobenzenesulfonyl chloride was condensed with 5-bromo-2-methoxypyridin-3-amine (**3**) [18], and further converted to the borate ester **5** [19] via Miyaura borylation.

**Scheme 1:**
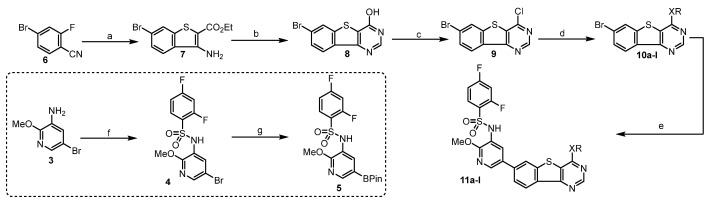
The synthesis of **11a–l**. Reagents and conditions: (**a**) ethyl 2-mercaptoacetate, DIPEA, K_2_CO_3_, DMF, 80 °C for 20 h, 98%; (**b**) formamidine acetate, formamide, 150 °C for 6 h, 95%; (**c**) DMF, POCl_3_, 90 °C for 20 h, 83%; (**d**) nucleophiles, Et_3_N, THF, rt, 4 h; (**e**) **5**, PdCl_2_(dppf)•DCM, K_2_CO_3_, 1,4-dioxane, reflux, 4 h, 55~83%; (**f**) 2,4-difluorobenzenesulfonyl chloride, pyridine, rt, 24 h, 91%; (**g**) bis(pinacolato)diborane, PdCl_2_(dppf)•DCM, KOAc, 1,4-dioxane, reflux, 4 h, 94%.

Respective routes were employed to synthesize our corresponding three types of target compounds. For the first type, the synthesis of **11a–l** was shown in Scheme 1. Compound **8** was prepared via the cyclization of 4-bromo-2-fluorophenylnitrile (**6**) with ethyl 2-mercaptoacetate and the successive cyclization with formamidine [20], and it was further treated with POCl_3_ [21] to give chloride **9**. **9** was converted to the key intermediates **10a–l** via nucleophilic substitution with various nucleophiles, mostly amines. Finally, the target compounds **11a–l** were synthesized via Suzuki–Miyaura coupling.

**Scheme 2:**
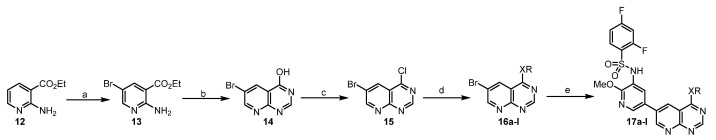
The synthesis of **17a–l**. Reagents and conditions: (**a**) NBS, MeCN, 0 °C for 24 h, 86%; (**b**) formamide, 155 °C for 20 h, 65%; (**c**) DMF, POCl_3_, 90 °C for 20 h, 77%; (**d**) amines, Et_3_N, THF, rt, 4 h, 75~93%; (**e**) **5**, PdCl_2_(dppf)•DCM, K_2_CO_3_, 1,4-dioxane, reflux, 4 h, 58~77%.

For the second type, the synthesis of **17a–l** was shown in Scheme 2. Arylester **12** was brominated and further cyclized with formamide to obtain **14**, which was further converted to **17a–l** using the above method.

**Scheme 3:**
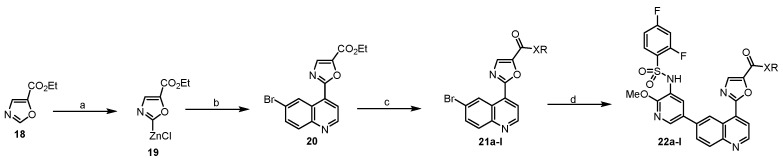
The synthesis of **22a–l**. Reagents and conditions: (**a**) LiHMDS, ZnCl_2_, THF, −10 °C for 1 h; (**b**) 6-bromo-4-iodoquinoline, Pd(PPh_3_)_4_, THF, 60 °C for 15 h, 71%; (**c**) amines, sealed tube, 100 °C for 4 h, 67~85%; (**d**) **5**, PdCl_2_(dppf)•DCM, K_2_CO_3_, 1,4-dioxane, reflux, 4 h, 46~71%.

For the third type, the synthesis of **22a–l** was shown in Scheme 3. **18** was deprotonated with LiHMDS and converted to the organic zinc **19** in THF. **19** was brought to the key intermediate **20** by coupling with 6-bromo-4-iodoquinoline via Negishi reaction [22], which was similarly transformed into **22a–l**.

## 4. Molecular Docking

**Figure 7:**
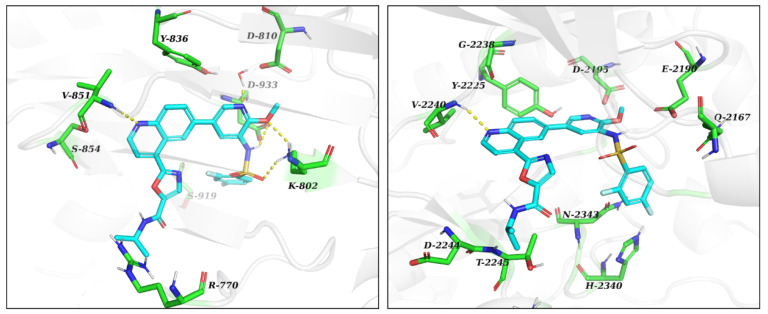
Predicted binding modes between **22c** and the PI3Kα (**left**, PDB code for PI3Kα is 4JPS), mTOR (**right**, PDB code for mTOR is 4JT6). Hydrogen bonds are shown as yellow dashed lines. Images were generated with PyMOL.

In order to further confirm the ligand–receptor interaction, molecular docking was carried out in AutoDock software. In the binding mode between **22c** and PI3Kα (PDB code: 4JPS), the key hydrogen bonds mainly existed in the affinity binding pocket and hinge area binding pocket, which were described as follows (as shown in Figure 7).

In the affinity binding pocket, the oxygen atoms of methoxy and sulfonyl formed conservative hydrogen bonds with Lys802; the *N* atom on the pyridine ring exhibited hydrogen binding with Asp810, Tyr836, and Asp933 through a water molecule. Then, NH of sulfonamide formed a hydrogen bond with Asp933 again. It is very rare to ever report these kinds of multiple hydrogen interactions in the binding pocket.

In the hinge binding pocket, the *N* atom on the quinoline skeleton formed a conservative hydrogen bond with Val851, which was necessary for maintaining enzymatic potency against PI3Kα. In addition, the benzene ring in the quinoline skeleton also formed a π−π interaction with Tyr836. Although the linker oxazole only formed a weak π−π interaction with Trp780, biological evaluation showed that oxazole linker and amide substituents could make great contributions to the kinase inhibitory activity. It could be inferred that there may be other interactions in the ribose binding pocket.

In the binding mode between **22c** and mTOR (PDB code: 4JT6), the *N* atom on the quinoline core formed a critical hydrogen bond with Val2240. The binding model proposed above could support the effect of **22c** on PI3Kα and mTOR kinase.

## 5. Materials and Methods

The reagents and solvents used in this article were chemically pure or analytically pure, and could be used directly without purification. The silica gel used for flash column chromatography and thin layer chromatography was purchased from Qingdao Yumingyuan Silicone Chemical Factory (Qingdao, China). The NMR spectra was recorded by a Bruker AV-400/600 nuclear magnetic resonance instrument, in which TMS was used as the internal standard, the chemical shift is expressed in ppm(δ), and the coupling constant (*J*) is expressed in Hertz (Hz). The HRMS spectrum was determined by an Agilent Accurate Mass Q-TOF 6530 mass spectrometer (Agilent, Santa Clara, CA, USA).

### 5.1. Chemistry

General Procedure A was used for the synthesis of **11a–l**.

The synthesis of *N*-(5-bromo-2-methoxypyridin-3-yl)-2,4-difluorobenzenesulfonamide (**4**). To a solution of 5-bromo-2-methoxypyridin-3-amine (**3**) (4.04 g, 20 mmol) in pyridine (anhydrous, 50 mL), 2,4-difluorobenzenesulfonyl chloride (3.22 mL, 24 mmol) was added dropwise. Then it was stirred at room temperature for 24 h. The solvent was evaporated, followed by addition of H_2_O (100 mL), and stirring for 1 h. The precipitate was filtered, washed with hexane, and dried to give a brown yellow solid (**4**) (6.91 g, 91%).

The synthesis of 2,4-difluoro-*N*-(2-methoxy-5-(4,4,5,5-tetramethyl-1,3,2-dioxaborolan-2-yl)pyridin-3-yl)benzenesulfonamide (**5**). PdCl_2_(dppf)•DCM (204 mg, 0.25 mmol) was added to a mixture of bromo **4** (1.99 g, 5 mmol), KOAc (1.23 g, 7.5 mmol), bis(pinacolato)diborane (1.65 g, 4.5 mmol), and 1,4-dioxane (anhydrous). It was refluxed for 4 h under argon. The solvent was evaporated, diluted with DCM, and washed with H_2_O. The separated organic layer was evaporated in vacuum and the residue was purified by flash column chromatography (Hexane: ethyl acetate = 3:1) to obtain an off-white solid (**5**) (2.00 g, 94%). ^1^H NMR (400 MHz, DMSO-*d*_6_) δ 10.15 (s, 1H), 8.20 (d, *J* = 1.8 Hz, 1H), 7.71 (d, *J* = 1.8 Hz, 1H), 7.68 (dd, *J* = 8.6, 6.3 Hz, 1H), 7.61–7.49 (m, 1H), 7.29–7.11 (m, 1H), 3.62 (s, 3H), 1.29 (s, 12H).

The synthesis of ethyl 3-amino-6-bromobenzo[*b*]thiophene-2-carboxylate (**7**). Ethyl 2-mercaptoacetate (2.20 mL, 20 mmol) was added to a mixture of 4-bromo-2-fluorobenzonitrile (**6**) (4.00 g, 20 mmol), DIPEA (3.30 mL, 20 mmol), K_2_CO_3_ (2.80 g), and DMF (50 mL). The mixture was stirred at 80 °C for 20 h. It was cooled to room temperature and treated with ice water (50 mL) and stirred for 1 h. The formed precipitate was washed with H_2_O (20 mL) and filtered to give a faint yellow solid (**7**) (5.88 g, 98%).^1^H NMR (400 MHz, DMSO-*d*_6_) δ 8.13 (d, *J* = 1.8 Hz, 1H), 8.07 (d, *J* = 8.6 Hz, 1H), 7.57 (dd, *J* = 8.6, 1.8 Hz, 1H), 7.16 (s, 2H), 4.26 (q, *J* = 7.1 Hz, 2H), 1.29 (t, *J* = 7.1 Hz, 3H).

The synthesis of 7-bromobenzo[4,5]thieno[3,2-*d*]pyrimidin-4-ol (**8**). A mixture of the ester **7** (4.50 g, 15 mmol) and formamidine acetate (1.56 g 15 mmol) in formamide (50 mL) was stirred at 150 °C for 45 min. Then an additional portion of formamidine acetate (1.56 g, 15 mmol) was added every 45 min to the mixture. The total formamidine acetate required was 12.48 g, 120 mmol, and the mixture was stirred for 6 h. It was cooled, treated with ice water (100 mL), and stirred for 1 h. The precipitate was filtered, washed with H_2_O, and dried to obtain an ashy solid (3.99 g, 95%).^1^H NMR (400 MHz, DMSO-*d*_6_) δ 12.94 (s, 1H), 8.50 (d, *J* = 1.8 Hz, 1H), 8.35 (s, 1H), 8.15 (d, *J* = 8.5 Hz, 1H), 7.75 (dd, *J* = 8.5, 1.8 Hz, 1H).

The synthesis of 7-bromo-4-chlorobenzo[4,5]thieno[3,2-*d*]pyrimidine (**9**). A mixture of compound **8** (2.80 g, 10 mmol), phosphorous oxychloride (30 mL) and *N*,*N*-dimethylformamide (0.1 mL) was stirred under argon at 90 °C for 20 h. The phosphorous oxychloride was removed in vacuo, and the residue was diluted with DCM and treated with ice water (10 mL). Then it was carefully neutralized with NaHCO_3_, and the formed crude was filtered, washed with H_2_O, and purified with flash column chromatography (Hexane: ethyl acetate = 1:1) to give a yellow solid (2.47 g, 83%).

The synthesis of 4-(7-bromobenzo[4,5]thieno[3,2-*d*]pyrimidin-4-yl)morpholine (**10a**). Morpholine (146 μL, 1 mmol) was added to a mixture of chloride **9** (75 mg, 0.25 mmol) and Et_3_N (70 μL, 0.5 mmol) in THF (5 mL) under an ice cooling bath. Then, the mixture was gradually raised to room temperature, and stirred for additional 4 h. It was concentrated in vacuo and the residue was stirred in ethyl acetate (5 mL) and H_2_O (5 mL). The organic layer was separated and dried. The solvent was evaporated in vacuum to afford **10a** as an ashy solid (89 mg, 88%), which was used without further purification.

**10b−l** were prepared by a similar procedure described for the synthesis of **10a**. The synthesis of 2,4-difluoro-*N*-(2-methoxy-5-(4-morpholinobenzo[4,5]thieno[3,2-*d*]pyrimidin-7-yl)pyridin-3-yl)benzenesulfonamide (**11a**). Under argon protection, PdCl_2_(dppf)•DCM (21 mg, 0.025 mmol) was added to a mixture of bromo compound **10a** (89 mg, 0.22 mmol), borate **5** (213 mg, 0.5 mmol), K_2_CO_3_ (52 mg, 0.375 mmol) in 1,4-dioxane (10 mL), and H_2_O (2 mL). The mixture was refluxed for 4 h. Then it was concentrated and purified by flash column chromatography (hexane: ethyl acetate = 1:1) to obtain the coupled **11a** as a white solid (94 mg, 75%). ^1^H NMR (400 MHz, DMSO-*d_6_*) δ 10.35 (s, 1H), 8.69 (s, 1H), 8.47 (d, J = 2.3 Hz, 1H), 8.47–8.26 (m, 2H), 8.04 (d, J = 2.3 Hz, 1H), 7.85 (dd, J = 8.3, 1.7 Hz, 1H), 7.78 (td, J = 8.6, 6.3 Hz, 1H), 7.58 (ddd, J = 10.6, 9.2, 2.5 Hz, 1H), 7.21 (tt, J = 8.7, 1.6 Hz, 1H), 3.95 (dd, J = 5.7, 4.0 Hz, 4H), 3.79 (dd, J = 5.7, 3.9 Hz, 4H), 3.67 (s, 3H). ^13^C NMR (100 MHz, DMSO-*d6*) δ 170.80, 164.35, 161.21, 158.65, 158.52, 158.41, 158.28, 157.18, 154.80, 143.31, 140.41, 138.47, 134.87, 132.99, 132.40, 132.30, 129.39, 125.64, 125.49, 124.58, 124.29, 120.91, 120.21, 114.30, 112.40, 112.21, 106.53, 106.28, 106.02, 66.46, 60.21, 53.94, 46.50, 21.22, 14.55. ^1^H and ^13^C NMR spectra were shown in Figure S1. HRMS (ESI) *m*/*z*: [M + H]^+^ calcd for C_26_H_22_F_2_N_5_O_4_S_2_, 570.1003; found, 570.1069.

**11b−l** were prepared by a similar procedure described for the synthesis of **11a**.

*N*-(2-methoxy-5-(4-(4-methylpiperidin-1-yl)benzo[4,5]thieno[3,2-d]pyrimidin-7-yl)pyridin-3-yl) 2,4-difluorobenzenesulfonamide (**11b**): flash column chromatography (hexane: ethyl acetate = 1:1) as a white solid (99 mg, 68%). ^1^H NMR (400 MHz, DMSO-*d*_6_) δ 10.34 (s, 1H), 8.63 (s, 1H), 8.47 (d, *J* = 2.3 Hz, 1H), 8.40 (d, *J* = 1.6 Hz, 1H), 8.38 (d, *J* = 8.3 Hz, 1H), 8.03 (d, *J* = 2.4 Hz, 1H), 7.83 (dd, *J* = 8.3, 1.6 Hz, 1H), 7.81–7.74 (m, 1H), 7.58 (ddd, *J* = 10.5, 9.2, 2.5 Hz, 1H), 7.21 (td, *J* = 8.6, 2.7 Hz, 1H), 4.71 (d, *J* = 13.3 Hz, 2H), 3.67 (s, 3H), 3.26–3.15 (m, 2H), 1.80 (d, *J* = 11.7 Hz, 3H), 1.39–1.10 (m, 2H), 0.94 (d, *J* = 6.1 Hz, 3H). ^13^C NMR (100 MHz, DMSO-*d6*) δ 166.88, 166.76, 164.35, 164.24, 161.21, 161.07, 158.65, 158.51, 158.23, 157.87, 156.95, 154.95, 143.23, 140.26, 138.28, 134.77, 133.23, 132.41, 132.30, 129.44, 125.64, 125.61, 125.50, 125.46, 124.48, 124.23, 120.87, 120.23, 113.91, 112.44, 112.40, 112.22, 112.18, 106.53, 106.27, 106.02, 60.21, 53.94, 47.47, 46.60, 34.33, 33.34, 31.00, 22.03, 21.22, 14.55. ^1^H and ^13^C NMR spectra were shown in Figure S2. HRMS (ESI) *m*/*z*: [M – H]^–^ calcd for C_28_H_24_F_2_N_5_O_3_S_2_, 580.1367; found, 580.1343.

*N*-(5-(4-((2-hydroxyethyl)amino)benzo[4,5]thieno[3,2-d]pyrimidin-7-yl)-2-methoxypyridin-3-yl) -2,4-difluoro-benzenesulfonamide (**11c**): flash column chromatography (hexane: ethyl acetate = 1:1) as a white solid (80 mg, 59%). ^1^H NMR (400 MHz, DMSO-*d*_6_) δ 10.34 (s, 1H), 8.60 (s, 1H), 8.49 (d, *J* = 2.4 Hz, 1H), 8.47 (d, *J* = 1.5 Hz, 1H), 8.35 (d, *J* = 8.3 Hz, 1H), 8.05 (d, *J* = 2.3 Hz, 1H), 7.97–7.91 (m, 1H), 7.83 (dd, *J* = 8.3, 1.6 Hz, 1H), 7.77 (td, *J* = 8.6, 6.3 Hz, 1H), 7.58 (ddd, *J* = 10.4, 9.2, 2.5 Hz, 1H), 7.21 (td, *J* = 8.5, 2.5 Hz, 1H), 4.91–4.58 (m, 1H), 3.66 (s, 3H), 3.65–3.61 (m, 4H). ^13^C NMR (100 MHz, DMSO-*d*_6_) δ 166.80 (d, *J* = 12.1 Hz), 164.27 (d, *J* = 11.7 Hz), 161.15 (d, *J* = 13.4 Hz), 158.59 (d, *J* = 13.6 Hz), 158.27, 157.64, 155.47, 154.90, 143.24, 140.83, 137.77, 135.03, 133.74, 132.34 (d, *J* = 10.8 Hz), 129.51, 125.54, 124.12 (d, *J* = 25.9 Hz), 121.78, 120.22, 115.22, 112.29 (d, *J* = 22.4 Hz), 106.26 (t, *J* = 26.1 Hz), 59.85, 53.92, 43.66. ^1^H and ^13^C NMR spectra were shown in Figure S3. HRMS (ESI) *m*/*z*: [M + H]^+^ calcd for C**_24_**H**_20_**F**_2_**N**_5_**O**_4_**S**_2_**, 544.0847; found, 544.0919.

*N*-(5-(4-(cyclopropylamino)benzo[4,5]thieno[3,2-d]pyrimidin-7-yl)-2-methoxypyridin-3-yl)-2,4-difluorobenzenesulfonamide (**11d**): flash column chromatography (hexane: ethyl acetate = 1:1) as a white solid (105 mg, 78%). ^1^H NMR (400 MHz, DMSO-*d*_6_) δ 10.33 (s, 1H), 8.63 (s, 1H), 8.49 (d, *J* = 2.3 Hz, 1H), 8.45 (d, *J* = 1.6 Hz, 1H), 8.36 (d, *J* = 8.3 Hz, 1H), 8.09 (d, *J* = 3.4 Hz, 1H), 8.05 (d, *J* = 2.4 Hz, 1H), 7.83 (dd, *J* = 8.3, 1.6 Hz, 1H), 7.77 (td, *J* = 8.6, 6.3 Hz, 1H), 7.58 (ddd, *J* = 10.5, 9.2, 2.5 Hz, 1H), 7.24–7.17 (m, 1H), 3.66 (s, 3H), 3.04 (tt, *J* = 7.0, 3.6 Hz, 1H), 0.83 (td, *J* = 7.0, 4.7 Hz, 2H), 0.71–0.65 (m, 2H). ^13^C NMR (100 MHz, DMSO-*d*_6_) δ 166.80 (d, *J* = 11.4 Hz), 164.28 (d, *J* = 11.8 Hz), 161.15 (d, *J* = 13.3 Hz), 158.92, 158.59 (d, *J* = 13.1 Hz), 158.27, 155.48, 155.19, 143.26, 141.05, 137.84, 135.02, 133.55, 132.35 (d, *J* = 11.0 Hz), 129.51, 125.59 (d, *J* = 10.6 Hz), 124.12 (d, *J* = 20.4 Hz), 121.62, 120.20, 114.85, 112.28 (d, *J* = 18.8 Hz), 106.26 (t, *J* = 26.1 Hz), 53.92. ^1^H and ^13^C NMR spectra were shown in Figure S4. HRMS (ESI) *m*/*z*: [M – H]^–^ calcd for C**_25_**H**_18_**F**_2_**N**_5_**O**_3_**S**_2_**, 538.0897; found, 538.0857.

*N*-(2-methoxy-5-(4-((3-methoxypropyl)amino)benzo[4,5]thieno[3,2-d]pyrimidin-7-yl)pyridin-3-yl)-2,4-difluorobenzenesulfonamide (**11e**): flash column chromatography (hexane: ethyl acetate = 1:1) as a white solid (117 mg, 82%). ^1^H NMR (400 MHz, DMSO-*d*_6_) δ 10.33 (s, 1H), 8.60 (s, 1H), 8.48 (d, *J* = 2.4 Hz, 1H), 8.46 (d, *J* = 1.6 Hz, 1H), 8.34 (d, *J* = 8.3 Hz, 1H), 8.04 (d, *J* = 2.4 Hz, 1H), 7.94 (t, *J* = 5.5 Hz, 1H), 7.83 (dd, *J* = 8.3, 1.6 Hz, 1H), 7.77 (td, *J* = 8.6, 6.3 Hz, 1H), 7.57 (ddd, *J* = 11.3, 9.2, 2.5 Hz, 1H), 7.24–7.17 (m, 1H), 3.66 (s, 3H), 3.63–3.56 (m, 2H), 3.43 (t, *J* = 6.2 Hz, 2H), 3.26 (s, 3H), 1.89 (p, *J* = 6.6 Hz, 2H). ^13^C NMR (100 MHz, DMSO-*d*_6_) δ 166.72, 164.25 (d, *J* = 11.7 Hz), 161.15 (d, *J* = 13.6 Hz), 158.59 (d, *J* = 13.3 Hz), 158.27, 157.52, 155.53, 154.86, 143.15, 140.78, 137.78, 134.93, 133.77, 132.34 (d, *J* = 10.8 Hz), 129.51, 125.70, 124.11 (d, *J* = 28.2 Hz), 121.76, 120.34, 115.15, 112.26 (d, *J* = 19.4 Hz), 106.25 (t, *J* = 26.0 Hz), 70.21, 58.40, 53.91. ^1^H and ^13^C NMR spectra were shown in Figure S5. HRMS (ESI) *m*/*z*: [M – H]^–^ calcd for C**_26_**H**_22_**F**_2_**N**_5_**O**_4_**S**_2_**, 572.1160; found, 572.1221.

*N*-(5-(4-((2-aminoethyl)amino)benzo[4,5]thieno[3,2-d]pyrimidin-7-yl)-2-methoxypyridin-3-yl)-2,4-difluorobenzenesulfonamide (**11f**): flash column chromatography (hexane: ethyl acetate = 1:1) as a white solid (87 mg, 64%). ^1^H NMR (400 MHz, DMSO-*d*_6_) δ 8.62 (s, 1H), 8.31 (d, *J* = 8.3 Hz, 1H), 8.24 (d, *J* = 5.0 Hz, 1H), 8.22 (s, 1H), 7.94 (s, 1H), 7.90–7.78 (m, 1H), 7.68 (d, *J* = 2.4 Hz, 1H), 7.63 (d, *J* = 8.4 Hz, 1H), 7.29 (td, *J* = 9.8, 2.6 Hz, 1H), 7.11 (td, *J* = 8.5, 2.6 Hz, 1H), 3.82 (q, *J* = 6.1 Hz, 2H), 3.77 (s, 3H), 3.11 (t, *J* = 6.3 Hz, 2H), 1.89 (s, 2H). ^13^C NMR (100 MHz, DMSO-*d*_6_) δ 160.46, 158.74 (d, *J* = 12.9 Hz), 157.57 (d, *J* = 20.6 Hz), 155.22 (d, *J* = 9.3 Hz), 140.93, 139.89, 133.00, 131.98 (d, *J* = 10.8 Hz), 129.03, 124.16, 123.93, 121.09, 115.38, 111.29 (d, *J* = 21.5 Hz), 105.60 (t, *J* = 26.5 Hz), 63.26, 60.22, 53.44, 49.01. ^1^H and ^13^C NMR spectra were shown in Figure S6. HRMS (ESI) *m*/*z*: [M – H]^–^ calcd for C**_24_**H**_19_**F**_2_**N**_6_**O**_3_**S**_2_**, 543.1006; found, 543.1073.

*N*-(5-(4-(isopropylamino)benzo[4,5]thieno[3,2-*d*]pyrimidin-7-yl)-2-methoxypyridin-3-yl)-2,4-difluorobenzenesulfonamide (**11g**): flash column chromatography (hexane: ethyl acetate = 1:1) as a white solid (108 mg, 80%). ^1^H NMR (400 MHz, DMSO-*d*_6_) δ 10.33 (s, 1H), 8.59 (s, 1H), 8.49 (d, *J* = 2.4 Hz, 1H), 8.46 (d, *J* = 1.6 Hz, 1H), 8.34 (d, *J* = 8.3 Hz, 1H), 8.05 (d, *J* = 2.4 Hz, 1H), 7.83 (dd, *J* = 8.3, 1.6 Hz, 1H), 7.79–7.76 (m, 1H), 7.74 (d, *J* = 7.7 Hz, 1H), 7.58 (ddd, *J* = 10.6, 9.2, 2.5 Hz, 1H), 7.20 (td, *J* = 8.6, 2.5 Hz, 1H), 4.50 (dq, *J* = 13.4, 6.7 Hz, 1H), 3.66 (s, 3H), 1.28 (s, 3H), 1.26 (s, 3H). ^13^C NMR (100 MHz, DMSO-*d*_6_) δ 166.80 (d, *J* = 11.6 Hz), 164.27 (d, *J* = 11.8 Hz), 161.16 (d, *J* = 13.5 Hz), 158.60 (d, *J* = 13.5 Hz), 158.28, 156.80, 155.50, 154.96, 143.26, 140.84, 137.73, 135.05, 133.79, 132.34 (d, *J* = 10.7 Hz), 129.53, 126.31–125.16 (m), 124.07 (d, *J* = 26.0 Hz), 121.73, 120.21, 115.07, 113.36–111.62 (m), 106.25 (t, *J* = 26.0 Hz), 53.90, 42.50, 22.69. ^1^H and ^13^C NMR spectra were shown in Figure S7. HRMS (ESI) *m*/*z*: [M + H]^+^ calcd for C**_25_**H**_22_**F**_2_**N**_5_**O**_3_**S**_2_**, 542.1054; found, 542.1122.

*N*-(5-(4-((2-(dimethylamino)ethyl)amino)benzo[4,5]thieno[3,2-*d*]pyrimidin-7-yl)-2-methoxypyridin-3-yl)-2,4-difluorobenzenesulfonamide (**11h**): flash column chromatography (hexane: ethyl acetate = 1:1) as a white solid (100 mg, 70%). ^1^H NMR (400 MHz, DMSO-*d*_6_) δ 8.61 (s, 1H), 8.34–8.25 (m, 2H), 8.17 (d, *J* = 2.3 Hz, 1H), 7.89 (t, *J* = 3.8 Hz, 2H), 7.79 (td, *J* = 8.6, 6.5 Hz, 1H), 7.72 (dd, *J* = 8.3, 1.6 Hz, 1H), 7.45 (td, *J* = 9.8, 2.5 Hz, 1H), 7.16 (td, *J* = 8.3, 2.5 Hz, 1H), 3.70 (s, 6H), 2.82 (t, *J* = 6.4 Hz, 2H), 2.44 (s, 7H). ^13^C NMR (100 MHz, DMSO-*d*_6_) δ 163.53 (d, *J* = 11.9 Hz), 160.93, 158.51, 157.66 (d, *J* = 40.9 Hz), 155.19 (d, *J* = 34.8 Hz), 140.67, 139.58, 138.50, 133.37, 132.16 (d, *J* = 10.4 Hz), 131.19, 128.99, 127.64, 124.76, 123.97 (d, *J* = 27.1 Hz), 121.32, 115.05, 111.81 (d, *J* = 24.9 Hz), 105.95 (t, *J* = 26.2 Hz), 57.65, 53.65, 44.85, 38.13. ^1^H and ^13^C NMR spectra were shown in Figure S8. HRMS (ESI) *m*/*z*: [M + H]^+^ calcd for C_26_H_25_F_2_N_6_O_3_S_2_, 570.1319; found, 570.1388.

*N*-(5-(4-(butylamino)benzo[4,5]thieno[3,2-*d*]pyrimidin-7-yl)-2-methoxypyridin-3-yl)-2,4-difluorobenzenesulfonamide (**11i**): flash column chromatography (hexane: ethyl acetate = 1:1) as a white solid (80 mg, 58%). ^1^H NMR (400 MHz, DMSO-*d*_6_) δ 10.39 (s, 1H), 8.59 (s, 1H), 8.49 (d, *J* = 2.3 Hz, 1H), 8.46 (d, *J* = 1.6 Hz, 1H), 8.34 (d, *J* = 8.3 Hz, 1H), 8.05 (d, *J* = 2.4 Hz, 1H), 7.93 (t, *J* = 5.6 Hz, 1H), 7.85–7.81 (m, 1H), 7.77 (td, *J* = 8.6, 6.3 Hz, 1H), 7.61–7.53 (m, 1H), 7.21 (td, *J* = 8.6, 2.5 Hz, 1H), 3.66 (s, 3H), 3.58–3.50 (m, 2H), 1.63 (s, 2H), 1.38 (d, *J* = 7.4 Hz, 2H), 0.93 (t, *J* = 7.4 Hz, 3H). ^13^C NMR (100 MHz, DMSO-*d*_6_) δ 166.80 (d, *J* = 11.9 Hz), 164.27 (d, *J* = 11.7 Hz), 161.15 (d, *J* = 13.3 Hz), 158.59 (d, *J* = 13.5 Hz), 158.26, 157.53, 155.55, 154.84, 143.22, 140.78, 137.73, 134.97, 133.81, 132.35 (d, *J* = 10.9 Hz), 129.53, 126.66–125.11 (m), 124.23, 123.96, 121.76, 120.24, 115.08, 112.27 (dd, *J* = 22.1, 3.5 Hz), 106.25 (t, *J* = 26.1 Hz), 91.15, 73.99, 70.40, 65.65, 63.19, 60.21, 53.91, 31.45, 20.17, 14.24. ^1^H and ^13^C NMR spectra were shown in Figure S9. HRMS (ESI) *m*/*z*: [M – H]^–^ calcd for C**_26_**H**_22_**F**_2_**N**_5_**O**_3_**S**_2_**, 554.1210; found, 554.1178.

*N*-(2-methoxy-5-(4-((3-morpholinopropyl)amino)benzo[4,5]thieno[3,2-*d*]pyrimidin-7-yl)pyridin-3-yl)-2,4-difluorobenzenesulfonamide (**11j**): flash column chromatography (hexane: ethyl acetate = 1:1) as a white solid (92 mg, 59%). ^1^H NMR (400 MHz, DMSO-*d*_6_) δ 10.38 (s, 1H), 8.60 (s, 1H), 8.46 (t, *J* = 2.1 Hz, 2H), 8.34 (d, *J* = 8.3 Hz, 1H), 8.03 (d, *J* = 2.4 Hz, 1H), 7.97 (t, *J* = 5.4 Hz, 1H), 7.82 (dd, *J* = 8.4, 1.7 Hz, 1H), 7.80–7.73 (m, 1H), 7.56 (ddd, *J* = 11.4, 9.3, 2.5 Hz, 1H), 7.20 (td, *J* = 8.5, 2.5 Hz, 1H), 3.67 (s, 3H), 3.61 (s, 2H), 2.44 (d, *J* = 7.2 Hz, 3H), 2.41 (d, *J* = 4.3 Hz, 3H), 1.82 (s, 1H). ^13^C NMR (100 MHz, DMSO-*d*_6_) δ 166.68 (d, *J* = 11.7 Hz), 164.16 (d, *J* = 11.6 Hz), 161.14 (d, *J* = 13.6 Hz), 158.58 (d, *J* = 13.5 Hz), 158.25, 157.49, 155.56, 154.86, 142.69, 140.70, 137.91, 134.46, 133.75, 132.32 (d, *J* = 10.6 Hz), 129.47, 125.92 (d, *J* = 14.3 Hz), 124.12 (d, *J* = 29.1 Hz), 121.75, 120.93, 115.11, 112.22 (d, *J* = 22.3 Hz), 106.21 (t, *J* = 26.1 Hz), 67.48, 66.58, 56.62, 53.82 (d, *J* = 10.4 Hz), 25.71 (d, *J* = 23.9 Hz). ^1^H and ^13^C NMR spectra were shown in Figure S10. HRMS (ESI) *m*/*z*: [M – H]^–^ calcd for C**_29_**H**_27_**F**_2_**N**_6_**O**_4_**S**_2_**, 625.1582; found, 625.1555.

*N*-(5-(4-(2-(dimethylamino)ethoxy)benzo[4,5]thieno[3,2-*d*]pyrimidin-7-yl)-2-methoxypyridin-3-yl)-2,4-difluorobenzenesulfonamide (**11k**): flash column chromatography (hexane: ethyl acetate = 1:1) as a white solid (104 mg, 73%). ^1^H NMR (400 MHz, DMSO-*d*_6_) δ 8.90 (s, 1H), 8.42 (d, *J* = 8.5 Hz, 2H), 8.32 (d, *J* = 2.3 Hz, 1H), 7.96 (d, *J* = 2.4 Hz, 1H), 7.83 (dd, *J* = 8.2, 1.8 Hz, 1H), 7.81–7.76 (m, 1H), 7.51 (d, *J* = 2.0 Hz, 1H), 7.19 (td, *J* = 8.5, 2.5 Hz, 1H), 4.73 (t, *J* = 5.6 Hz, 2H), 3.69 (s, 3H), 2.92 (t, *J* = 5.6 Hz, 2H), 2.38 (s, 6H). ^13^C NMR (100 MHz, DMSO-*d*_6_) δ 166.44 (d, *J* = 11.5 Hz), 164.07, 163.93 (d, *J* = 11.5 Hz), 161.15, 158.53 (d, *J* = 13.1 Hz), 158.16 (d, *J* = 5.1 Hz), 155.15, 141.59 (d, *J* = 30.1 Hz), 139.17, 132.78 (d, *J* = 40.9 Hz), 132.25 (d, *J* = 10.7 Hz), 129.01, 126.59 (d, *J* = 14.8 Hz), 124.49 (d, *J* = 34.5 Hz), 122.59, 121.79, 117.52, 112.09 (d, *J* = 21.6 Hz), 106.12 (t, *J* = 26.2 Hz), 64.92, 57.37, 53.82, 45.52. ^1^H and ^13^C NMR spectra were shown in Figure S11. HRMS (ESI) *m*/*z*: [M + H]^+^ calcd for C**_26_**H**_24_**F**_2_**N**_5_**O**_4_**S**_2_**, 572.1160; found, 572.1229.

*N*-(2-methoxy-5-(4-(2-methoxyethoxy)benzo[4,5]thieno[3,2-*d*]pyrimidin-7-yl)pyridin-3-yl)-2,4-difluorobenzenesulfonamide (**11l**): flash column chromatography (hexane: ethyl acetate = 1:1) as a white solid (102 mg, 73%). ^1^H NMR (400 MHz, DMSO-*d*6) δ 10.35 (s, 1H), 8.91 (s, 1H), 8.52 (d, *J* = 1.6 Hz, 1H), 8.49 (d, *J* = 2.3 Hz, 1H), 8.45 (d, *J* = 8.3 Hz, 1H), 8.06 (d, *J* = 2.4 Hz, 1H), 7.90 (dd, *J* = 8.3, 1.6 Hz, 1H), 7.78 (td, *J* = 8.6, 6.3 Hz, 1H), 7.58 (ddd, *J* = 10.5, 9.2, 2.5 Hz, 1H), 7.21 (td, *J* = 8.6, 2.5 Hz, 1H), 4.82–4.66 (m, 2H), 3.87–3.74 (m, 2H), 3.67 (s, 3H), 3.35 (s, 3H). ^13^C NMR (100 MHz, DMSO-*d*6) δ 166.80 (d, *J* = 11.9 Hz), 164.34, 164.19, 161.15 (d, *J* = 13.5 Hz), 158.58 (d, *J* = 13.5 Hz), 158.38, 158.15, 155.20, 143.30, 141.76, 138.85, 134.91, 132.73, 132.34 (d, *J* = 11.0 Hz), 129.21, 126.07–125.29 (m), 124.76, 124.40, 122.03, 120.33, 117.53, 112.30 (d, *J* = 25.0 Hz), 106.27 (t, *J* = 26.1 Hz), 70.28, 66.73, 58.72, 53.96. ^1^H and ^13^C NMR spectra were shown in Figure S12. HRMS (ESI) *m*/*z*: [M – H]^–^ calcd for C_25_H_19_F_2_N_4_O_5_S_2_, 557.0543; found, 557.0820.

General Procedure B was used for the synthesis of **17a–l**.

The synthesis of ethyl 2-amino-5-bromonicotinate (**13**). 1-bromo-2,5-pyrrolidinedione (13.8 g in 15 mL acetonitrile, 78 mmol) was added to a mixture of compound **12** (9.9 g, 60 mmol) in acetonitrile (50 mL) under ice cooling bath. The mixture was gradually raised to room temperature and stirred for an additional 1 h. The precipitate was filtered, washed with dilute ammonia, and dried to give a white solid (**13**) (14.1 g, 86%).

The synthesis of 6-bromopyrido[2,3-*d*]pyrimidin-4-ol (**14**). A mixture of compound **13** (14.1 g, 58 mmol) and formamide (80 mL) was stirred at 155 °C for 20 h. It was cooled down to room temperature and treated with ice water (400 mL). The mixture was stirred for 1 h, filtered and washed with ethyl acetate to give a white solid (**14**) (8.5 g, 65%). ^1^H NMR (400 MHz, DMSO-*d*_6_) δ 12.72 (s, 1H), 9.04 (d, *J* = 2.6 Hz, 1H), 8.61 (d, *J* = 2.6 Hz, 1H), 8.36 (s, 1H).

The synthesis of 6-bromo-4-chloropyrido[2,3-*d*]pyrimidine (**15**). A mixture of compound **14** (2.25 g 10 mmol), phosphorous oxychloride (30 mL), and *N*,*N*-dimethylformamide (0.1 mL) was stirred under argon at 90 °C for 20 h. The phosphorous oxychloride was removed in vacuo, and the residue was diluted with DCM and treated with ice water (10 mL). Then it was carefully neutralized with NaHCO_3_, the formed crude was filtered, washed with water, and purified by flash column chromatography (Hexane: ethyl acetate = 1:1) to give a yellow solid (**15**) (1.9 g, 77%).

The synthesis of 6-bromo-*N*-isopropylpyrido[2,3-*d*]pyrimidin-4-amine (**16a**). Isopropyl amine (86 μL, 1 mmol) was added to a mixture of **15** (75 mg, 0.25 mmol) and Et_3_N (70 μL, 0.5 mmol) in THF (5 mL) under ice cooling bath. Then, the mixture was gradually raised to room temperature, and stirred for an additional 4 h. It was concentrated in vacuo and the residue was stirred in ethyl acetate (5 mL) and H_2_O (5 mL). The organic layer was separated and dried. The solvent was evaporated in vacuum to afford **16a** as an ashy solid (55 mg, 83.1%), which was used without further purification.

**16b−l** were prepared by a similar procedure described for the synthesis of **16a**.

The synthesis of 2,4-difluoro-*N*-(5-(4-(isopropylamino)pyrido[2,3-*d*]pyrimidin-6-yl)-2-methoxypyridin-3-yl)benzenesulfonamide (**17a**). Under argon protection, PdCl_2_(dppf)•DCM (21 mg, 0.025 mmol) was added to a mixture of bromo compound **16a** (55 mg, 0.21mmol), borate **5** (213 mg, 0.5 mmol), K_2_CO_3_ (52 mg, 0.375 mmol) in 1,4-dioxane (10 mL), and H_2_O (2 mL). The mixture was refluxed for 4 h, then concentrated and purified by flash column chromatography (hexane: ethyl acetate = 1:1) to obtain the coupled **17a** as a white solid (90 mg, 88%). ^1^H NMR (400 MHz, DMSO-*d*_6_) δ 10.40 (s, 1H), 9.29 (d, *J* = 2.4 Hz, 1H), 9.03 (d, *J* = 2.5 Hz, 1H), 8.61 (s, 1H), 8.56 (d, *J* = 2.4 Hz, 1H), 8.33 (d, *J* = 7.5 Hz, 1H), 8.14 (d, *J* = 2.4 Hz, 1H), 7.74 (td, *J* = 8.6, 6.2 Hz, 1H), 7.58 (ddd, *J* = 11.4, 9.3, 2.5 Hz, 1H), 7.20 (td, *J* = 8.5, 2.5 Hz, 1H), 4.54 (hept, *J* = 6.7 Hz, 1H), 3.63 (s, 3H), 1.31 (d, *J* = 6.6 Hz, 6H). ^13^C NMR (100 MHz, DMSO-*d*_6_) δ 166.80 (d, *J* = 11.4 Hz), 164.27 (d, *J* = 12.0 Hz), 161.18 (d, *J* = 13.5 Hz), 160.41, 158.80 (d, *J* = 13.0 Hz), 158.55, 158.02, 153.86, 143.54, 135.63, 132.29 (d, *J* = 11.0 Hz), 129.89, 129.51, 126.69, 125.68 (d, *J* = 10.8 Hz), 120.27, 113.22–111.50 (m), 109.78, 106.23 (t, *J* = 26.2 Hz), 60.22, 53.88, 43.12, 22.45. ^1^H and ^13^C NMR spectra were shown in Figure S13. HRMS (ESI) *m*/*z*: [M – H]^–^ calcd for C**_22_**H**_19_**F**_2_**N**_6_**O**_3_**S, 485.1286; found, 485.1231.

**17b–i** were prepared by a similar procedure described for the synthesis of **17a**.

*N*-(5-(4-(cyclopropylamino)pyrido[2,3-*d*]pyrimidin-6-yl)-2-methoxypyridin-3-yl)-2,4-difluorobenzenesulfonamide (**17b**): flash column chromatography (hexane: ethyl acetate = 1:1) as a white solid (80 mg, 65%). ^1^H NMR (400 MHz, DMSO-*d6*) δ 10.37 (s, 1H), 9.31 (d, J = 2.4 Hz, 1H), 8.95 (d, J = 2.5 Hz, 1H), 8.67 (s, 1H), 8.64 (s, 1H), 8.54 (d, J = 2.4 Hz, 1H), 8.12 (d, J = 2.4 Hz, 1H), 7.74 (td, J = 8.6, 6.2 Hz, 1H), 7.57 (ddd, J = 10.5, 9.2, 2.5 Hz, 1H), 7.19 (td, J = 8.5, 2.5 Hz, 1H), 3.63 (s, 3H), 3.07 (dt, J = 7.7, 3.6 Hz, 1H), 0.86 (dt, J = 6.9, 3.4 Hz, 2H), 0.77–0.65 (m, 2H). ^13^C NMR (100 MHz, DMSO-*d6*) δ 166.85, 166.74, 164.33, 164.21, 162.50, 161.24, 161.11, 158.88, 158.75, 158.68, 158.55, 157.77, 153.95, 143.47, 135.58, 132.33, 132.22, 129.77, 129.58, 126.63, 125.78, 125.74, 125.63, 125.60, 120.30, 112.34, 112.31, 112.12, 112.09, 109.75, 106.47, 106.21, 105.96, 60.21, 53.89, 24.95, 14.55, 6.72. ^1^H and ^13^C NMR spectra were shown in Figure S14. HRMS (ESI) *m*/*z*: [M – H]^–^ calcd for C**_22_**H**_17_**F**_2_**N**_6_**O**_3_**S, 483.1129; found, 483.1057.

*N*-(5-(4-(butylamino)pyrido[2,3-*d*]pyrimidin-6-yl)-2-methoxypyridin-3-yl)-2,4-difluorobenzenesulfonamide (**17c**): flash column chromatography (hexane: ethyl acetate = 1:1) as a white solid (75 mg, 60%). ^1^H NMR (400 MHz, DMSO-*d*_6_) δ 10.40 (s, 1H), 9.30 (d, *J* = 2.4 Hz, 1H), 9.00 (d, *J* = 2.5 Hz, 1H), 8.64 (t, *J* = 5.5 Hz, 1H), 8.60 (s, 1H), 8.56 (d, *J* = 2.3 Hz, 1H), 8.13 (d, *J* = 2.4 Hz, 1H), 7.74 (td, *J* = 8.5, 6.2 Hz, 1H), 7.58 (ddd, *J* = 11.3, 9.3, 2.5 Hz, 1H), 7.20 (td, *J* = 8.5, 2.5 Hz, 1H), 3.63 (s, 3H), 3.58 (q, *J* = 7.0 Hz, 2H), 1.66 (p, *J* = 7.3 Hz, 2H), 1.41 (h, *J* = 7.4 Hz, 2H), 0.94 (t, *J* = 7.4 Hz, 3H). ^13^C NMR (100 MHz, DMSO-*d*_6_) δ 166.85, 164.27 (d, *J* = 11.9 Hz), 161.25, 161.12, 158.81 (d, *J* = 14.4 Hz), 157.95, 153.82, 143.45, 135.59, 132.28 (d, *J* = 10.5 Hz), 129.61 (d, *J* = 24.0 Hz), 126.64, 125.70 (d, *J* = 14.6 Hz), 120.31, 112.20 (d, *J* = 22.3 Hz), 109.85, 106.22 (t, *J* = 26.2 Hz), 53.89, 41.11, 31.07, 20.22, 14.24. ^1^H and ^13^C NMR spectra were shown in Figure S15. HRMS (ESI) *m*/*z*: [M – H]^–^ calcd for C**_23_**H**_21_**F**_2_**N**_6_**O**_3_**S, 499.1442; found, 499.1388.

*N*-(5-(4-((2-(dimethylamino)ethyl)amino)pyrido[2,3-*d*]pyrimidin-6-yl)-2-methoxypyridin-3-yl)-2,4-difluorobenzenesulfonamide (**17d**): flash column chromatography (hexane: ethyl acetate = 1:1) as a white solid (87 mg, 68%). ^1^H NMR (400 MHz, DMSO-*d*_6_) δ 10.35–10.02 (m, 1H), 9.31 (d, *J* = 2.4 Hz, 1H), 9.24 (s, 1H), 9.14 (d, *J* = 2.6 Hz, 1H), 8.64 (s, 1H), 8.60 (d, *J* = 2.4 Hz, 1H), 8.12 (d, *J* = 2.4 Hz, 1H), 7.75 (td, *J* = 8.4, 6.1 Hz, 1H), 7.65–7.54 (m, 1H), 7.20 (td, *J* = 8.5, 2.5 Hz, 1H), 3.94 (q, *J* = 5.1 Hz, 2H), 3.63 (s, 3H), 3.43 (t, *J* = 5.7 Hz, 2H), 2.88 (s, 6H). ^13^C NMR (100 MHz, DMSO-d6) δ 166.80, 166.68, 164.28, 164.16, 161.21, 161.07, 158.65, 158.52, 158.26, 157.52, 155.53, 154.93, 142.98, 140.76, 137.86, 134.76, 133.72, 132.39, 132.27, 129.48, 125.79, 125.67, 124.28, 123.99, 121.78, 120.56, 115.14, 112.37, 112.14, 106.50, 106.24, 105.98, 66.59, 60.22, 57.56, 53.90, 53.82, 38.04, 24.95, 21.23, 14.55. ^1^H and ^13^C NMR spectra were shown in Figure S16. HRMS (ESI) *m*/*z*: [M – H]^–^ calcd for C**_23_**H**_22_**F**_2_**N**_7_**O**_3_**S, 514.1551; found, 514.1471.

*N*-(2-methoxy-5-(4-morpholinopyrido[2,3-*d*]pyrimidin-6-yl)pyridin-3-yl)2,4-difluorobenzenesulfonamide (**17e**): flash column chromatography (hexane: ethyl acetate = 1:1) as a white solid (91 mg, 71%). ^1^H NMR (400 MHz, DMSO-*d*_6_) δ 10.36 (s, 1H), 9.28 (d, *J* = 2.5 Hz, 1H), 8.72 (s, 1H), 8.54–8.45 (m, 2H), 8.08 (d, *J* = 2.4 Hz, 1H), 7.84–7.69 (m, 1H), 7.58 (ddd, *J* = 11.4, 9.1, 2.5 Hz, 1H), 7.21 (td, *J* = 8.6, 2.5 Hz, 1H), 3.92 (t, *J* = 4.7 Hz, 4H), 3.77 (t, *J* = 4.7 Hz, 4H), 3.67 (s, 3H). ^13^C NMR (100 MHz, DMSO-*d*_6_) δ 166.83, 164.43, 164.19, 161.05, 159.64, 158.46, 157.00, 154.53, 143.66, 135.03, 133.51–130.58 (m), 129.04, 126.56, 125.70, 120.40, 112.30 (d, *J* = 21.5 Hz), 110.21, 106.28 (t, *J* = 26.1 Hz), 66.44, 53.94, 49.81. ^1^H and ^13^C NMR spectra were shown in Figure S17. HRMS (ESI) *m*/*z*: [M – H]^–^ calcd for C**_23_**H**_19_**F**_2_**N**_6_**O**_4_**S, 513.1235; found, 513.1203.

*N*-(2-methoxy-5-(4-(4-methylpiperidin-1-yl)pyrido[2,3-*d*]pyrimidin-6-yl)pyridin-3-yl)-2,4-difluorobenzenesulfonamide (**17f**): flash column chromatography (hexane: ethyl acetate = 1:1) as a white solid (84 mg, 64%). ^1^H NMR (400 MHz, DMSO-*d*_6_) δ 10.39–10.37 (m, 1H), 9.26 (d, *J* = 2.4 Hz, 1H), 8.66 (s, 1H), 8.48 (d, *J* = 2.4 Hz, 1H), 8.40 (d, *J* = 2.5 Hz, 1H), 8.05 (d, *J* = 2.4 Hz, 1H), 7.77 (td, *J* = 8.6, 6.3 Hz, 1H), 7.57 (ddd, *J* = 11.4, 9.2, 2.5 Hz, 1H), 7.21 (td, *J* = 8.5, 2.5 Hz, 1H), 4.43 (d, *J* = 13.1 Hz, 2H), 3.68 (s, 3H), 3.30 (d, *J* = 14.4 Hz, 3H), 1.77 (t, *J* = 7.8 Hz, 3H), 1.33 (td, *J* = 13.6, 6.8 Hz, 2H), 0.97 (d, *J* = 6.0 Hz, 3H). ^13^C NMR (100 MHz, DMSO-*d*_6_) δ 170.80, 166.72 (d, *J* = 11.2 Hz), 164.39, 164.25, 161.11 (d, *J* = 13.4 Hz), 159.72, 158.55 (d, *J* = 13.6 Hz), 158.31, 157.09, 154.22, 143.04, 134.32, 132.28, 132.18, 128.75, 126.60, 125.82, 120.87, 113.75–111.67 (m), 110.17, 107.45, 106.27 (t, *J* = 26.1 Hz), 67.08, 60.22, 53.94, 49.82, 34.14, 30.87, 22.09, 14.55, 14.42. ^1^H and ^13^C NMR spectra were shown in Figure S18. HRMS (ESI) *m*/*z*: [M – H]^–^ calcd for C**_25_**H**_23_**F**_2_**N**_6_**O**_3_**S, 525.1599; found, 525.1539.

*N*-(2-methoxy-5-(4-((2-morpholinoethyl)amino)pyrido[2,3-*d*]pyrimidin-6-yl)pyridin-3-yl) -2,4-difluorobenzenesulfonamide (**17g**): flash column chromatography (hexane: ethyl acetate = 1:1) as a white solid (96 mg, 69%). ^1^H NMR (400 MHz, DMSO-*d*_6_) δ 10.40 (s, 1H), 9.30 (d, *J* = 2.4 Hz, 1H), 8.99 (d, *J* = 2.5 Hz, 1H), 8.71 (t, *J* = 5.6 Hz, 1H), 8.62 (s, 1H), 8.52 (d, *J* = 2.3 Hz, 1H), 7.76 (td, *J* = 8.5, 6.2 Hz, 1H), 7.20 (td, *J* = 8.5, 2.5 Hz, 1H), 3.73 (q, *J* = 6.5 Hz, 2H), 3.65 (s, 3H), 3.59 (t, *J* = 4.7 Hz, 4H), 2.64 (t, *J* = 6.9 Hz, 2H), 2.56–2.44 (m, 4H). ^13^C NMR (100 MHz, DMSO-*d*_6_) δ 170.80, 161.33, 158.77 (d, *J* = 15.2 Hz), 157.94, 153.94, 142.75, 132.24 (d, *J* = 10.8 Hz), 131.22, 129.70 (d, *J* = 4.1 Hz), 126.56, 113.27–111.12 (m), 109.88, 106.16 (t, *J* = 26.2 Hz), 66.59, 60.21, 57.29, 53.86, 38.58. ^1^H and ^13^C NMR spectra were shown in Figure S19. HRMS (ESI) *m*/*z*: [M – H]^–^ calcd for C**_25_**H**_24_**F**_2_**N**_7_**O**_4_**S, 556.1657; found, 556.1578.

*N*-(5-(4-((2-hydroxyethyl)amino)pyrido[2,3-*d*]pyrimidin-6-yl)-2-methoxypyridin-3-yl)-2,4-difluorobenzenesulfonamide (**17h**): flash column chromatography (hexane: ethyl acetate = 1:1) as a white solid (71 mg, 58%). ^1^H NMR (400 MHz, DMSO-*d*_6_) δ 10.38 (s, 1H), 9.29 (d, *J* = 2.4 Hz, 1H), 9.16 (d, *J* = 2.5 Hz, 1H), 8.90 (d, *J* = 5.4 Hz, 1H), 8.59 (s, 1H), 8.58 (d, *J* = 3.3 Hz, 1H), 8.14 (d, *J* = 2.3 Hz, 1H), 7.75 (td, *J* = 8.5, 6.3 Hz, 1H), 7.63–7.49 (m, 1H), 7.19 (td, *J* = 8.5, 2.5 Hz, 1H), 4.94 (d, *J* = 5.5 Hz, 1H), 3.66 (d, *J* = 2.6 Hz, 4H), 3.63 (s, 3H). ^13^C NMR (100 MHz, DMSO-*d*_6_) δ 161.48, 158.80, 158.70, 157.97, 153.80, 132.28 (d, *J* = 11.1 Hz), 129.99, 129.51, 126.55, 112.19 (d, *J* = 22.1 Hz), 109.99, 106.18, 63.27, 59.52, 53.87, 44.32. ^1^H and ^13^C NMR spectra were shown in Figure S20. HRMS (ESI) *m*/*z*: [M – H]^–^ calcd for C**_21_**H**_19_**F**_2_**N**_6_**O**_4_**S, 487.1078; found, 487.1025.

*N*-(2-methoxy-5-(4-((3-methoxypropyl)amino)pyrido[2,3-*d*]pyrimidin-6-yl)pyridin-3-yl)- 2,4-difluorobenzenesulfonamide (**17i**): flash column chromatography (hexane: ethyl acetate = 1:1) as a white solid (90 mg, 70%). ^1^H NMR (400 MHz, DMSO-*d*_6_) δ 10.43 (s, 1H), 9.28 (d, *J* = 2.4 Hz, 1H), 8.98 (d, *J* = 2.5 Hz, 1H), 8.67 (t, *J* = 5.5 Hz, 1H), 8.60 (s, 1H), 8.51 (d, *J* = 2.3 Hz, 1H), 8.10 (d, *J* = 2.4 Hz, 1H), 7.75 (td, *J* = 8.6, 6.3 Hz, 1H), 7.55 (ddd, *J* = 11.6, 9.3, 2.5 Hz, 1H), 7.19 (td, *J* = 8.6, 2.5 Hz, 1H), 3.64 (s, 3H), 3.61 (d, *J* = 6.9 Hz, 2H), 3.26 (s, 3H), 1.91 (p, *J* = 6.5 Hz, 2H). ^13^C NMR (100 MHz, DMSO-*d*_6_) δ 161.29, 158.84, 158.69, 157.95, 153.84, 132.24 (d, *J* = 10.8 Hz), 129.69, 126.60, 113.46–111.45 (m), 109.89, 106.15 (t, *J* = 26.1 Hz), 70.08, 58.40, 53.84, 38.68, 29.01. ^1^H and ^13^C NMR spectra were shown in Figure S21. HRMS (ESI) *m*/*z*: [M – H]^–^ calcd for C**_23_**H**_21_**F**_2_**N**_6_**O**_4_**S, 515.1391; found, 515.1345.

*N*-(2-methoxy-5-(4-((2-methoxyethyl)amino)pyrido[2,3-*d*]pyrimidin-6-yl)pyridin-3-yl)-2,4-difluorobenzenesulfonamide (**17j**): flash column chromatography (hexane: ethyl acetate = 1:1) as a white solid (93 mg, 74%). ^1^H NMR (400 MHz, DMSO-*d*_6_) δ 10.44–10.31 (m, 1H), 9.32 (d, *J* = 2.4 Hz, 1H), 9.04 (d, *J* = 2.5 Hz, 1H), 8.78 (t, *J* = 5.6 Hz, 1H), 8.64–8.55 (m, 2H), 8.15 (d, *J* = 2.4 Hz, 1H), 7.75 (td, *J* = 8.5, 6.2 Hz, 1H), 7.58 (ddd, *J* = 11.3, 9.3, 2.5 Hz, 1H), 7.20 (td, *J* = 8.5, 2.5 Hz, 1H), 3.77 (q, *J* = 5.5 Hz, 2H), 3.63 (s, 3H), 3.61 (t, *J* = 5.9 Hz, 2H), 3.31 (s, 3H). ^13^C NMR (100 MHz, DMSO-*d*_6_) δ 166.80 (d, *J* = 11.6 Hz), 164.28 (d, *J* = 11.8 Hz), 161.38, 161.18 (d, *J* = 13.6 Hz), 158.75, 157.93, 153.92, 143.45, 135.60, 132.30 (d, *J* = 10.8 Hz), 129.64 (d, *J* = 19.0 Hz), 126.56, 125.67 (d, *J* = 14.5 Hz), 120.28, 112.23 (d, *J* = 20.9 Hz), 109.88, 106.22 (t, *J* = 26.2 Hz), 70.46, 58.54, 53.90, 41.16. ^1^H and ^13^C NMR spectra were shown in Figure S22. HRMS (ESI) *m*/*z*: [M + H]^+^ calcd for C_22_H_21_F_2_N_6_O_4_S, 503.1235; found, 503.1316.

*N*-(5-(4-((cyclohexylmethyl)amino)pyrido[2,3-*d*]pyrimidin-6-yl)-2-methoxypyridin-3-yl)-2,4-difluorobenzenesulfonamide (**17k**): flash column chromatography (hexane: ethyl acetate = 1:1) as a white solid (90 mg, 67%). ^1^H NMR (400 MHz, DMSO-*d*_6_) δ 10.38 (s, 1H), 9.29 (d, *J* = 2.4 Hz, 1H), 9.02 (d, *J* = 2.5 Hz, 1H), 8.65 (t, *J* = 5.7 Hz, 1H), 8.59 (s, 1H), 8.56 (d, *J* = 2.4 Hz, 1H), 8.13 (d, *J* = 2.3 Hz, 1H), 7.75 (td, *J* = 8.5, 6.2 Hz, 1H), 7.57 (ddd, *J* = 10.6, 9.2, 2.5 Hz, 1H), 7.19 (td, *J* = 8.6, 2.5 Hz, 1H), 3.63 (s, 3H), 3.44 (t, *J* = 6.1 Hz, 2H), 1.87–1.53 (m, 6H), 1.38–1.07 (m, 4H), 1.00 (t, *J* = 11.9 Hz, 1H). ^13^C NMR (100 MHz, DMSO-*d*_6_) δ 166.84, 164.26 (d, *J* = 11.9 Hz), 161.45, 158.80 (d, *J* = 10.9 Hz), 157.98, 153.82, 143.47, 135.58, 132.28 (d, *J* = 11.1 Hz), 129.63 (d, *J* = 25.7 Hz), 126.67, 125.71 (d, *J* = 10.7 Hz), 120.32, 112.22 (d, *J* = 22.5 Hz), 109.85, 106.21 (t, *J* = 26.1 Hz), 53.88, 47.55, 37.33, 31.08, 26.51, 25.90. ^1^H and ^13^C NMR spectra were shown in Figure S23. HRMS (ESI) *m*/*z*: [M – H]^–^ calcd for C**_26_**H**_25_**F**_2_**N**_6_**O**_3_**S, 539.1755; found, 539.1725.

*N*-(5-(4-((cyclopropylmethyl)amino)pyrido[2,3-*d*]pyrimidin-6-yl)-2-methoxypyridin-3-yl)-2,4-difluorobenzenesulfonamide (**17l**): flash column chromatography (hexane: ethyl acetate = 1:1) as a white solid (96 mg, 77%). ^1^H NMR (400 MHz, DMSO-*d*_6_) δ 10.36 (s, 1H), 9.30 (d, *J* = 2.4 Hz, 1H), 9.03 (d, *J* = 2.5 Hz, 1H), 8.77 (t, *J* = 5.5 Hz, 1H), 8.60 (s, 1H), 8.56 (d, *J* = 2.4 Hz, 1H), 8.14 (d, *J* = 2.4 Hz, 1H), 7.75 (td, *J* = 8.5, 6.2 Hz, 1H), 7.57 (ddd, *J* = 11.2, 9.3, 2.5 Hz, 1H), 7.20 (td, *J* = 8.5, 2.5 Hz, 1H), 3.64 (s, 3H), 3.55–3.43 (m, 2H), 1.22 (ddd, *J* = 14.0, 6.6, 2.8 Hz, 1H), 0.51 (dt, *J* = 8.1, 2.9 Hz, 2H), 0.40–0.29 (m, 2H). ^13^C NMR (100 MHz, DMSO-*d*_6_) δ 170.77, 166.79 (d, *J* = 11.7 Hz), 164.27 (d, *J* = 11.8 Hz), 161.27, 161.11, 158.78 (d, *J* = 11.3 Hz), 158.00, 143.42, 135.47, 132.29 (d, *J* = 10.8 Hz), 129.80, 129.54, 126.65, 125.72 (dd, *J* = 14.6, 3.8 Hz), 120.36, 112.21 (dd, *J* = 22.2, 3.7 Hz), 109.80, 106.21 (t, *J* = 26.2 Hz), 67.48, 60.20, 53.88, 45.96, 25.58, 21.21, 14.54, 10.91, 4.06. ^1^H and ^13^C NMR spectra were shown in Figure S24. HRMS (ESI) *m*/*z*: [M + H]^+^ calcd for C_23_H_21_F_2_N_6_O_3_S, 499.1286; found, 499.1369.

General Procedure C was used for the synthesis of **22a–l**.

The synthesis of ethyl 2-(6-bromoquinolin-4-yl)oxazole-5-carboxylate (**20**). To a mixture of zinc chloride (1.0 M in THF, 6 mL,6 mmol) and compound **18** (242 μL, 2 mmol) in THF (10 mL) at −10 °C under argon, lithium hexamethyldisilazide (1.0 M in THF, 3 mL, 3 mmol) was added and the mixture was stirred at −10 °C for 1 h. Then, 6-bromo-4-iodoquinoline (333 mg, 1 mmol) and Pd(PPh_3_)_4_ (116 mg, 0.1 mmol) were added and the mixture was stirred at 60 °C for 15 h. It was cooled to room temperature and neutralized by saturated ammonium chloride (30 mL). Ethyl acetate (10 mL) was added and the mixture was stirred for 15 min. The separated organic layer was evaporated in vacuum and the residue was purified by flash column chromatography to obtain a white solid (**20**) (245mg, 71%). ^1^H NMR (400 MHz, DMSO-*d6*) δ 9.44 (d, J = 2.2 Hz, 1H), 9.10 (d, J = 4.6 Hz, 1H), 8.31 (s, 1H), 8.13 (d, J = 4.5 Hz, 1H), 8.05 (d, J = 8.9 Hz, 1H), 7.98 (dd, J = 9.0, 2.3 Hz, 1H), 4.40 (q, J = 7.1 Hz, 2H), 1.36 (t, J = 7.0 Hz, 3H).

The synthesis of 2-(6-bromoquinolin-4-yl)-*N*-isopropyloxazole-5-carboxamide (**21c**). In a sealed tube, a mixture of ester **20** (88 mg, 0.25 mmol) and isopropyl amine (1 mL) was stirred at 100 °C for 4 h. It was cooled to room temperature and H_2_O (5 mL) was added. The suspension was filtered and dried to give an ashy solid **21c** (76.3 mg, 86%), which was used without purification.

**21a–b, 21d–l** were prepared by a similar procedure described for the synthesis of **21c**.

The synthesis of 2-(6-(5-((2,4-difluorophenyl)sulfonamido)-6-methoxypyridin-3-yl)quinolin-4-yl)-*N*-isopropyloxazole-5-carboxamide (**22c**). Under argon protection, PdCl_2_(dppf)•DCM (21 mg, 0.025 mmol) was added to a mixture of bromo compound **21c** (76.3 mg, 0.21mmol), borate **5** (213 mg, 0.5 mmol), K_2_CO_3_ (52 mg, 0.375 mmol) in 1,4-dioxane (10 mL), and H_2_O (2 mL). The mixture was refluxed for 4 h, then concentrated and purified by flash column chromatography (hexane: ethyl acetate = 1:1) to obtain the coupled **22c** as a white solid (83.9 mg, 70%). ^1^H NMR (400 MHz, DMSO-*d*_6_) δ 10.39 (s, 1H), 9.56 (d, *J* = 2.0 Hz, 1H), 9.13 (d, *J* = 4.6 Hz, 1H), 8.65 (d, *J* = 7.9 Hz, 1H), 8.47 (d, *J* = 2.3 Hz, 1H), 8.39 (d, *J* = 4.6 Hz, 1H), 8.23 (d, *J* = 8.8 Hz, 1H), 8.15 (dd, *J* = 9.0, 2.2 Hz, 1H), 8.13 (s, 1H), 8.03 (d, *J* = 2.3 Hz, 1H), 3.70 (s, 3H), 1.23 (d, *J* = 6.6 Hz, 6H). ^13^C NMR (100 MHz, DMSO-*d*_6_) δ 170.79, 166.83 (d, *J* = 12.0 Hz), 164.30 (d, *J* = 11.6 Hz), 161.13 (d, *J* = 13.5 Hz), 159.62, 158.57 (d, *J* = 13.5 Hz), 158.12, 155.80, 150.91, 148.42, 146.28, 143.02, 136.41, 134.18, 132.37 (d, *J* = 10.7 Hz), 131.82, 131.18, 129.85, 129.55, 129.18, 125.53 (d, *J* = 18.1 Hz), 124.02, 123.53, 121.89, 120.54, 113.19–111.28 (m), 106.32 (t, *J* = 26.1 Hz), 60.21, 53.99, 41.32, 22.72, 21.22, 14.54. ^1^H and ^13^C NMR spectra were shown in Figure S27. HRMS (ESI) *m*/*z*: [M – H]^–^ calcd for C**_28_**H**_22_**F**_2_**N**_5_**O**_5_**S, 578.1388; found, 578.1355.

**22a–b**, **22d−l** were prepared by a similar procedure described for the synthesis of **22c**.

Ethyl-2-(6-(5-((2,4-difluorophenyl)sulfonamido)-6-methoxypyridin-3-yl)quinolin-4-yl)oxazole-5-carboxylate (**22a**): flash column chromatography (hexane: ethyl acetate = 1:1) as a white solid (99 mg, 68%). ^1^H NMR (400 MHz, DMSO-*d*_6_) δ 10.37 (s, 1H), 9.41 (d, *J* = 2.0 Hz, 1H), 9.10 (d, *J* = 4.5 Hz, 1H), 8.46 (d, *J* = 2.3 Hz, 1H), 8.36 (d, *J* = 1.1 Hz, 1H), 8.23 (d, *J* = 8.8 Hz, 1H), 8.20–8.13 (m, 2H), 8.03 (d, *J* = 2.3 Hz, 1H), 7.80 (td, *J* = 8.5, 6.2 Hz, 1H), 7.57 (ddd, *J* = 11.2, 9.3, 2.5 Hz, 1H), 7.20 (td, *J* = 8.5, 2.5 Hz, 1H), 4.42 (q, *J* = 7.1 Hz, 2H), 3.70 (s, 3H), 1.36 (t, *J* = 7.1 Hz, 3H). ^13^C NMR (100 MHz, DMSO-*d*_6_) δ 166.83 (d, *J* = 11.9 Hz), 164.30 (d, *J* = 11.6 Hz), 161.47, 161.13 (d, *J* = 13.9 Hz), 158.57 (d, *J* = 13.6 Hz), 158.24, 157.51, 151.03, 148.42, 143.17, 142.93, 136.51, 135.97, 134.47, 132.36 (d, *J* = 10.8 Hz), 131.26, 130.06–128.52 (m), 125.53 (d, *J* = 14.3 Hz), 123.98, 123.24, 122.05, 120.43, 112.33 (d, *J* = 19.3 Hz), 106.31 (t, *J* = 26.2 Hz), 62.07, 53.98, 22.72, 14.58. ^1^H and ^13^C NMR spectra were shown in Figure S25. HRMS (ESI) *m*/*z*: [M – H]^–^ calcd for C**_27_**H**_19_**F**_2_**N**_4_**O**_6_**S, 565.1072; found, 565.1016.

2-(6-(5-((2,4-difluorophenyl)sulfonamido)-6-methoxypyridin-3-yl)quinolin-4-yl)-*N*-methyloxazole-5-carboxamide (**22b**): flash column chromatography (hexane: ethyl acetate = 1:1) as a white solid (78 mg, 57%). ^1^H NMR (400 MHz, DMSO-*d*_6_) δ 10.40 (s, 1H), 9.56 (d, *J* = 2.1 Hz, 1H), 9.14 (d, *J* = 4.6 Hz, 1H), 8.87 (q, *J* = 4.5 Hz, 1H), 8.47 (d, *J* = 2.4 Hz, 1H), 8.37 (d, *J* = 4.5 Hz, 1H), 8.23 (d, *J* = 8.8 Hz, 1H), 8.16 (dd, *J* = 8.8, 2.1 Hz, 1H), 8.12 (s, 1H), 8.03 (d, *J* = 2.4 Hz, 1H), 7.81 (td, *J* = 8.6, 6.2 Hz, 1H), 7.63–7.53 (m, 1H), 7.20 (td, *J* = 8.3, 2.3 Hz, 1H), 3.70 (s, 3H), 2.85 (d, *J* = 4.6 Hz, 3H). ^13^C NMR (100 MHz, DMSO-*d*_6_) δ 166.83 (d, *J* = 11.8 Hz), 159.57, 158.16, 157.10, 150.94, 148.43, 146.22, 143.09, 136.44, 134.30, 132.36 (d, *J* = 11.1 Hz), 131.63, 131.20, 129.83, 129.56, 129.23, 125.51 (d, *J* = 14.0 Hz), 124.02, 123.50, 121.81, 120.50, 112.34 (d, *J* = 23.7 Hz), 106.32 (t, *J* = 26.0 Hz), 60.22, 54.00. ^1^H and ^13^C NMR spectra were shown in Figure S26. HRMS (ESI) *m*/*z*: [M – H]^–^ calcd for C**_26_**H**_18_**F**_2_**N**_5_**O**_5_**S, 550.1075; found, 550.1032.

*N*-cyclopropyl-2-(6-(5-((2,4-difluorophenyl)sulfonamido)-6-methoxypyridin-3-yl)quinolin-4-yl)oxazole-5-carboxamide (**22d**): flash column chromatography (hexane: ethyl acetate = 1:1) as a white solid (66 mg, 46%). ^1^H NMR (400 MHz, DMSO-*d*_6_) δ 10.38 (s, 1H), 9.55 (d, *J* = 2.0 Hz, 1H), 9.12 (d, *J* = 4.6 Hz, 1H), 8.89 (d, *J* = 4.0 Hz, 1H), 8.47 (d, *J* = 2.3 Hz, 1H), 8.37 (d, *J* = 4.5 Hz, 1H), 8.23 (d, *J* = 8.8 Hz, 1H), 8.15 (dd, *J* = 8.8, 2.1 Hz, 1H), 8.12 (s, 1H), 8.03 (d, *J* = 2.4 Hz, 1H), 7.89–7.78 (m, 1H), 7.57 (ddd, *J* = 11.4, 9.2, 2.5 Hz, 1H), 7.20 (td, *J* = 8.5, 2.5 Hz, 1H), 3.71 (s, 3H), 3.08–2.83 (m, 1H), 0.77 (dt, *J* = 6.9, 3.3 Hz, 2H), 0.69–0.61 (m, 2H). ^13^C NMR (100 MHz, DMSO-*d*_6_) δ 159.68, 158.12, 157.83, 150.92, 148.42, 146.08, 143.01, 136.44, 134.15, 132.37 (d, *J* = 10.8 Hz), 131.79, 131.19, 129.81, 129.54, 129.20, 125.52 (d, *J* = 14.5 Hz), 124.01, 123.49, 121.89, 120.56, 112.35 (d, *J* = 22.5 Hz), 106.31 (t, *J* = 26.1 Hz), 60.22, 54.00, 22.98, 21.22, 14.55, 6.27. ^1^H and ^13^C NMR spectra were shown in Figure S28. HRMS (ESI) *m*/*z*: [M + H]^+^ calcd for C_28_H_22_F_2_N_5_O_5_S, 578.1231; found, 578.1301.

2-(6-(5-((2,4-difluorophenyl)sulfonamido)-6-methoxypyridin-3-yl)quinolin-4-yl)-*N*-(2-methoxyethyl)oxazole-5-carboxamide (**22e**): flash column chromatography (hexane: ethyl acetate = 1:1) as a white solid (106 mg, 71%). ^1^H NMR (400 MHz, DMSO-*d*_6_) δ 10.38 (s, 1H), 9.56 (d, *J* = 2.1 Hz, 1H), 9.13 (d, *J* = 4.6 Hz, 1H), 8.99 (d, *J* = 5.5 Hz, 1H), 8.47 (d, *J* = 2.3 Hz, 1H), 8.39 (d, *J* = 4.6 Hz, 1H), 8.23 (d, *J* = 8.8 Hz, 1H), 8.19–8.11 (m, 2H), 8.03 (d, *J* = 2.4 Hz, 1H), 7.81 (td, *J* = 8.6, 6.2 Hz, 1H), 7.58 (ddd, *J* = 10.5, 9.1, 2.5 Hz, 1H), 7.21 (td, *J* = 8.6, 2.5 Hz, 1H), 3.71 (s, 3H), 3.50 (dt, *J* = 6.2, 2.9 Hz, 4H), 3.29 (s, 3H). ^13^C NMR (100 MHz, DMSO-*d*_6_) δ 170.79, 166.83 (d, *J* = 11.9 Hz), 164.30 (d, *J* = 11.6 Hz), 161.12 (d, *J* = 13.4 Hz), 159.70, 158.56 (d, *J* = 13.4 Hz), 158.13, 156.79, 150.91, 148.42, 146.06, 143.01, 136.43, 134.18, 132.37 (d, *J* = 10.7 Hz), 131.96, 131.19, 129.82, 129.55, 129.20, 126.06–125.07 (m), 124.03, 123.50, 121.88, 120.56, 113.48–110.87 (m), 106.31 (t, *J* = 26.0 Hz), 70.87, 67.48, 60.21, 58.43, 53.99, 22.98, 21.22, 14.55, 6.27. ^1^H and ^13^C NMR spectra were shown in Figure S29. HRMS (ESI) *m*/*z*: [M + H]^+^ calcd for C_28_H_24_F_2_N_5_O_6_S, 596.1337; found, 596.1420.

2-(6-(5-((2,4-difluorophenyl)sulfonamido)-6-methoxypyridin-3-yl)quinolin-4-yl)-*N*-(3-methoxypropyl)oxazole-5-carboxamide (**22f**): flash column chromatography (hexane: ethyl acetate = 1:1) as a white solid (90 mg, 59%). ^1^H NMR (400 MHz, DMSO-*d*_6_) δ 10.38 (s, 1H), 9.55 (d, *J* = 2.0 Hz, 1H), 9.13 (d, *J* = 4.6 Hz, 1H), 8.90 (t, *J* = 5.8 Hz, 1H), 8.47 (d, *J* = 2.3 Hz, 1H), 8.37 (d, *J* = 4.6 Hz, 1H), 8.23 (d, *J* = 8.8 Hz, 1H), 8.16 (dd, *J* = 8.8, 2.1 Hz, 1H), 8.13 (s, 1H), 8.03 (d, *J* = 2.4 Hz, 1H), 7.81 (td, *J* = 8.6, 6.2 Hz, 1H), 7.58 (ddd, *J* = 10.5, 9.2, 2.5 Hz, 1H), 7.25–7.16 (m, 1H), 3.71 (s, 3H), 3.41 (t, *J* = 6.2 Hz, 2H), 3.39–3.34 (m, 2H), 3.25 (s, 3H), 1.81 (p, *J* = 6.6 Hz, 2H). ^13^C NMR (100 MHz, DMSO-*d6*) δ 159.62, 158.14, 156.66, 150.93, 148.43, 146.20, 143.09, 136.43, 134.25, 132.42, 132.32, 131.78, 131.19, 129.86, 129.56, 129.21, 125.46, 124.03, 123.51, 121.85, 120.48, 112.46, 112.25, 106.58, 106.32, 106.06, 70.03, 60.21, 58.40, 54.00, 36.54, 29.67, 14.55. ^1^H and ^13^C NMR spectra were shown in Figure S30. HRMS (ESI) *m*/*z*: [M – H]^–^ calcd for C**_29_**H**_24_**F**_2_**N**_5_**O**_6_**S, 608.1494; found, 608.1464.

*N*-butyl-2-(6-(5-((2,4-difluorophenyl)sulfonamido)-6-methoxypyridin-3-yl)quinolin-4-yl)oxazole-5-carboxamide (**22g**): flash column chromatography (hexane: ethyl acetate = 1:1) as a white solid (95 mg, 64%). ^1^H NMR (400 MHz, DMSO-*d*_6_) δ 10.40 (s, 1H), 9.55 (d, *J* = 2.0 Hz, 1H), 9.13 (d, *J* = 4.6 Hz, 1H), 8.87 (t, *J* = 5.8 Hz, 1H), 8.47 (d, *J* = 2.3 Hz, 1H), 8.37 (d, *J* = 4.5 Hz, 1H), 8.23 (d, *J* = 8.7 Hz, 1H), 8.15 (dd, *J* = 8.8, 2.1 Hz, 1H), 8.12 (s, 1H), 8.03 (d, *J* = 2.3 Hz, 1H), 7.81 (td, *J* = 8.6, 6.2 Hz, 1H), 7.58 (ddd, *J* = 11.2, 9.3, 2.5 Hz, 1H), 7.20 (td, *J* = 8.5, 2.5 Hz, 1H), 3.70 (s, 3H), 3.30 (d, *J* = 7.2 Hz, 2H), 1.64–1.50 (m, 2H), 1.36 (h, *J* = 7.4 Hz, 2H), 0.92 (t, *J* = 7.3 Hz, 3H). ^13^C NMR (100 MHz, DMSO-*d*_6_) δ 170.80, 166.82 (d, *J* = 11.7 Hz), 164.30 (d, *J* = 11.6 Hz), 161.12 (d, *J* = 13.4 Hz), 159.60, 158.56 (d, *J* = 13.5 Hz), 158.13, 156.57, 150.92, 148.41, 146.23, 143.03, 136.42, 134.22, 132.37 (d, *J* = 11.0 Hz), 131.73, 131.18, 129.84, 129.55, 129.20, 125.52 (dd, *J* = 14.5, 3.7 Hz), 124.02, 123.51, 121.85, 120.54, 112.33 (dd, *J* = 22.2, 3.5 Hz), 106.32 (t, *J* = 26.2 Hz), 60.22, 53.99, 38.81, 31.67, 23.07, 21.22, 20.08, 14.54, 14.14. ^1^H and ^13^C NMR spectra were shown in Figure S31. HRMS (ESI) *m*/*z*: [M – H]^–^ calcd for C**_29_**H**_24_**F**_2_**N**_5_**O**_5_**S, 592.1544; found, 592.1503.

*N*-(cyclopropylmethyl)-2-(6-(5-((2,4-difluorophenyl)sulfonamido)-6-methoxypyridin-3-yl)quinolin-4-yl)oxazole-5-carboxamide (**22h**): flash column chromatography (hexane: ethyl acetate = 1:1) as a white solid (77 mg, 52%). ^1^H NMR (400 MHz, DMSO-*d*_6_) δ 10.41–10.33 (m, 1H), 9.56 (s, 1H), 9.14 (d, *J* = 4.6 Hz, 1H), 9.00 (t, *J* = 5.7 Hz, 1H), 8.46 (s, 1H), 8.39 (d, *J* = 4.5 Hz, 1H), 8.24 (d, *J* = 8.7 Hz, 1H), 8.16 (d, *J* = 10.0 Hz, 2H), 8.03 (s, 1H), 7.82 (q, *J* = 7.9 Hz, 1H), 7.56 (t, *J* = 10.1 Hz, 1H), 7.20 (t, *J* = 8.0 Hz, 1H), 3.71 (s, 3H), 3.21 (t, *J* = 6.4 Hz, 2H), 1.07 (d, *J* = 8.1 Hz, 1H), 0.48 (d, *J* = 7.7 Hz, 3H), 0.28 (d, *J* = 5.0 Hz, 2H). ^13^C NMR (151 MHz, DMSO-*d*_6_) δ 169.35, 166.35 (d, *J* = 12.3 Hz), 164.66 (d, *J* = 12.0 Hz), 160.69 (d, *J* = 13.5 Hz), 159.66, 158.98 (d, *J* = 13.5 Hz), 158.14, 156.62, 150.93, 148.42, 146.22, 142.78, 136.50, 133.98, 132.35 (d, *J* = 10.9 Hz), 131.88, 131.19, 129.86, 129.54, 129.24, 125.66, 124.04, 123.51, 121.89, 120.82, 112.30 (d, *J* = 22.1 Hz), 106.30 (t, *J* = 26.2 Hz), 53.98, 43.57, 11.44, 11.30, 3.93, 3.66. ^1^H and ^13^C NMR spectra were shown in Figure S32. HRMS (ESI) *m*/*z*: [M + H]^+^ calcd for C_29_H_24_F_2_N_5_O_5_S, 592.1388; found, 592.1471.

*N*-(cyclohexylmethyl)-2-(6-(5-((2,4-difluorophenyl)sulfonamido)-6-methoxypyridin-3-yl)quinolin-4-yl)oxazole-5-carboxamide (**22i**): flash column chromatography (hexane: ethyl acetate = 1:1) as a white solid (73 mg, 46%). ^1^H NMR (400 MHz, DMSO-*d*_6_) δ 10.39 (s, 1H), 9.55 (d, *J* = 2.1 Hz, 1H), 9.13 (d, *J* = 4.5 Hz, 1H), 8.87 (t, *J* = 6.0 Hz, 1H), 8.47 (d, *J* = 2.3 Hz, 1H), 8.38 (d, *J* = 4.6 Hz, 1H), 8.23 (d, *J* = 8.8 Hz, 1H), 8.15 (dd, *J* = 8.9, 2.1 Hz, 1H), 8.13 (s, 1H), 8.03 (d, *J* = 2.4 Hz, 1H), 7.81 (td, *J* = 8.5, 6.2 Hz, 1H), 7.58 (ddd, *J* = 11.3, 9.3, 2.5 Hz, 1H), 7.20 (td, *J* = 8.5, 2.5 Hz, 1H), 3.71 (s, 3H), 3.18 (s, 2H), 1.74 (d, *J* = 14.6 Hz, 2H), 1.71–1.66 (m, 2H), 1.59 (ddd, *J* = 18.4, 9.5, 5.8 Hz, 2H), 1.30–1.05 (m, 4H), 0.96–0.90 (m, 1H). ^13^C NMR (100 MHz, DMSO-*d*_6_) δ 166.84 (d, *J* = 11.8 Hz), 164.31 (d, *J* = 11.8 Hz), 161.12 (d, *J* = 13.7 Hz), 159.62, 158.56 (d, *J* = 13.4 Hz), 158.12, 156.68, 150.93, 148.42, 146.25, 143.09, 136.41, 134.26, 132.38 (d, *J* = 11.0 Hz), 131.75, 131.19, 129.87, 129.56, 129.21, 125.50 (d, *J* = 10.6 Hz), 124.03, 123.52, 121.88, 120.46, 112.35 (d, *J* = 21.0 Hz), 106.32 (t, *J* = 26.0 Hz), 60.21, 53.99, 45.32, 38.01, 30.95, 26.48, 25.86, 21.22, 14.55. ^1^H and ^13^C NMR spectra were shown in Figure S33. HRMS (ESI) *m*/*z*: [M – H]^–^ calcd for C**_32_**H**_28_**F**_2_**N**_5_**O**_5_**S, 632.1857; found, 632.1823.

*N*-cyclohexyl-2-(6-(5-((2,4-difluorophenyl)sulfonamido)-6-methoxypyridin-3-yl)quinolin-4-yl)oxazole-5-carboxamide (**22j**): flash column chromatography (hexane: ethyl acetate = 1:1) as a white solid (76 mg, 49%). ^1^H NMR (400 MHz, DMSO-*d*_6_) δ 10.40–10.36 (m, 1H), 9.55 (d, *J* = 2.1 Hz, 1H), 9.13 (d, *J* = 4.5 Hz, 1H), 8.63 (d, *J* = 8.1 Hz, 1H), 8.48 (d, *J* = 2.4 Hz, 1H), 8.40 (d, *J* = 4.6 Hz, 1H), 8.23 (d, *J* = 8.8 Hz, 2H), 8.18–8.14 (m, 1H), 8.14 (s, 1H), 8.03 (d, *J* = 2.4 Hz, 1H), 7.81 (td, *J* = 8.6, 6.3 Hz, 1H), 7.62–7.52 (m, 1H), 7.20 (td, *J* = 8.5, 2.5 Hz, 1H), 3.84–3.78 (m, 1H), 3.71 (s, 3H), 1.92–1.73 (m, 4H), 1.68–1.40 (m, 2H), 1.38–1.26 (m, 4H). ^13^C NMR (100 MHz, DMSO-*d*_6_) δ 161.19, 159.65, 158.56 (d, *J* = 13.5 Hz), 158.12, 155.75, 150.92, 148.43, 146.30, 143.00, 136.42, 134.15, 132.37 (d, *J* = 10.8 Hz), 131.86, 131.19, 129.88, 129.55, 129.20, 124.03, 123.54, 121.93, 120.59, 112.33 (d, *J* = 24.6 Hz), 106.31 (t, *J* = 26.2 Hz), 60.22, 53.98, 48.58, 32.86, 25.67, 25.38, 21.22, 14.55. ^1^H and ^13^C NMR spectra were shown in Figure S34. HRMS (ESI) *m*/*z*: [M – H]^–^ calcd for C**_31_**H**_26_**F**_2_**N**_5_**O**_5_**S, 618.1701; found, 618.1664.

2-(6-(5-((2,4-difluorophenyl)sulfonamido)-6-methoxypyridin-3-yl)quinolin-4-yl)-*N*-(4-methoxyphenethyl)oxazole-5-carboxamide (**22k**): flash column chromatography (hexane: ethyl acetate = 1:1) as a white solid (92 mg, 55%). ^1^H NMR (400 MHz, DMSO-*d*_6_) δ 10.39 (s, 1H), 9.56 (d, *J* = 2.1 Hz, 1H), 9.14 (d, *J* = 4.6 Hz, 1H), 8.99 (t, *J* = 5.8 Hz, 1H), 8.47 (d, *J* = 2.3 Hz, 1H), 8.37 (d, *J* = 4.6 Hz, 1H), 8.24 (d, *J* = 8.8 Hz, 1H), 8.17 (dd, *J* = 8.8, 2.1 Hz, 1H), 8.13 (s, 1H), 8.03 (d, *J* = 2.3 Hz, 1H), 7.81 (td, *J* = 8.6, 6.3 Hz, 1H), 7.62–7.52 (m, 1H), 7.25–7.08 (m, 3H), 6.90–6.81 (m, 2H), 3.71 (d, *J* = 4.7 Hz, 6H), 3.51 (dt, *J* = 8.1, 6.2 Hz, 2H), 2.83 (t, *J* = 7.5 Hz, 2H). ^13^C NMR (100 MHz, DMSO-*d*_6_) δ 159.67, 158.25, 158.14, 156.60, 150.94, 148.44, 146.19, 136.49, 132.36 (d, *J* = 11.1 Hz), 131.81, 131.49, 131.21, 130.09 129.86, 129.55, 129.25, 124.04, 123.50, 121.85, 114.32, 55.46, 53.99, 41.04, 34.69. ^1^H and ^13^C NMR spectra were shown in Figure S35. HRMS (ESI) *m*/*z*: [M + H]^+^ calcd for C_34_H_28_F_2_N_5_O_6_S, 672.1650; found, 672.1731.

*N*-(4-chlorophenethyl)-2-(6-(5-((2,4-difluorophenyl)sulfonamido)-6-methoxypyridin-3-yl)quinolin-4-yl)oxazole-5-carboxamide (**22l**): flash column chromatography (hexane: ethyl acetate = 1:1) as a white solid (103 mg, 61%). ^1^H NMR (400 MHz, DMSO-*d*_6_) δ 10.39 (s, 1H), 9.55 (d, *J* = 2.2 Hz, 1H), 9.14 (d, *J* = 4.6 Hz, 1H), 9.00 (t, *J* = 5.7 Hz, 1H), 8.47 (d, *J* = 2.4 Hz, 1H), 8.35 (d, *J* = 4.5 Hz, 1H), 8.24 (d, *J* = 8.8 Hz, 1H), 8.16 (dd, *J* = 8.8, 2.1 Hz, 1H), 8.13 (s, 1H), 8.03 (d, *J* = 2.3 Hz, 1H), 7.81 (td, *J* = 8.6, 6.2 Hz, 1H), 7.57 (ddd, *J* = 11.3, 9.3, 2.5 Hz, 1H), 7.39–7.27 (m, 4H), 7.20 (td, *J* = 8.3, 2.3 Hz, 1H), 3.70 (s, 3H), 3.59–3.50 (m, 2H), 2.89 (t, *J* = 7.3 Hz, 2H). ^13^C NMR (100 MHz, DMSO-*d*_6_) δ 159.69, 158.15, 156.65, 150.94, 148.43, 146.11, 138.71, 136.49, 132.36 (d, *J* = 11.2 Hz), 131.88, 131.34, 131.20, 131.07, 129.85, 129.54, 129.25, 128.78, 124.03, 123.49, 121.85, 106.43 (d, *J* = 26.2 Hz), 53.99, 34.78. ^1^H and ^13^C NMR spectra were shown in Figure S36. HRMS (ESI) *m*/*z*: [M + H]^+^ calcd for C_33_H_25_ClF_2_N_5_O_5_S, 676.1155; found, 676.1230.

### 5.2. Enzymatic Inhibitory Activity Assay

The enzymatic inhibitory assays of all target compounds against PI3Kα were performed with the ADP-Glo assay (Promega, Madison, WI, USA, catalog No. V9102/3). Briefly, compounds were continuously diluted to a certain concentration. To the kinase buffer, the compound solution, PI3K enzyme (SignalChem, Richmond, BC, Canada, catalog No. P27-122DH), PIP2 substrate, and ATP were diluted. The mixture was added to a 96-well plate and incubated in a dark place at ambient temperature for 40 min. Then, the kinase reaction was stopped by adding of ADP-Glo reagent and the plate was incubated again at ambient temperature for another 40 min. The kinase detection reagent was added and the signal was collected via PerkinElmer Envision plate reader (PE, Boston, MA, USA).

The inhibitory activity of **22c** on mTOR was determined by LANCE Ultra assay. In short, to a solution of kinase buffer, compound solution, ULight-4E-BP1 (Thr37/46) peptide substrate (PE, Catalog No. TRF0128-M), ATP, and mTOR protein (Millipore, Burlington, MA, USA, Catalog No. 14-770) were added. It was transferred to a 384-well plate and incubated at room temperature for 30 min. The LANCE detection buffer was added, which contained EDTA and Eu-antiphos-4E-BP1 (Thr37/46) antibody (PE, catalog number TRF0216-M). The plate was balanced at ambient temperature for 1 h, and the signal was detected via PerkinElmer Envision plate reader.

### 5.3. Cell Viability Assay

MCF-7 and HCT-116 cells were purchased from the Meilunbio Biotechnology Co., Ltd. (Dalian, China). Cells were cultured at 37 °C with 5% CO_2_, using DMEM or RPMI 1640 medium (Meilunbio, Dalian, China) with 10% (*v*/*v*) fetal bovine serum (Gibco, San Diego, CA, USA) and 1% (*v*/*v*) penicillin and streptomycin (Meilunbio, Dalian, China). CCK-8 assay was used to determine the inhibitory effect in vitro. In short, cells were incubated into 96-well plates at the rate of 5000 cells per well. After 24 h of incubation, 100 μL medium was added, which contained a specific concentration of the test compound. After 72 h, 10 μL CCK-8 regent was added and the plate was incubated at 37 °C, 5% CO_2_ for 1 h. The optical density of each hole at 450 nm was read by a BioTek TS-800 microplate reader. GraphPad Prism 9.3.1 software was used to calculate the IC_50_ value.

### 5.4. Cell Apoptosis and Cycle Analysis

Hoechst 33342/PI staining was used to detect cell survival and apoptosis, and flow cytometry (FACS Verse, BD, Piscataway, NJ, USA) was used to measure cell cycle and survival. Briefly, HCT-116 cells were seeded in 6-well plates and cultured with **22c** at different concentrations for 24 h. As for the Hoechst 33342/PI staining assay, staining buffer with Hoechst 33342/PI was added into each plate and incubated at 4 °C for 30 min. After washing with PBS, the stained cells were observed by fluorescence microscope (IX73, Olympus, Tokyo, Japan). As for detection of cell apoptosis by flow cytometry, cells were stained with Annexin V-FITC/PI. As for cell cycle assay, the cells were fixed with 70% ethanol at 4 °C overnight, and stained with PI/RNase solution for 30 min at 37 °C. All assays were measured by flow cytometry and analyzed by Flowjo 7.6.

### 5.5. Western Blot Analysis

HCT-116 cells were cultured in 100 mm culture dish to 70~80%, and treated with compound **22c** at a specified concentration for 24 h. Then, the cells were scraped off with cell scraper, collected into a precooled 1.5 mL centrifuge tube, and lysed with RIPA buffer (Cwbio, Taizhou, China) containing PMSF (1 mM), NaF (1 mM), Na_3_VO_4_ (1 mM). The cells were lysed at 4 °C for 30 min, and the concentration of protein was determined by the BCA assay kit (Meilunbio, Dalian, China). Then the protein samples (40 μg) were used to conduct SDS-PAGE electrophoresis (Electrophoresis equipment: BIO-RAD, Hercules, CA, USA; Prefabricated-gel: ACE, Shanghai, China). After electrophoresis, the protein was transferred to PVDF membranes (0.45 μM, Millipore, USA). It was sealed with 5% non-fat milk for 1.5 h, washed with TBST for 4 times, and incubated with primary antibody at 4 °C overnight. Then, membranes were recovered to room temperature, washed with TBST for 4 times and incubated with secondary antibody (Huabio, Hangzhou, China) at room temperature for 1.5 h. After incubating, the membranes were washed with TBST for 4 times, visualized with an Amersham Imager 680 (GE, Windsor, CT, USA) through the enhanced ECL detection kit (Meilunbio, Dalian, China). The primary antibodies were anti AKT (1/1000 dilution, CST No. 4691T), anti p-AKT (S473) (1/1000 diluent, CST No. 4060T), anti p-AKT (T308) (1/1000 diluent, CST No. 13038T), and anti GAPDH (1/5000, Huabio, Hangzhou, China).

### 5.6. Molecular Docking

AutoDock 4.2 software was used to perform the molecular docking research. The crystal structures of PI3Kα (PDB code 4JPS) and mTOR kinase (PDB code 4JT6) were gained from the Protein Data Bank. AutoDockTools module was used to prepare the ligands and receptors following these steps. For receptors, all the water molecules were removed except for the water molecule that connects ligands and amino acid residues. For ligands, the 3D structure was created to generate a low-energy structure. The binding modes were analyzed with Pymol.

## 6. Conclusions

Based on a scaffold hopping strategy, 36 sulfonamide methoxypyridine derivatives were synthesized with three types of skeletons: benzo[4,5]thiopheno[3,2-*d*]pyrimidine, pyridine[2,3-*d*]pyrimidine, or quinoline. **22a–l** with quinoline showed ideal kinase inhibitory activity, while the first two types, **11a–l** or **17a–l**, showed poor enzymic or cellular inhibitory activity. Among them, **22c** performed sufficient inhibitory activity both on PI3Kα and mTOR (for PI3Kα, IC_50_ = 0.22 nM, for mTOR, IC_50_ = 23 nM). **22c** could also effectively inhibit the proliferation of MCF-7 cells (IC_50_ = 130 nM) and HCT-116 cells (IC_50_ = 20 nM). Further study showed **22c** could block the cell cycle of HCT-116 cells in G0/G1 phase and prominently induce apoptosis. Therefore, as a novel potent PI3K/mTOR dual inhibitor, **22c** could be used for further exploration of the area.

## Figures and Tables

**Table 1:** Enzyme inhibition of PI3Kα and antiproliferative activity against MCF-7 and HCT-116 cells by **11a–l**.

Compd.	XR	PI3Kα Inhibition (%) at 1 nM	IC_50_(μM) ^a^ MCF-7	IC_50_(μM) ^a^ HCT-116	ClogP ^b^
**11a**	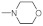	10	0.96 ± 0.23	>1	5.37
**11b**	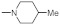	10	>1	>1	7.27
**11c**	-NH(CH_2_)_2_OH	29	0.85 ± 0.37	0.59 ± 0.18	5.22
**11d**	-NHCH(CH_2_)_2_	40	0.51 ± 0.11	0.19 ± 0.12	6.57
**11e**	-NH(CH_2_)_3_OMe	35	>1	>1	6.34
**11f**	-NH(CH_2_)_2_NH_2_	10	0.69 ± 0.25	0.77 ± 0.21	5.30
**11g**	-NHCHMe_2_	44	0.53 ± 0.16	0.22 ± 0.08	6.83
**11h**	-NH(CH_2_)_2_NMe_2_	12	0.70 ± 0.21	0.56 ± 0.10	6.20
**11i**	-NHBu^n^	15	>1	>1	7.58
**11j**	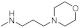	10	>1	>1	6.48
**11k**	-O(CH_2_)_2_NMe_2_	13	0.69 ± 0.11	0.25 ± 0.08	6.19
**11l**	-O(CH_2_)_2_OMe	18	0.86 ± 0.12	>1	5.99
**HS-173**		52	1.76 ± 0.44	0.19 ± 0.05	3.46
**Omipalisib**		89	>4	0.02 ± 0.01	4.07

^a^ IC_50_ values represented as the mean (*n* = 3), ^b^ ClogP values were predicted by SwissADME.

**Table 2:** Enzyme inhibition of PI3Kα and antiproliferative activity against MCF-7 and HCT-116 cells by **17a–l**.

Compd.	XR	PI3Kα Inhibition (%) at 1 nM	IC_50_(μM) ^a^ MCF-7	IC_50_(μM) ^a^ HCT-116	ClogP ^b^
**17a**	-NHCHMe_2_	39	0.85 ± 0.30	0.59 ± 0.15	5.21
**17b**	-NHCH(CH_2_)_2_	21	0.91 ± 0.39	0.35 ± 0.17	4.07
**17c**	-NHBu*^n^*	21	>1	0.83 ± 0.25	4.90
**17d**	-NHC_2_H_4_NMe_2_	31	0.73 ± 0.19	0.52 ± 0.23	4.65
**17e**	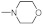	36	0.92 ± 0.41	0.64 ± 0.20	4.27
**17f**	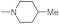	11	0.86 ± 0.30	0.51 ± 0.25	4.58
**17g**	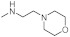	11	>1	>1	5.65
**17h**	-NHC_2_H_4_OH	24	0.93 ± 0.48	0.69 ± 0.12	5.04
**17i**	-NHC_3_H_6_OMe		>1	>1	6.71
**17j**	-NHC_2_H_4_OMe	24	0.75 ± 0.25	0.42 ± 0.12	6.10
**17k**	-NHCH_2_C_6_H_12_	22	>1	>1	6.08
**17l**	-NHCH_2_CH(CH_2_)_2_	25	0.63 ± 0.12	0.47 ± 0.17	6.88
**HS-173**		52	1.76 ± 0.44	0.19 ± 0.05	3.46
**Omipalisib**		89	>4	0.02 ± 0.01	4.07

^a^ IC_50_ values represented as the mean (n = 3), ^b^ ClogP values were predicted by SwissADME.

**Table 3:** Enzyme inhibition of PI3Kα and antiproliferative activity against MCF-7 and HCT-116 cells by **22a–l**.

Compd.	XR	PI3Kα Inhibition (%) at 1 nM	IC_50_ (nM) ^a^ PI3Kα	IC_50_(μM) ^b^ MCF-7	IC_50_(μM) ^b^ HCT-116	ClogP ^c^
**22a**	-OEt	20	-	>1	>1	5.21
**22b**	-NHCH_3_	74	-	0.39 ± 0.08	0.15 ± 0.04	4.07
**22c**	-NHMe_2_	82	0.22	0.13 ± 0.03	0.02 ± 0.01	4.90
**22d**	-NHCH(CH_2_)_2_	82	0.22	0.46 ± 0.11	0.10 ± 0.06	4.65
**22e**	-NH(CH_2_)_2_OMe	69	-	0.20 ± 0.09	0.13 ± 0.05	4.27
**22f**	-NH(CH_2_)_3_OMe	78	0.38	0.29 ± 0.10	0.06 ± 0.02	4.58
**22g**	-NHBu^n^	55	-	0.45 ± 0.09	0.32 ± 0.09	5.65
**22h**	-NHCH_2_CH(CH_2_)_2_	78	0.37	0.35 ± 0.11	0.06 ± 0.01	5.04
**22i**	-NHCH_2_C_6_H_12_	33	-	1.00 ± 0.30	0.77 ± 0.19	6.71
**22j**	-NHC_6_H_12_	18	-	>1	>1	6.10
**22k**	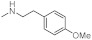	22	-	>1	>1	6.08
**22l**	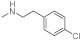	15	–	>1	>1	6.88
**HS-173**		52	1.1	1.76 ± 0.44	0.19 ± 0.05	3.46
**Omipalisib**		89	0.20	>4	0.02 ± 0.01	4.07

^a^ IC_50_ values were represented as the mean (n = 2), and ^b^ IC50 values were represented as the mean (n = 3); ^c^ ClogP values were predicted by SwissADME.

**Table 4:** Inhibition of **22c** against mTOR.

Compd.	IC_50_ (nM) for mTOR ^a^
**22c**	23
**HS-173**	110
**Omipalisib**	35

^a^ IC_50_ values were represented as the mean (n = 2).

## Data Availability

Data are available within article and Supplementary Materials.

## Supplementary Materials

The following supporting information can be downloaded at https://www.mdpi.com/article/10.3390/ph16030461/s1, Figures S1–S36: ^1^H and ^13^C NMR spectra of **11a–l**, **17a–l**, **22a–l**.
