# Supplementary material for: Design, Synthesis, and Biological Evaluation of Sulfonamide Methoxypyridine Derivatives as Novel PI3K/mTOR Dual Inhibitors
Source: Pharmaceuticals (Basel). 2023 Mar 20;16(3):461. doi: 10.3390/ph16030461 (PMC10054477; doi:10.3390/ph16030461)
Supplement: Supplementary file 1 [file pharmaceuticals-16-00461-s001.zip › pharmaceuticals-2266496-supplementary.pdf]

Article

Supplementary Materials

Design, Synthesis, and Biological Evaluation of Pyridinesulfonamide Derivatives as Novel PI3K/mTOR Dual Inhibitors

Haotian Gao [1], Zaolin Li [1], Yuhan Zhang [1], Tong Wang [1], Kai Wang [2], Fang Wang [2,*] and Youjun Xu [1,*]

1 Key Laboratory of Structure-Based Drug Design and Discovery (Ministry of Education), School of Pharmaceutical Engineering, Shenyang Pharmaceutical University, Shenyang, 110016, China
2 Faculty of functional food and wine, Shenyang Pharmaceutical University, Shenyang 110016, China
* Correspondence: e-mail@e-mail.com: (xuyoujun@syphu.edu.cn (Y. Xu); Wangf@syphu.edu.cn (F. Wang))

NMR spectra for final compounds

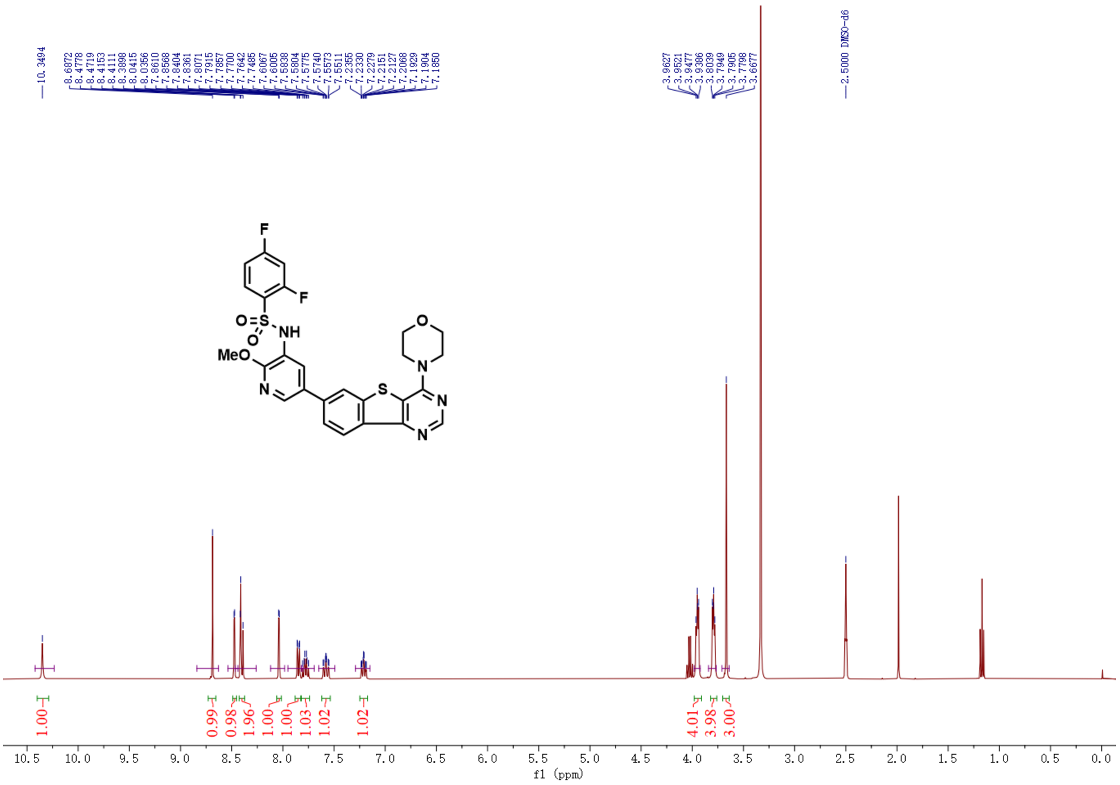

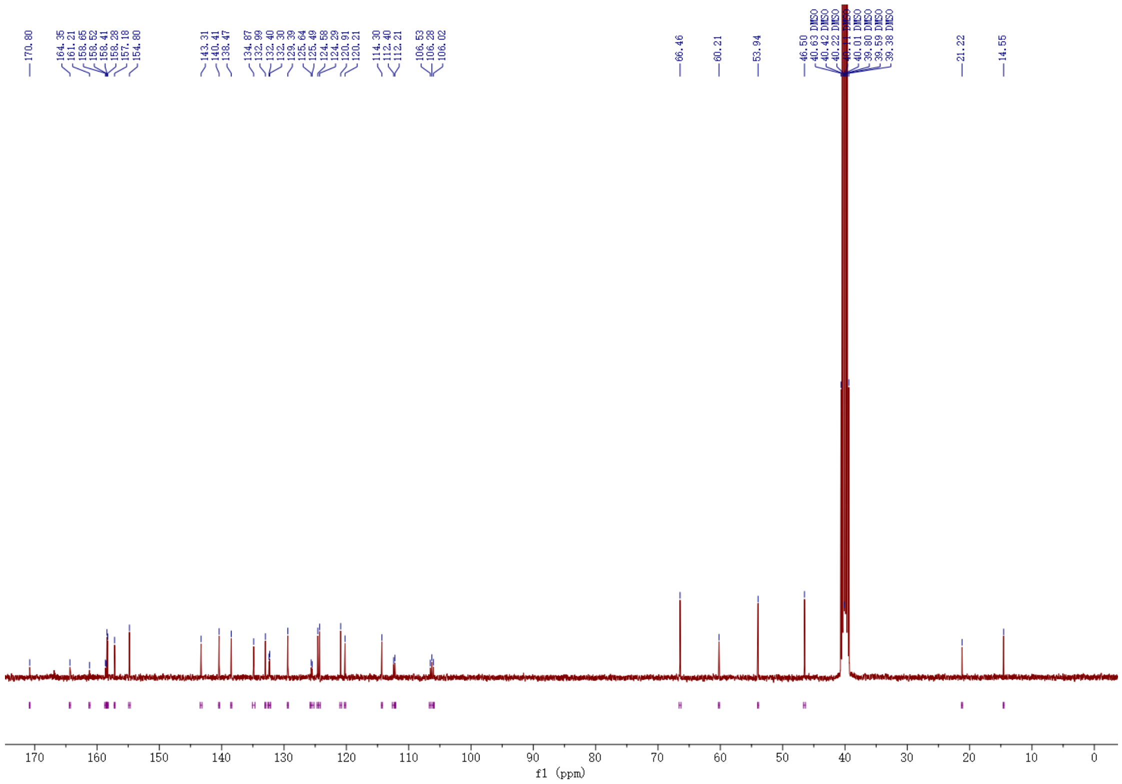

Figure S1. ^{1}H and ^{13}C NMR spectra of **11a** (DMSO-d6).

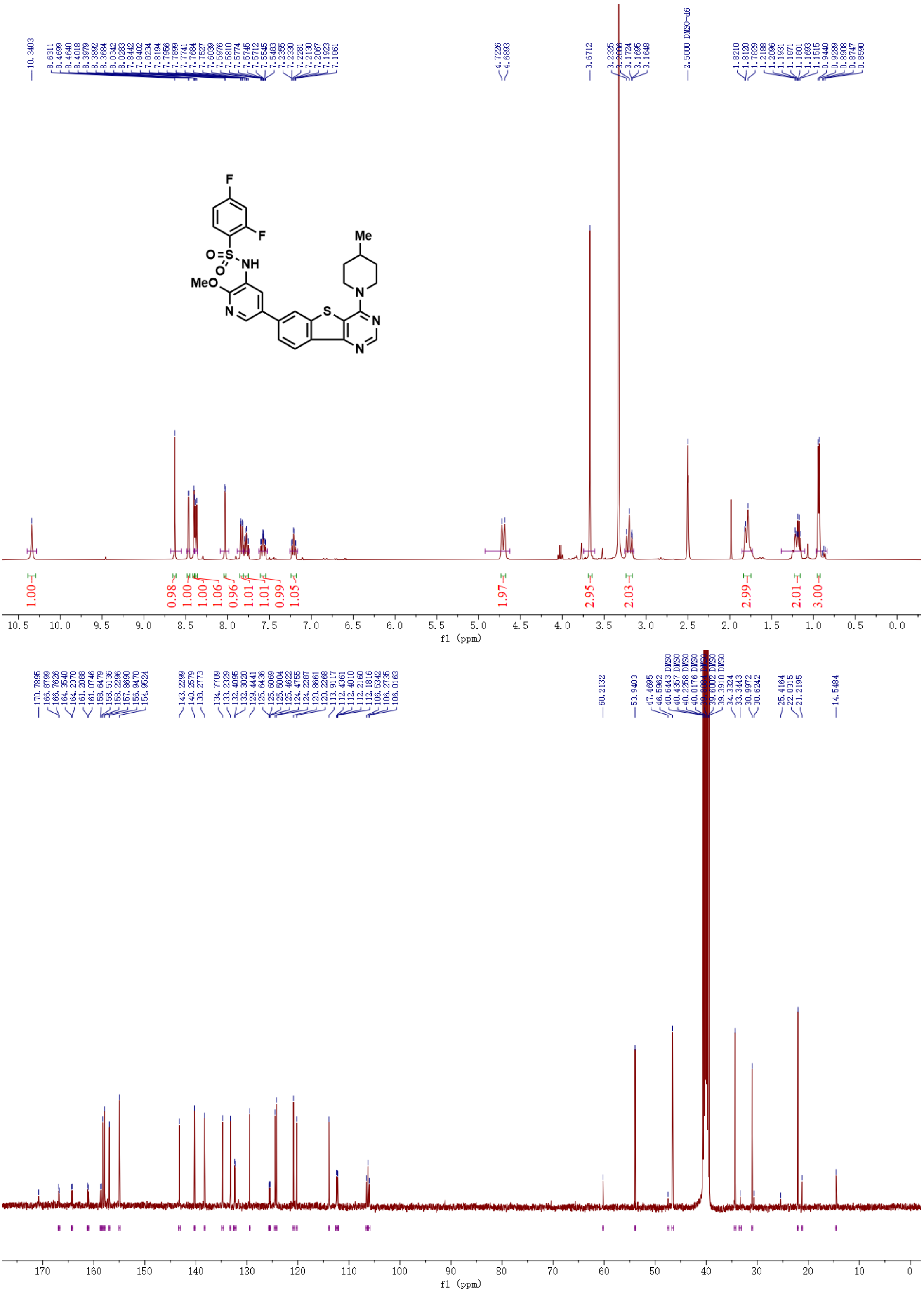

Figure S2. ^{1}H and ^{13}C NMR spectra of **11b** (DMSO-d6).

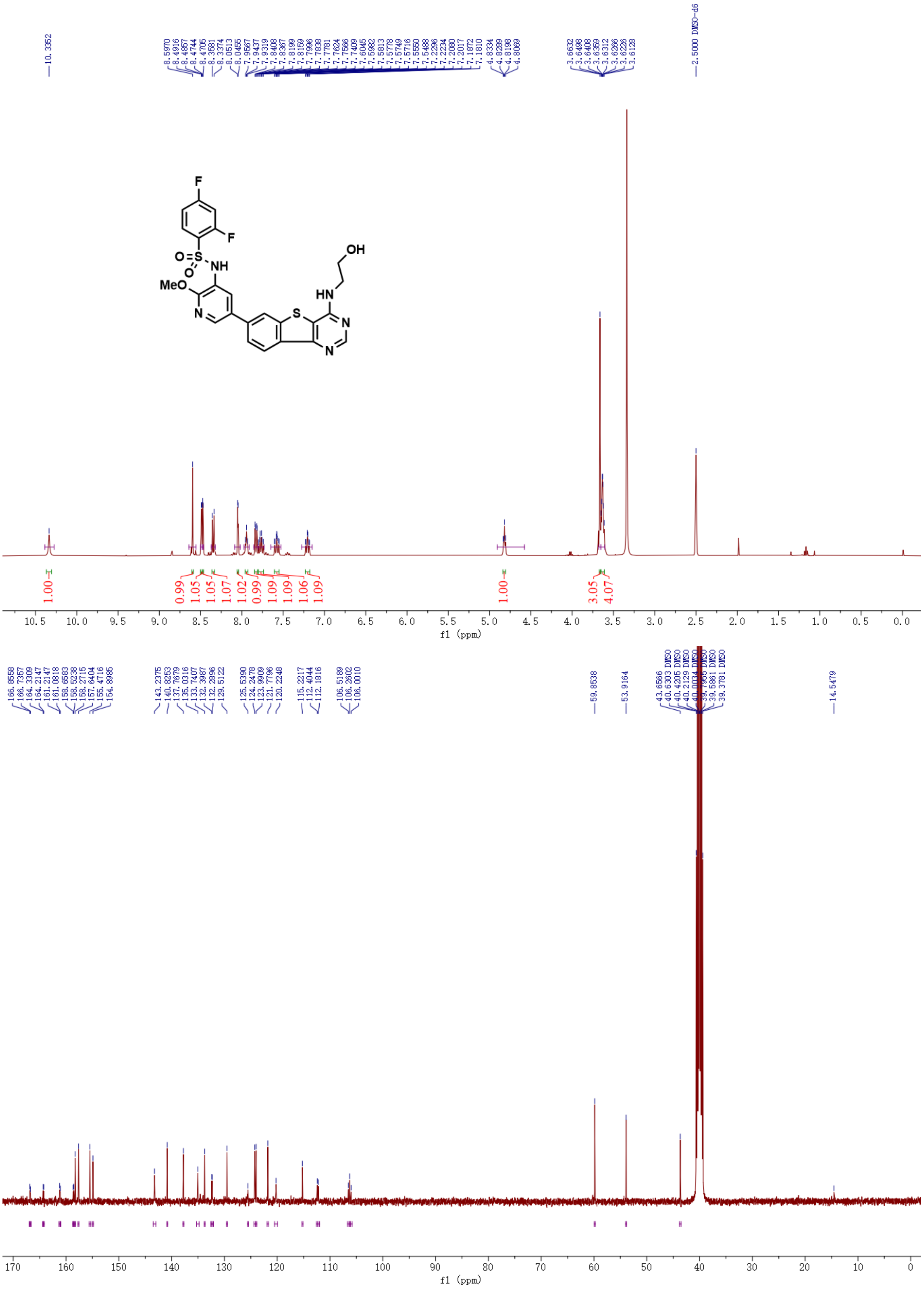

Figure S3. ^{1}H and ^{13}C NMR spectra of **11c** (DMSO-d6).

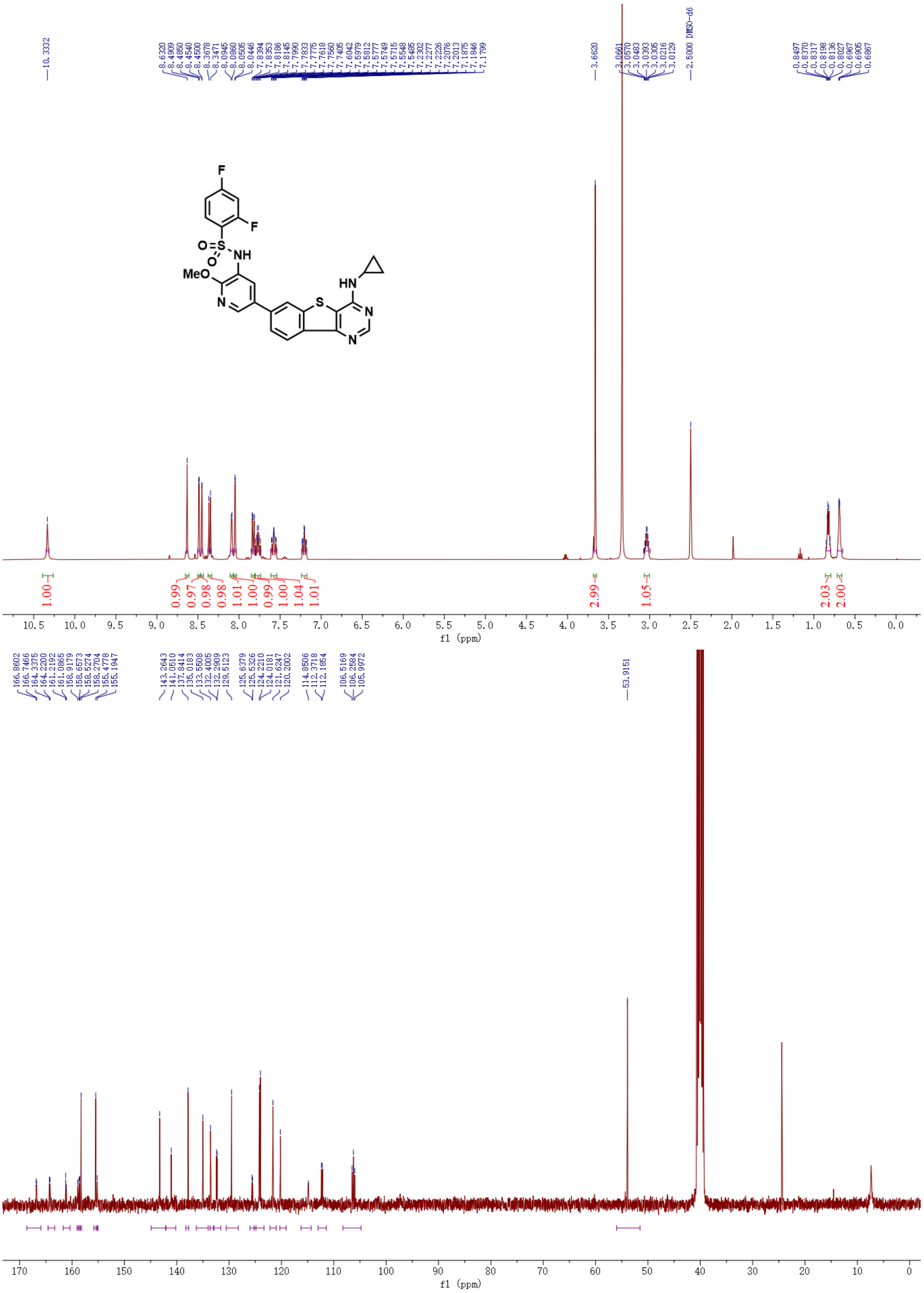

Figure S4. ^{1}H and ^{13}C NMR spectra of **11d** (DMSO-d6).

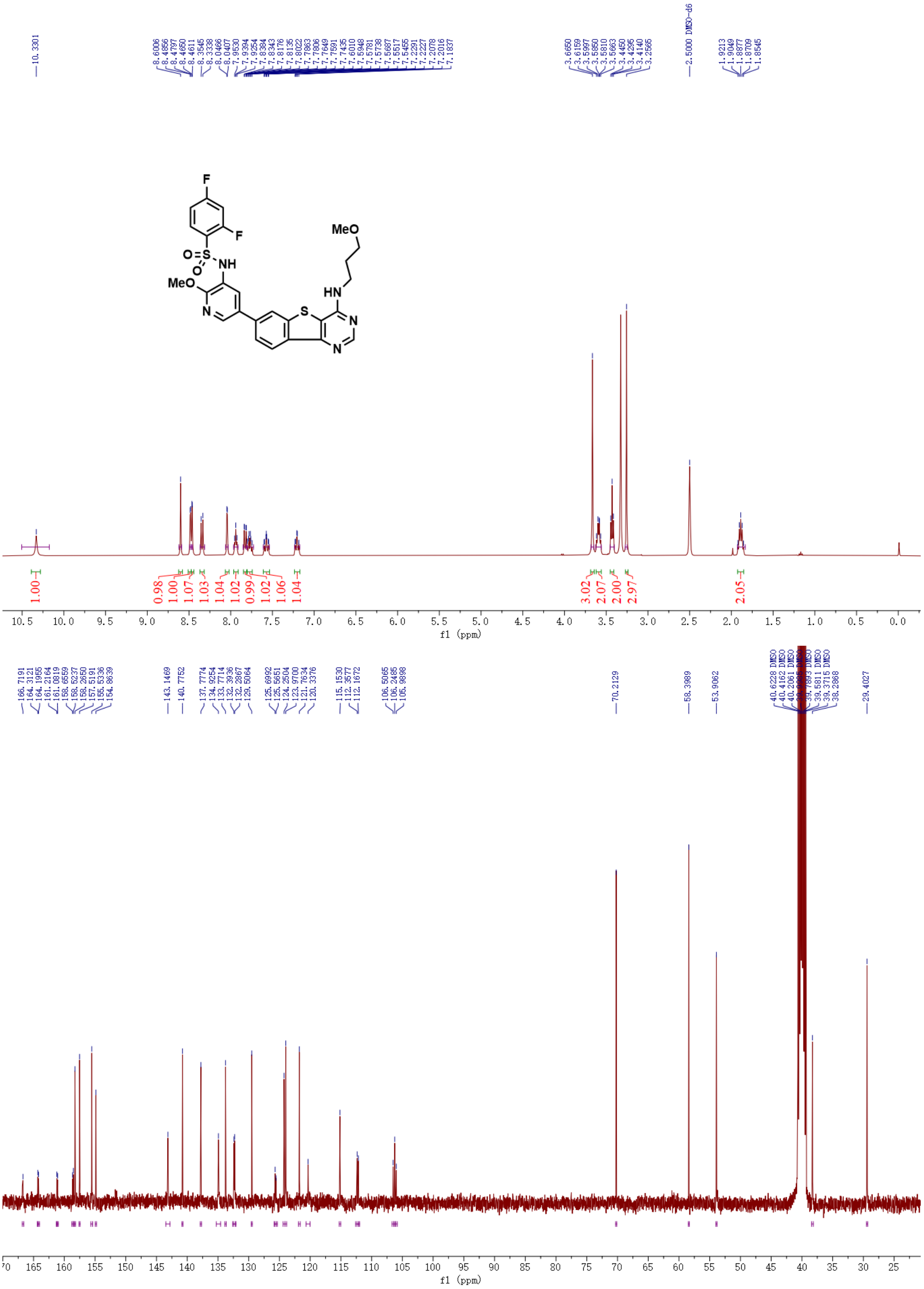

Figure S5. ^{1}H and ^{13}C NMR spectra of **11e** (DMSO-d6).

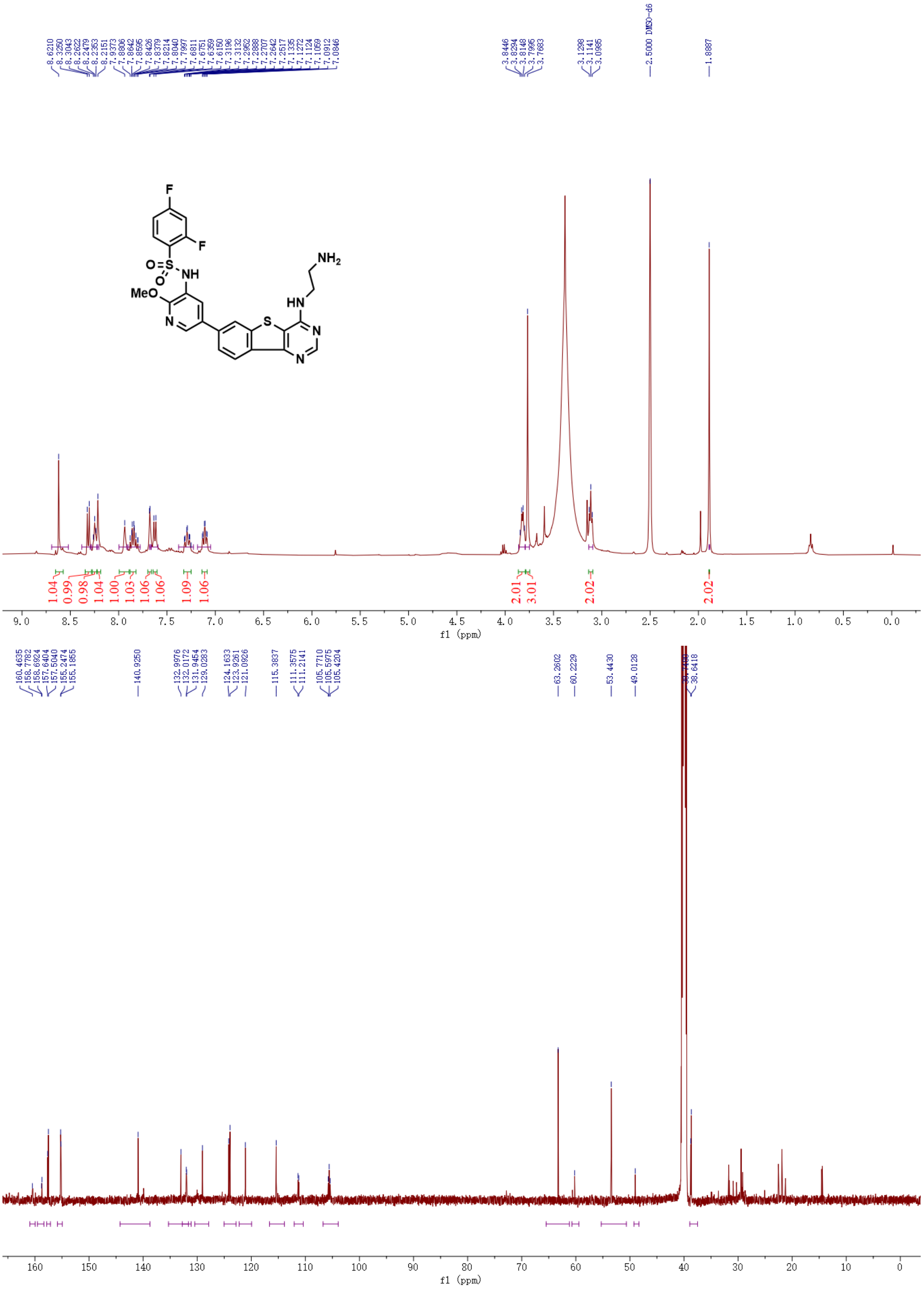

Figure S6. ^{1}H and ^{13}C NMR spectra of **11f** (DMSO-d6).

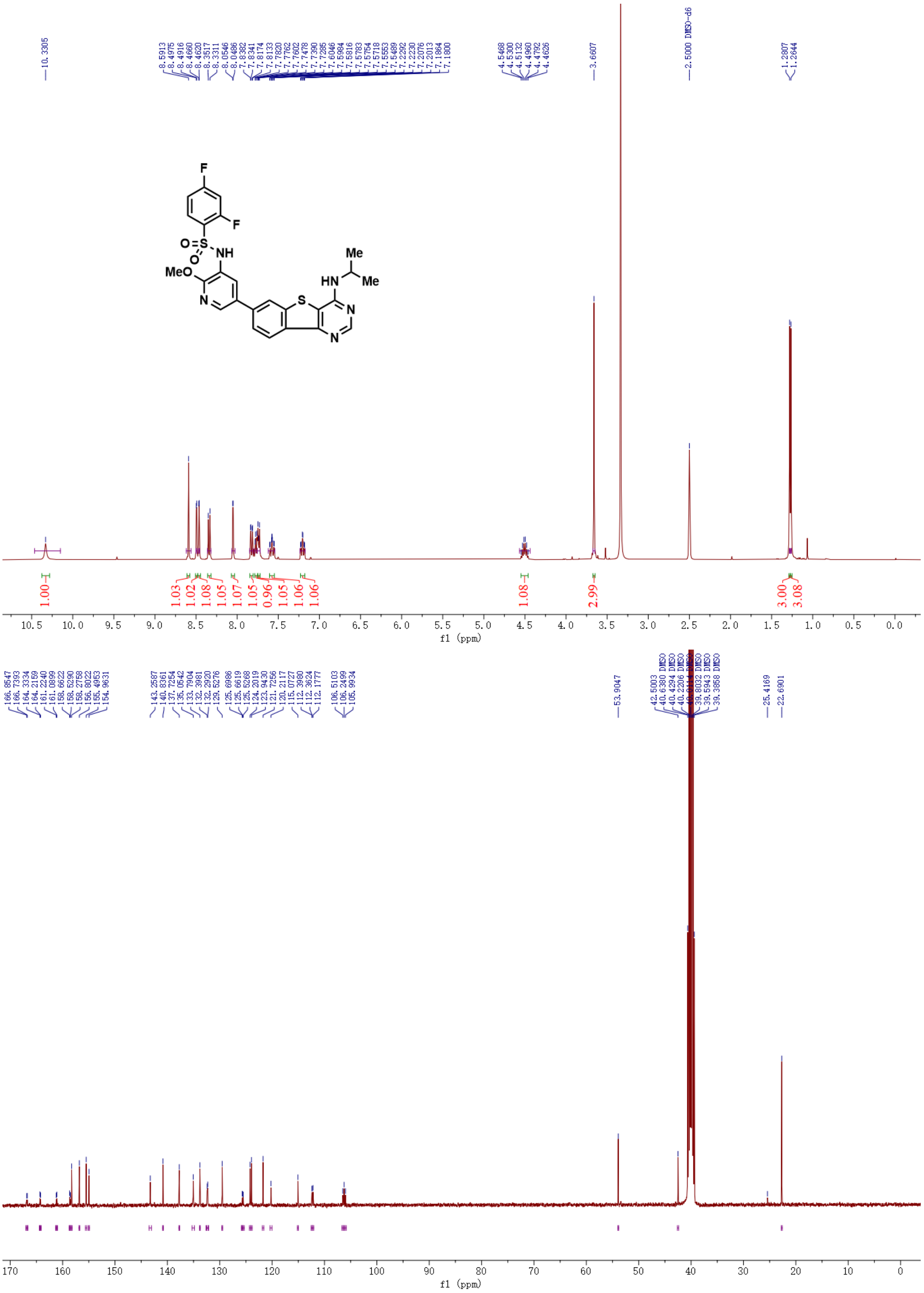

Figure S7. ^{1}H and ^{13}C NMR spectra of **11g** (DMSO-d6).

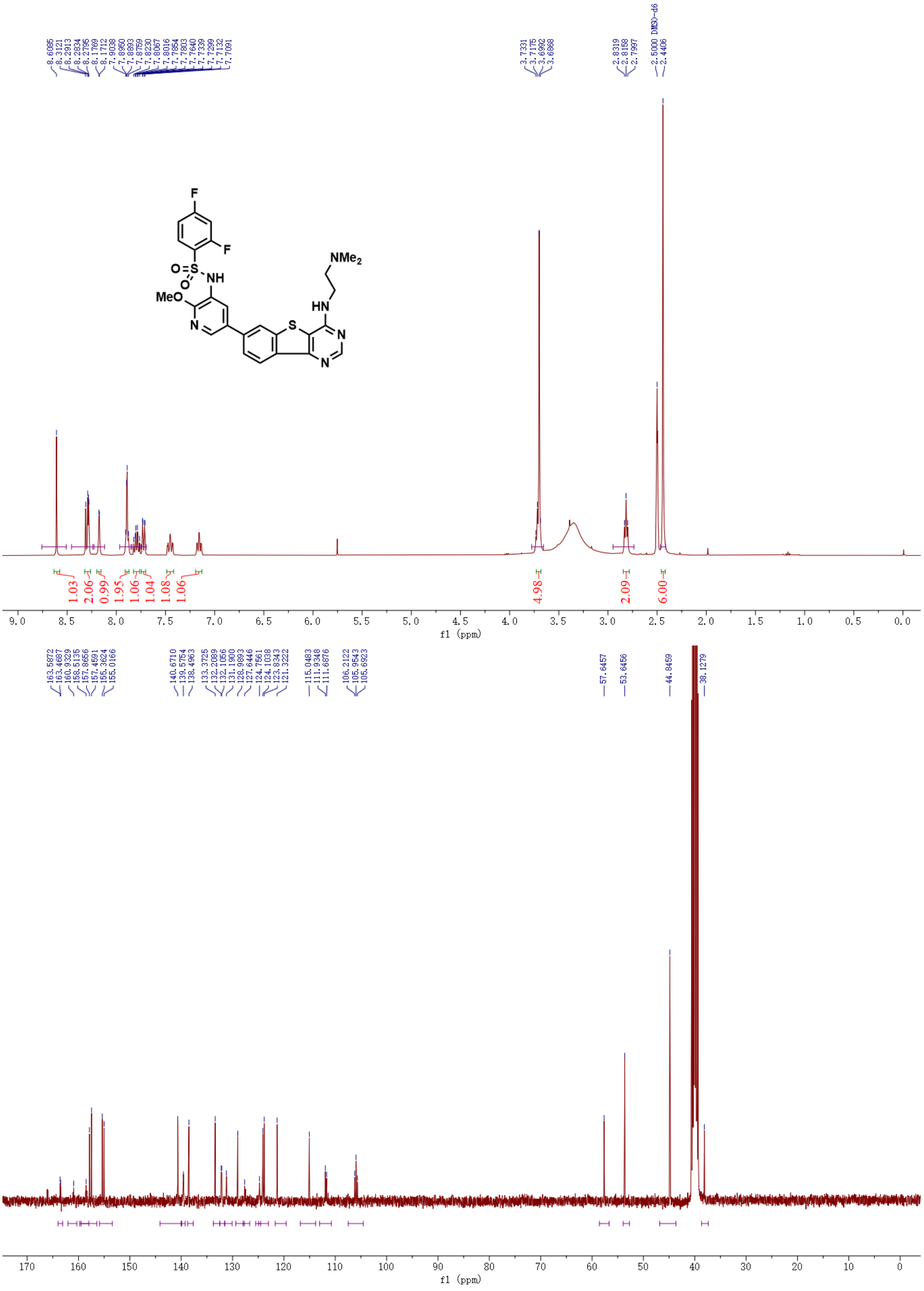

Figure S8 ^{1}H and ^{13}C NMR spectra of **11h** (DMSO-d6).

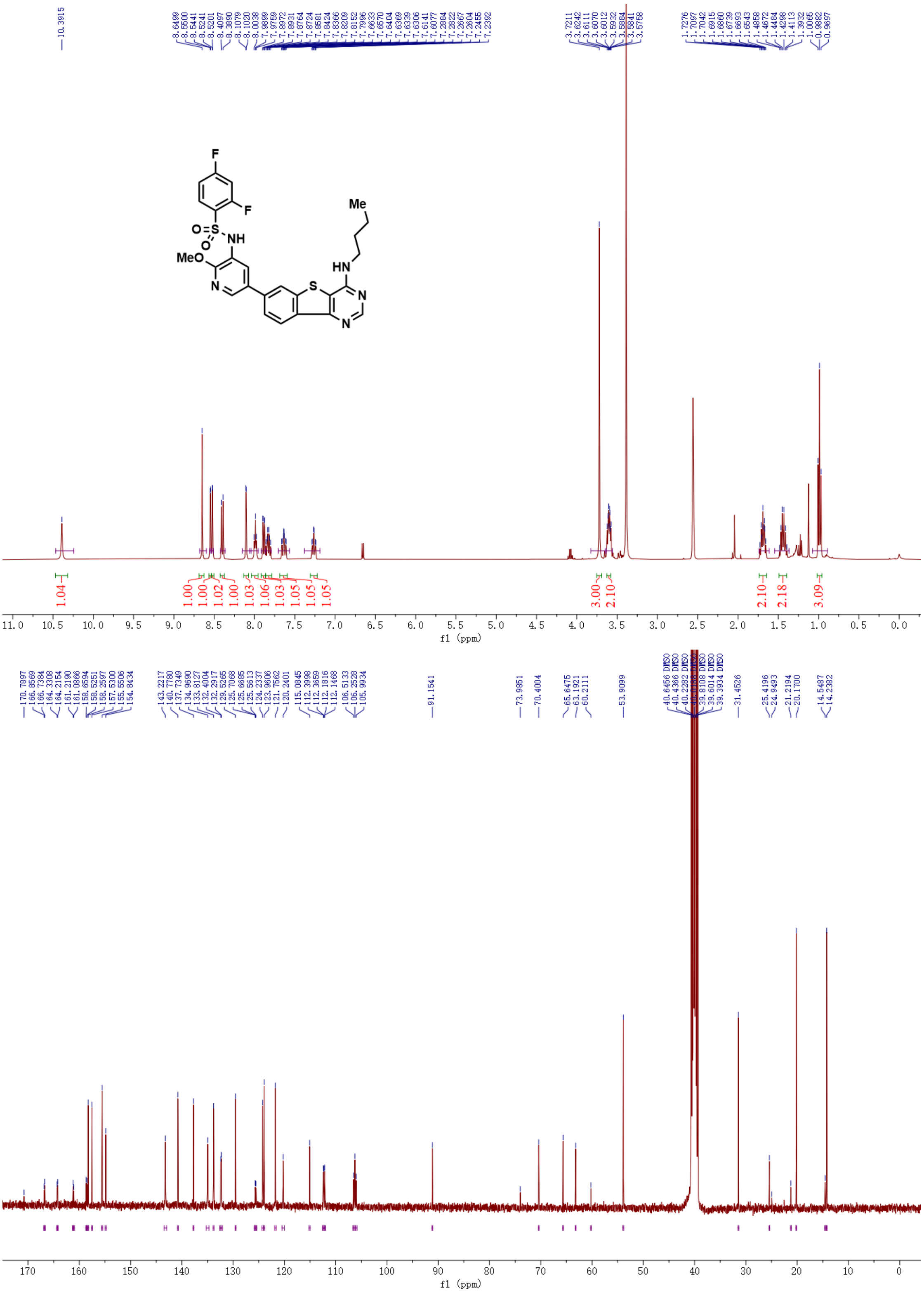

Figure S9. ^{1}H and ^{13}C NMR spectra of **11i** (DMSO-d6).

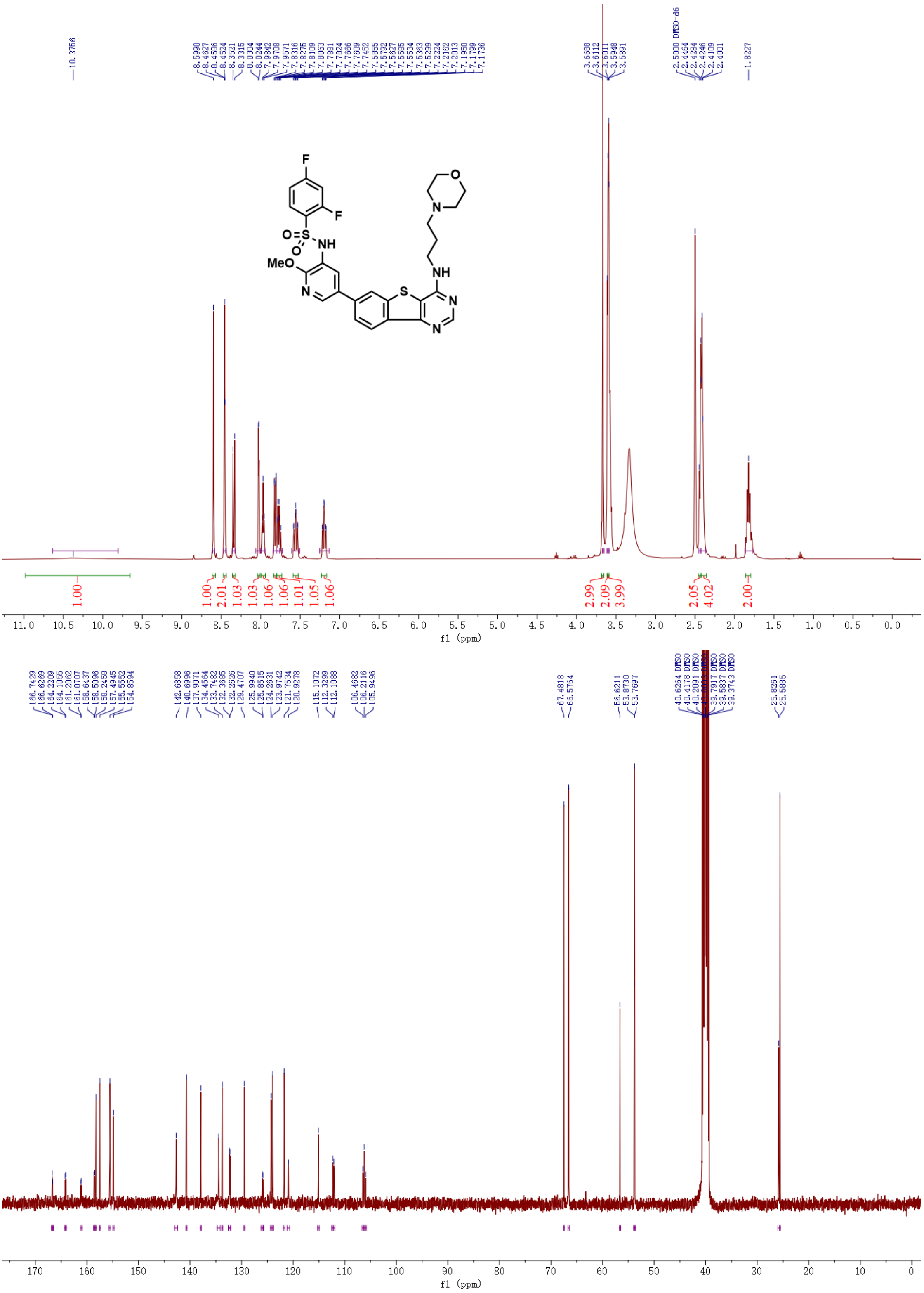

Figure S10. ^{1}H and ^{13}C NMR spectra of **11j** (DMSO-d6).

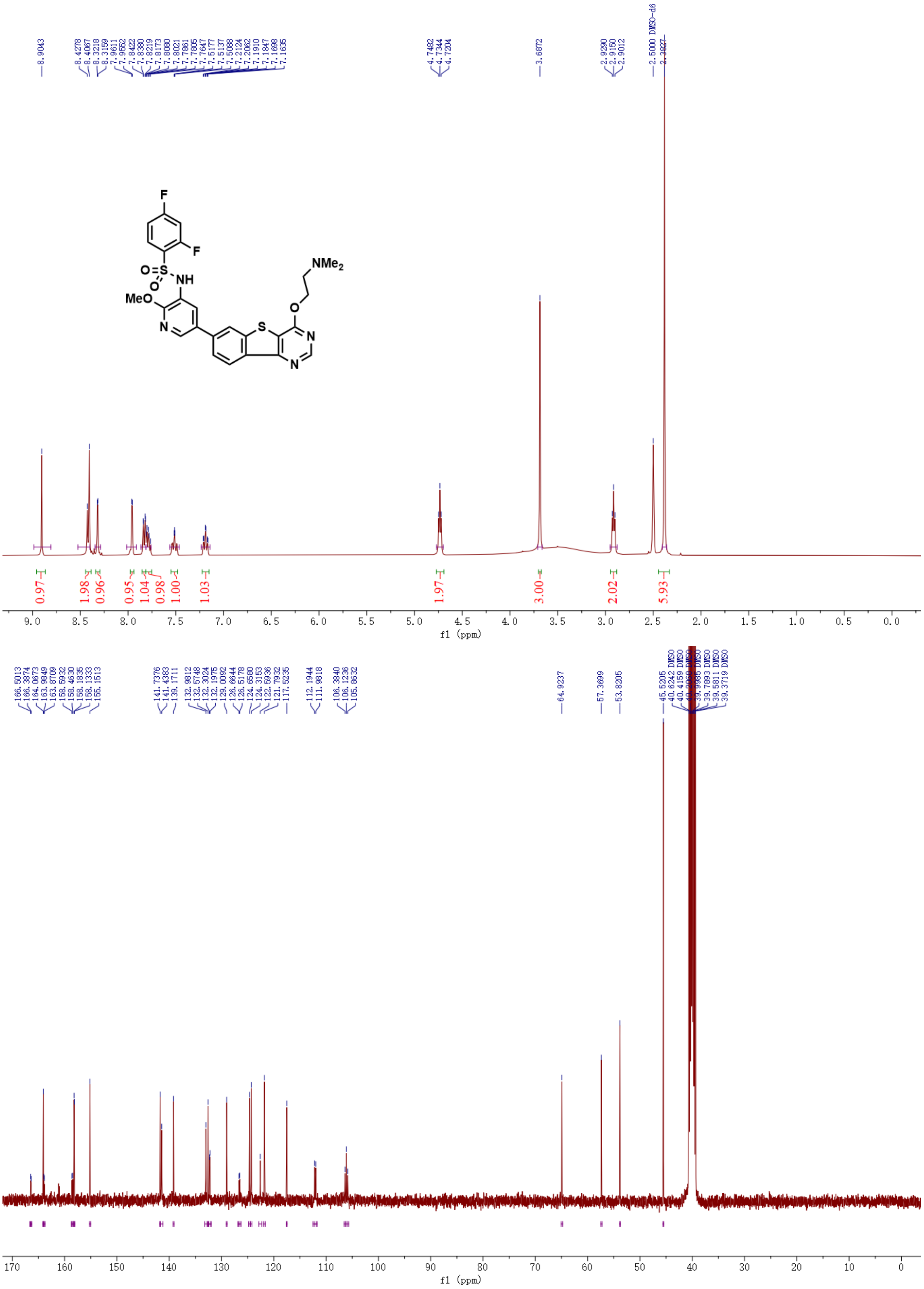

Figure S11. ^{1}H and ^{13}C NMR spectra of **11k** (DMSO-d6).

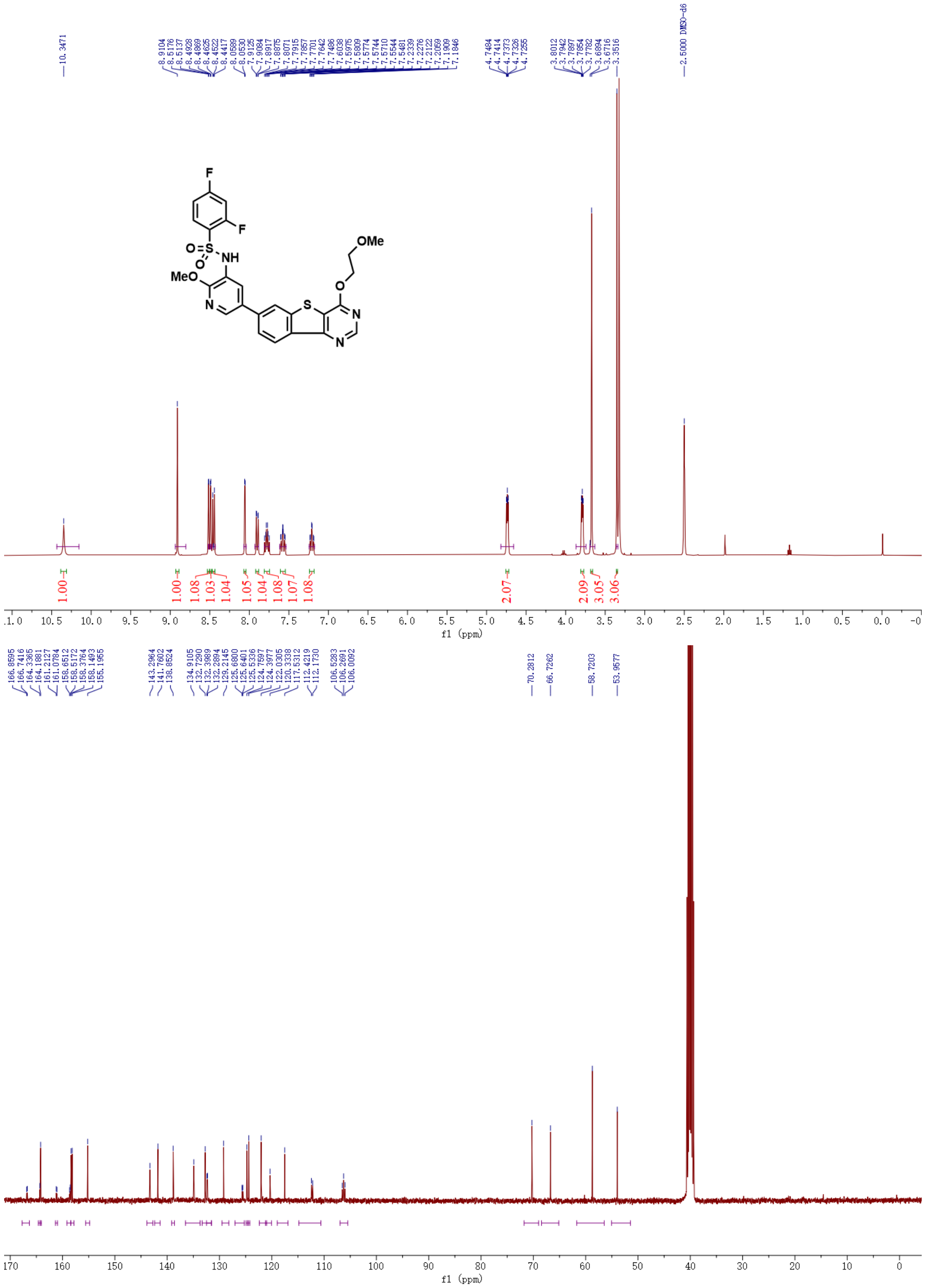

Figure S12. ^{1}H and ^{13}C NMR spectra of **11l** (DMSO-d6).

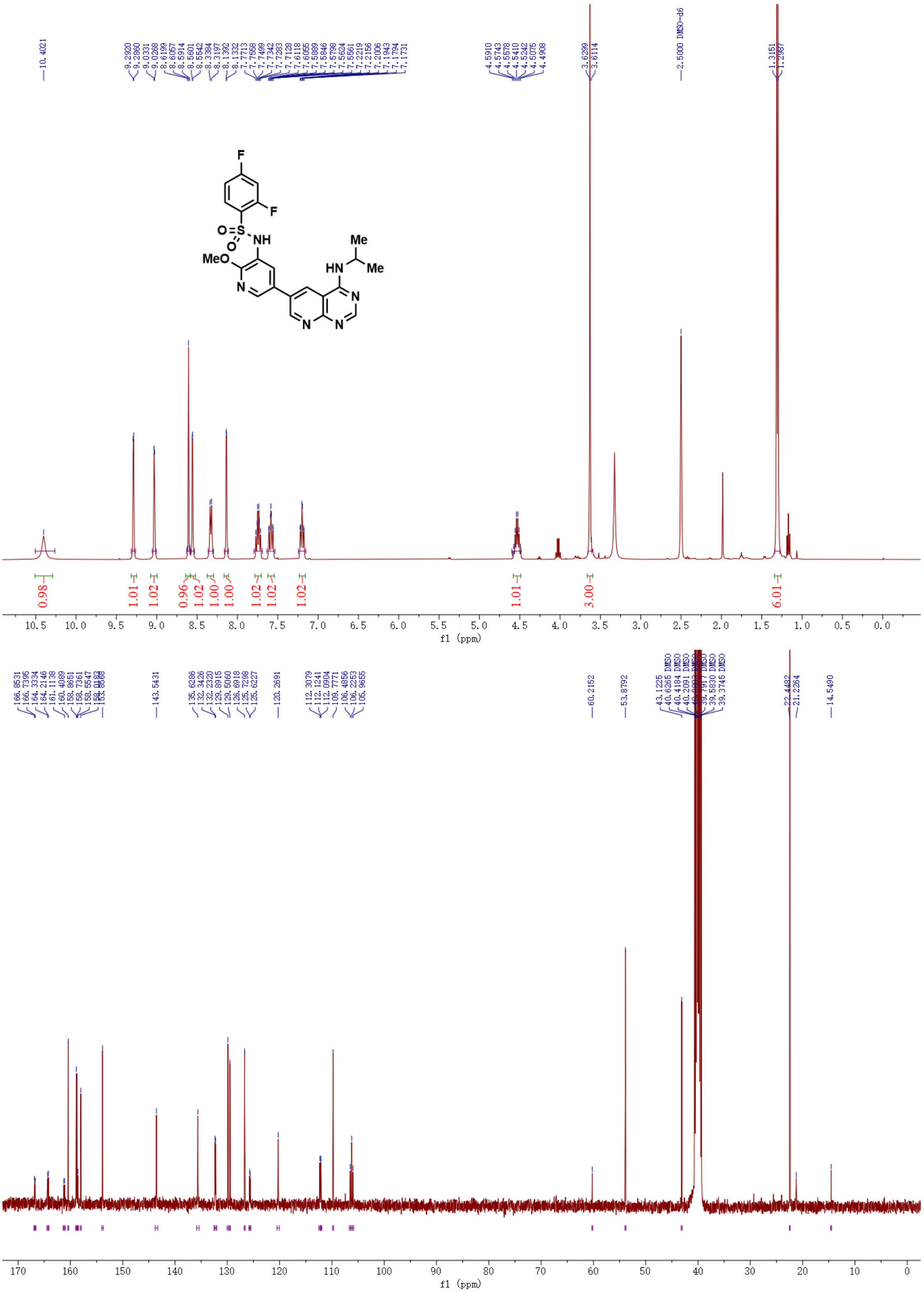

Figure S13. ^{1}H and ^{13}C NMR spectra of **17a** (DMSO-d6).

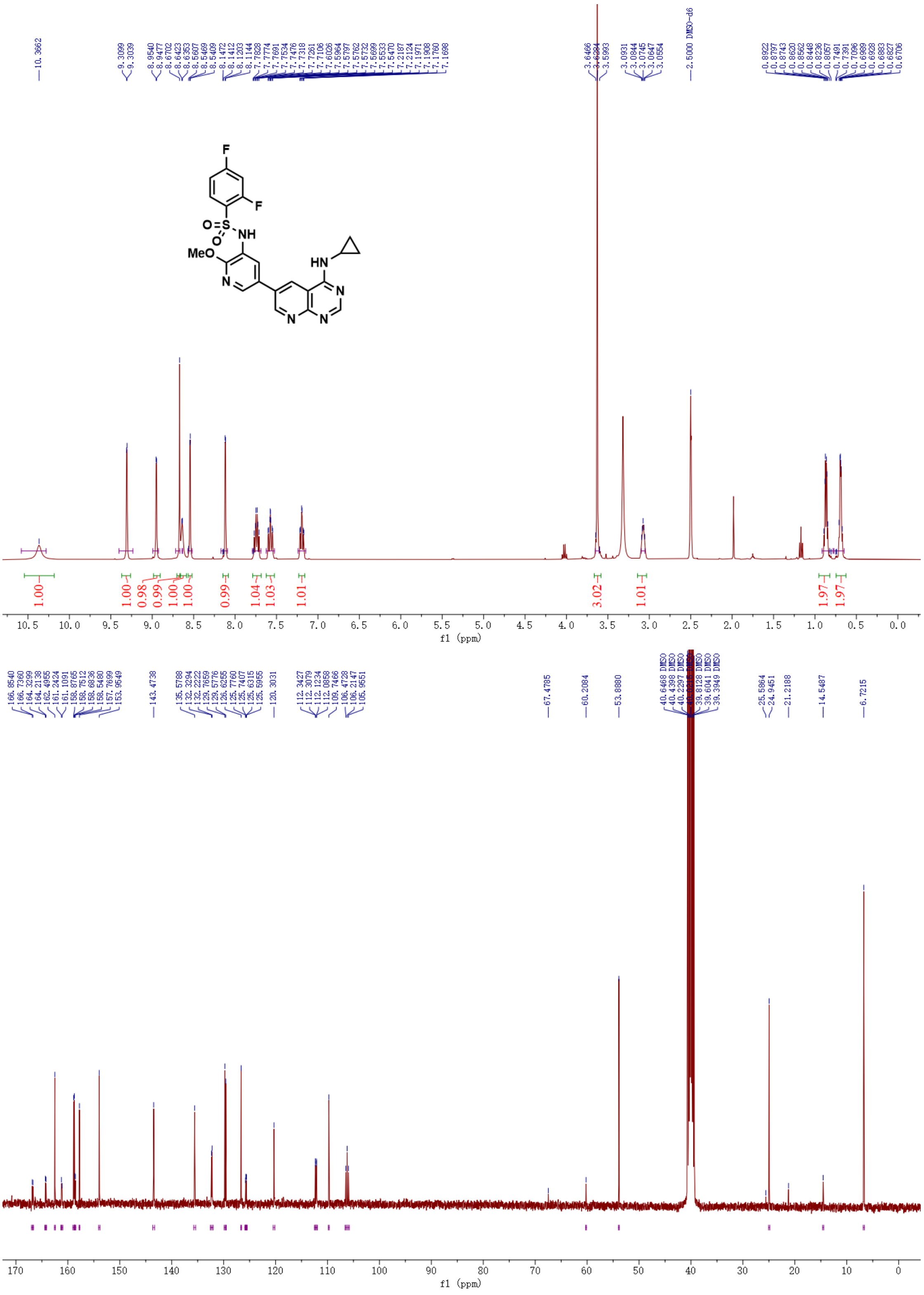

Figure S14. ^{1}H and ^{13}C NMR spectra of **17b** (DMSO-d6).

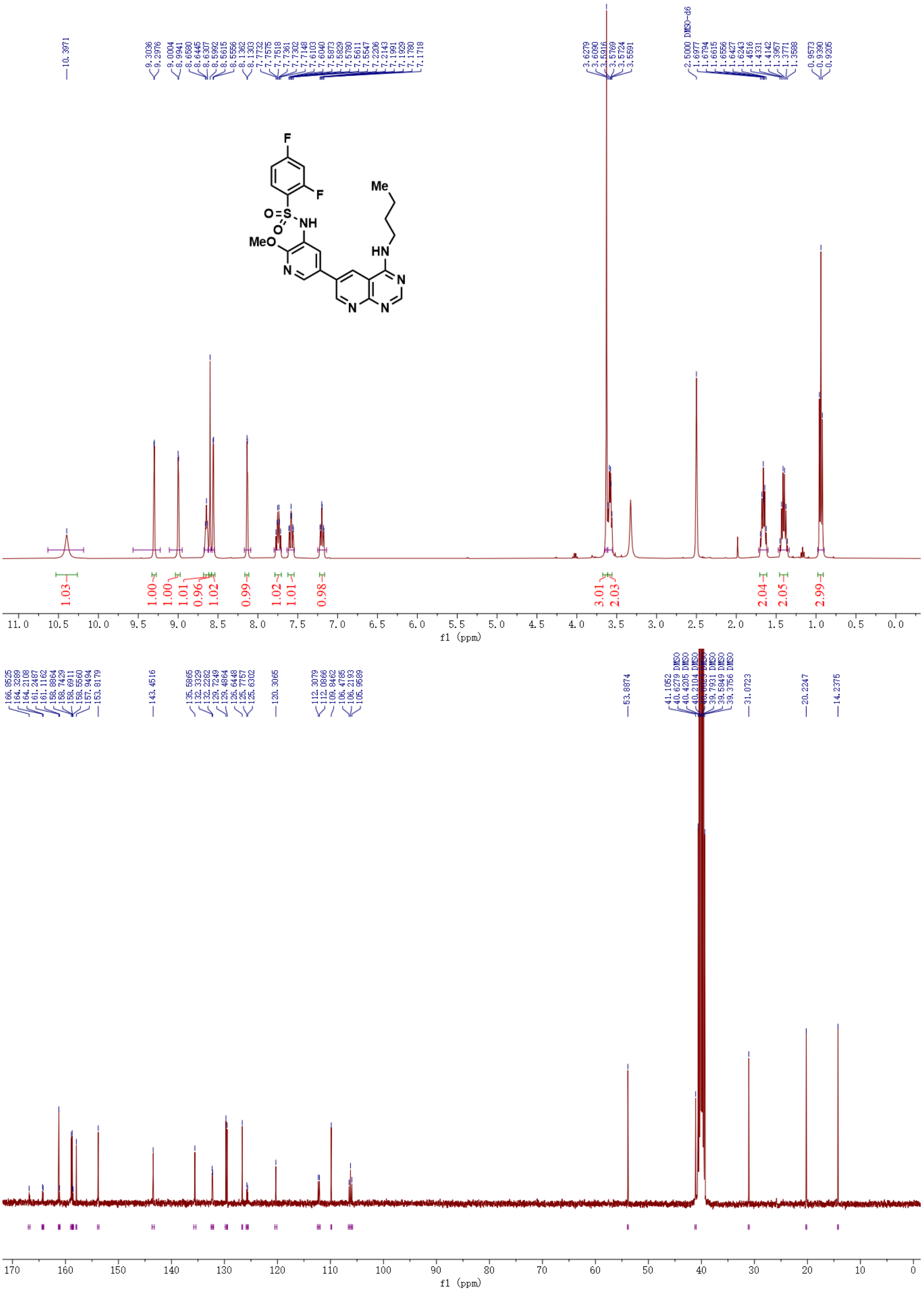

Figure S15 ^{1}H and ^{13}C NMR spectra of **17c** (DMSO-d6).

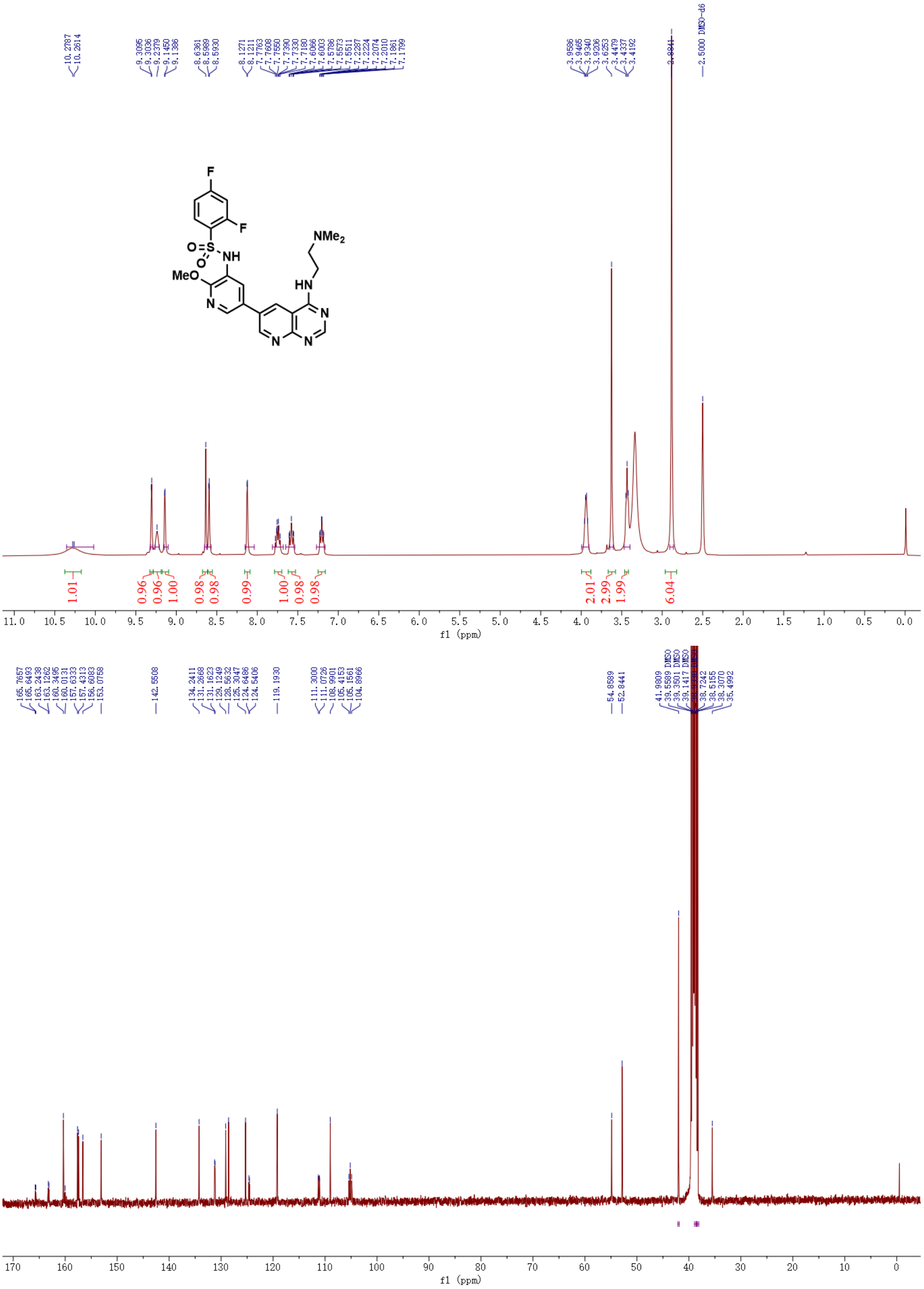

Figure S16. ^{1}H and ^{13}C NMR spectra of **17d** (DMSO-d6).

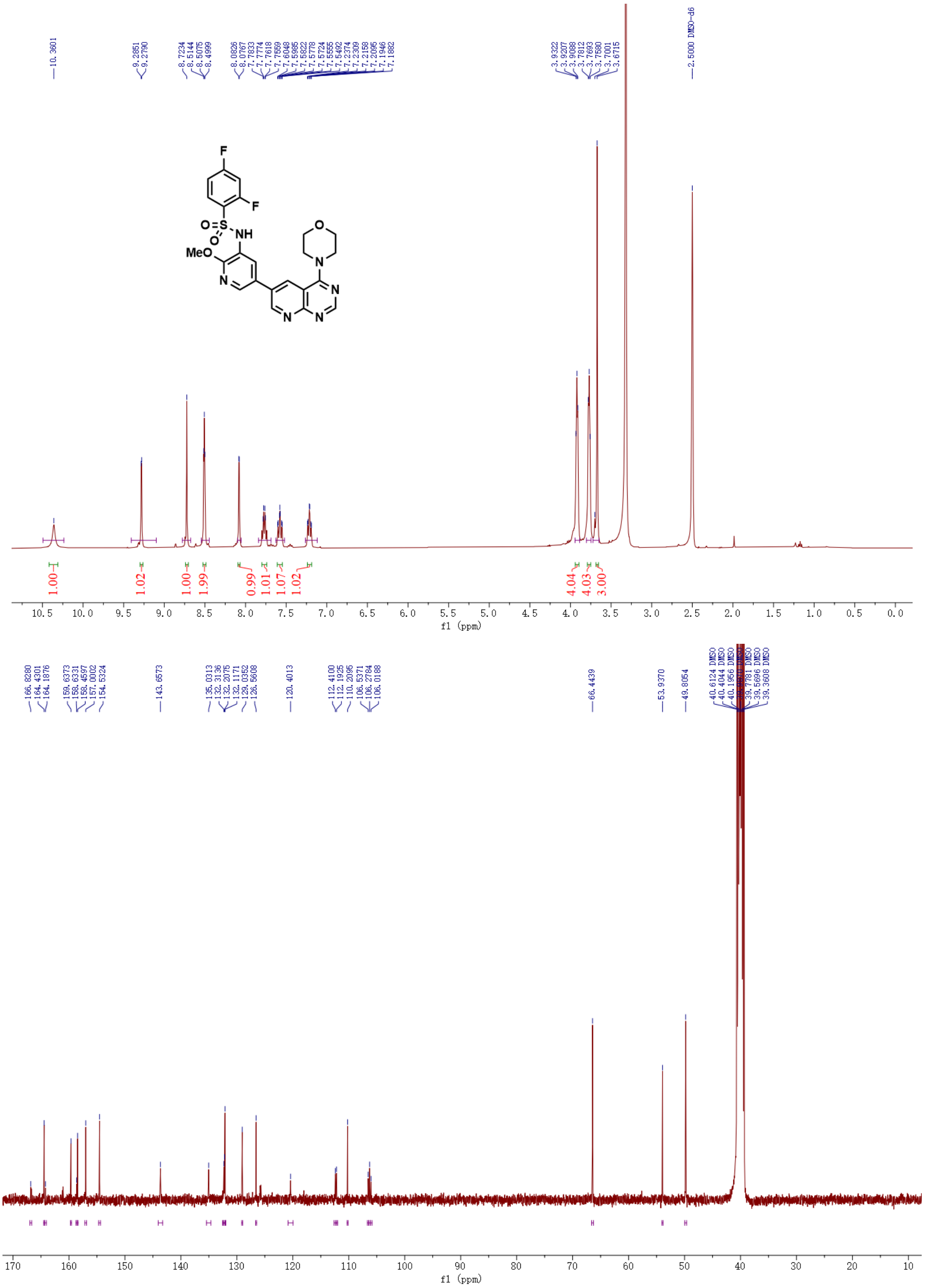

Figure S17. ^{1}H and ^{13}C NMR spectra of **17e** (DMSO-d6).

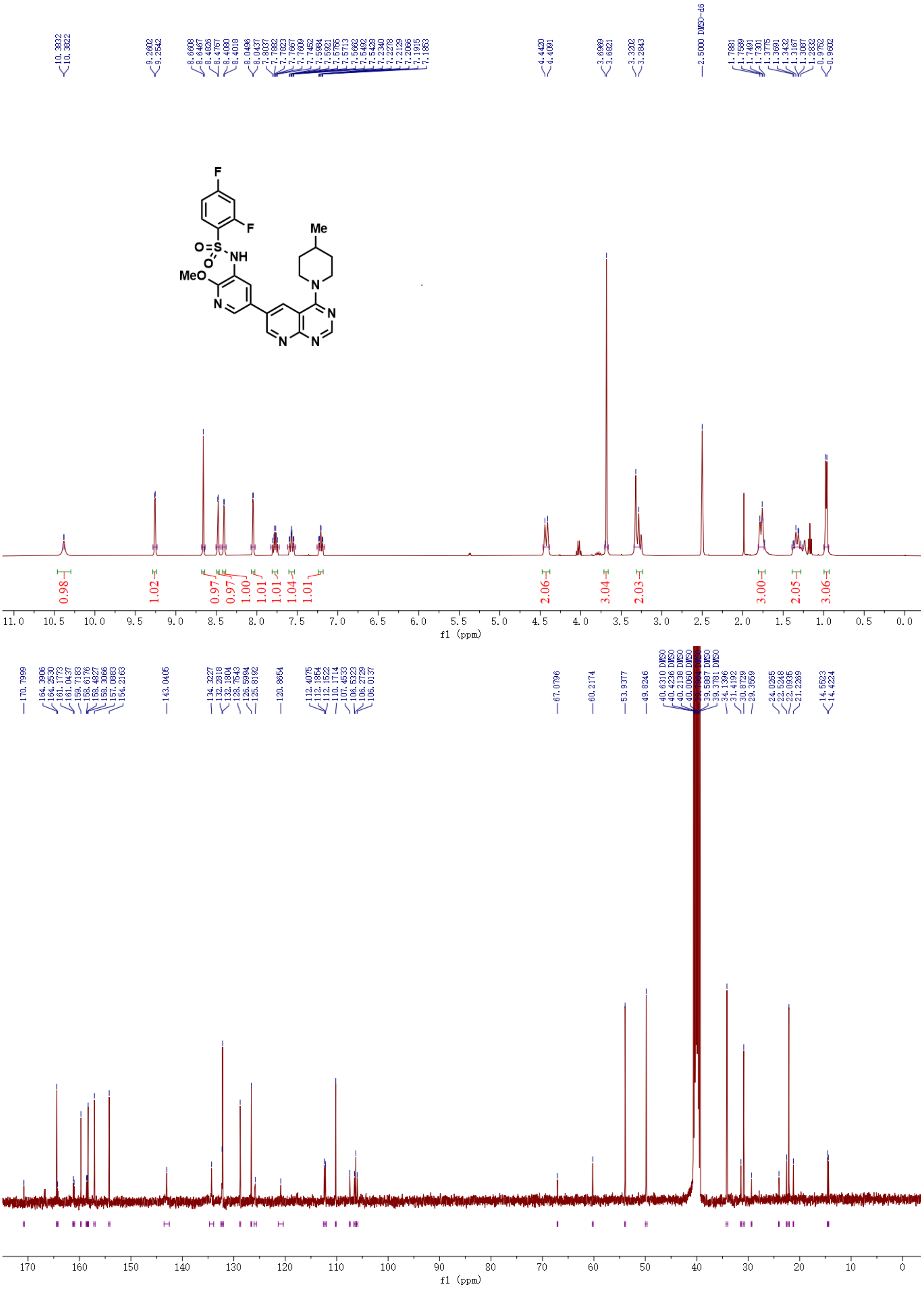

Figure S18. ^{1}H and ^{13}C NMR spectra of **17f** (DMSO-d6).

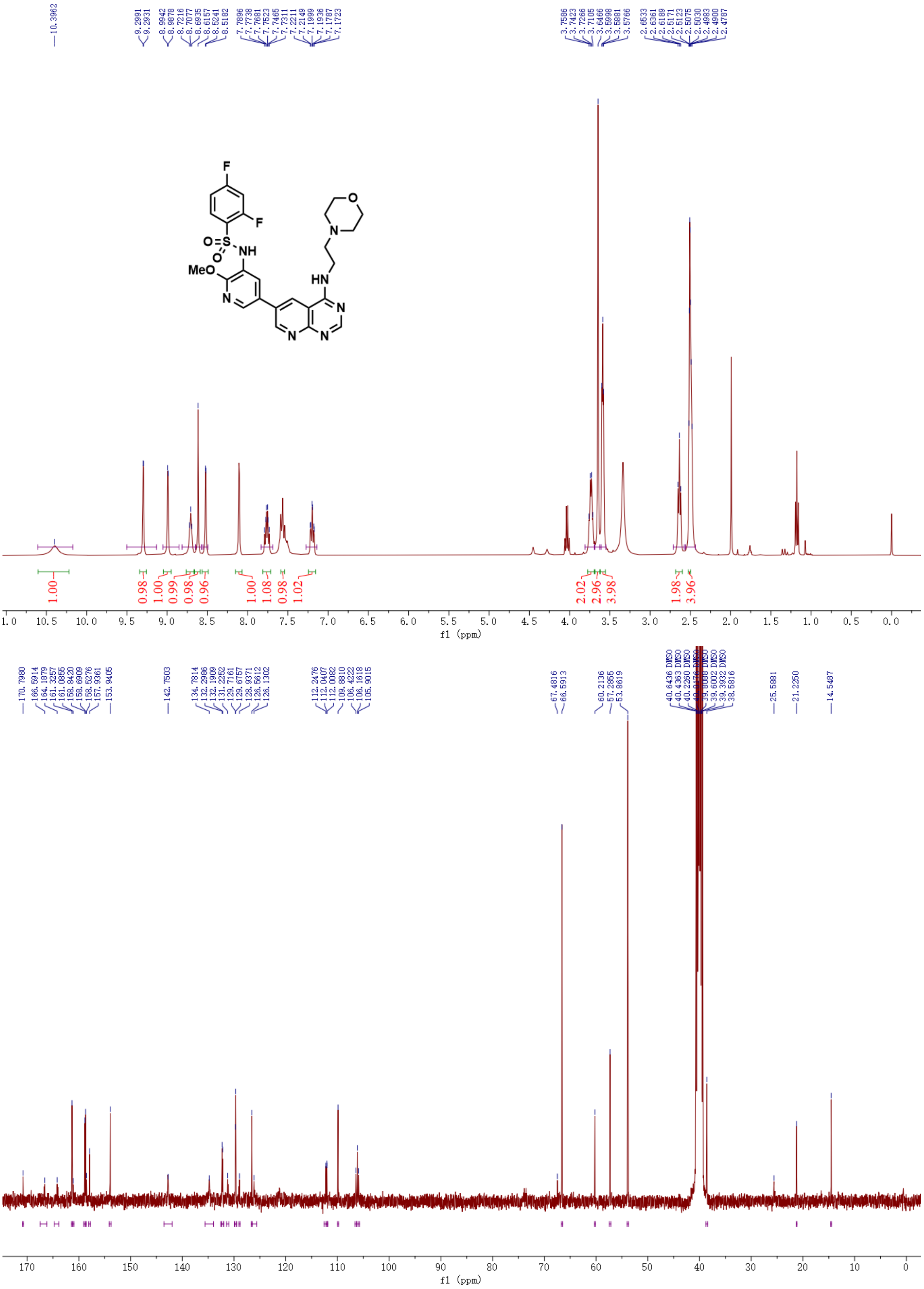

Figure S19. ^{1}H and ^{13}C NMR spectra of **17g** (DMSO-d6).

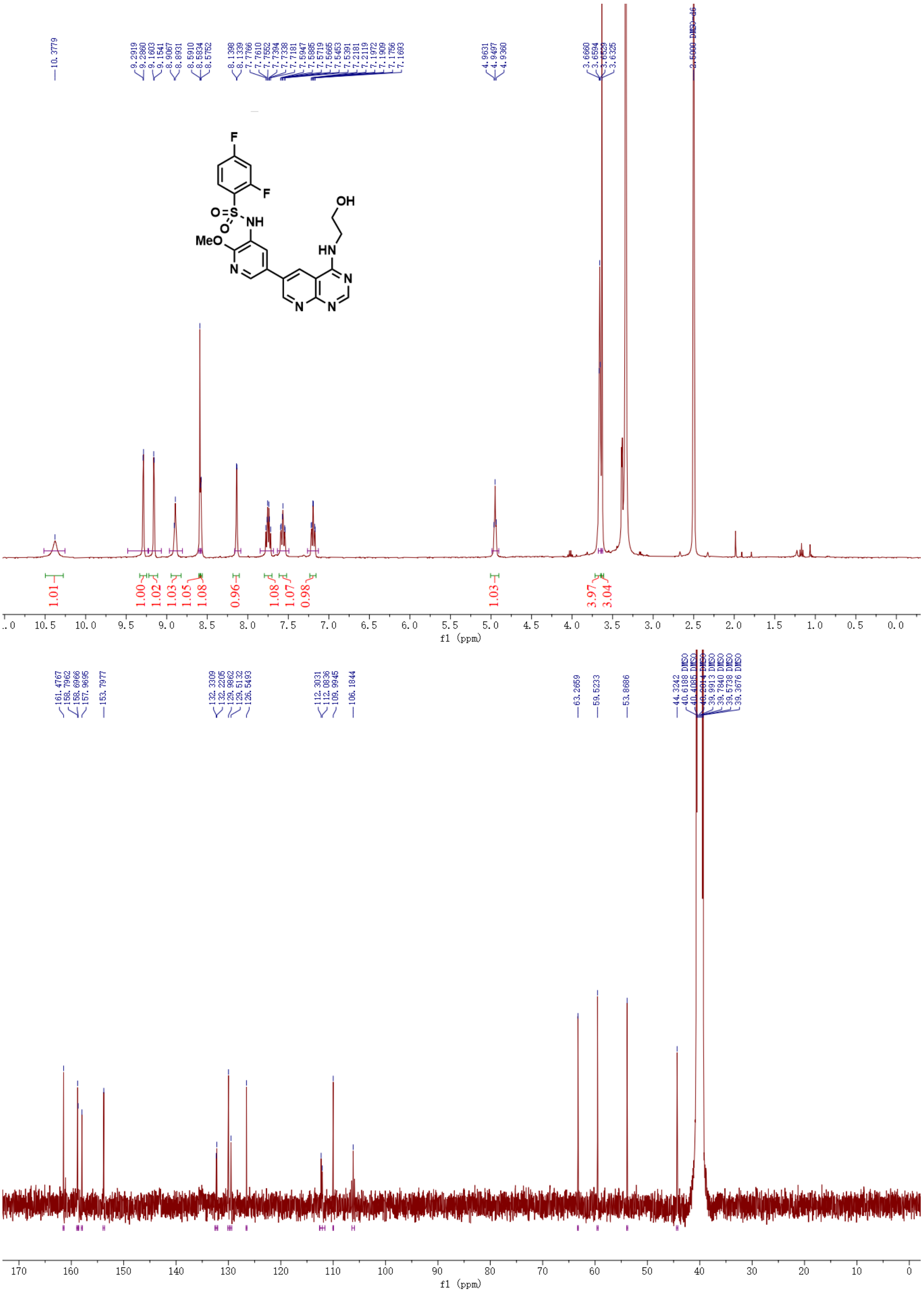

Figure S20. ^{1}H and ^{13}C NMR spectra of **17h** (DMSO-d6).

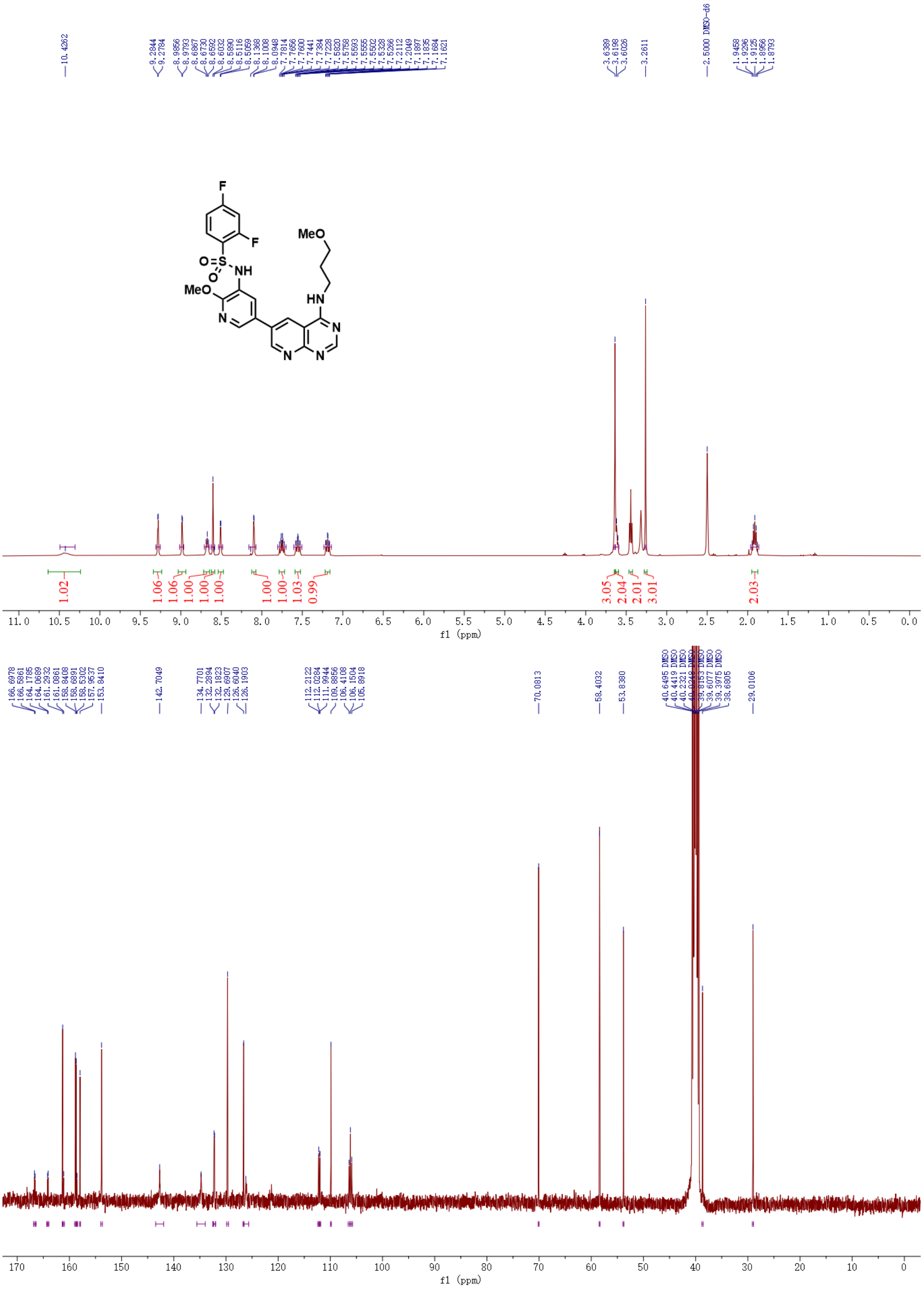

Figure S21. ^{1}H and ^{13}C NMR spectra of **17i** (DMSO-d6).

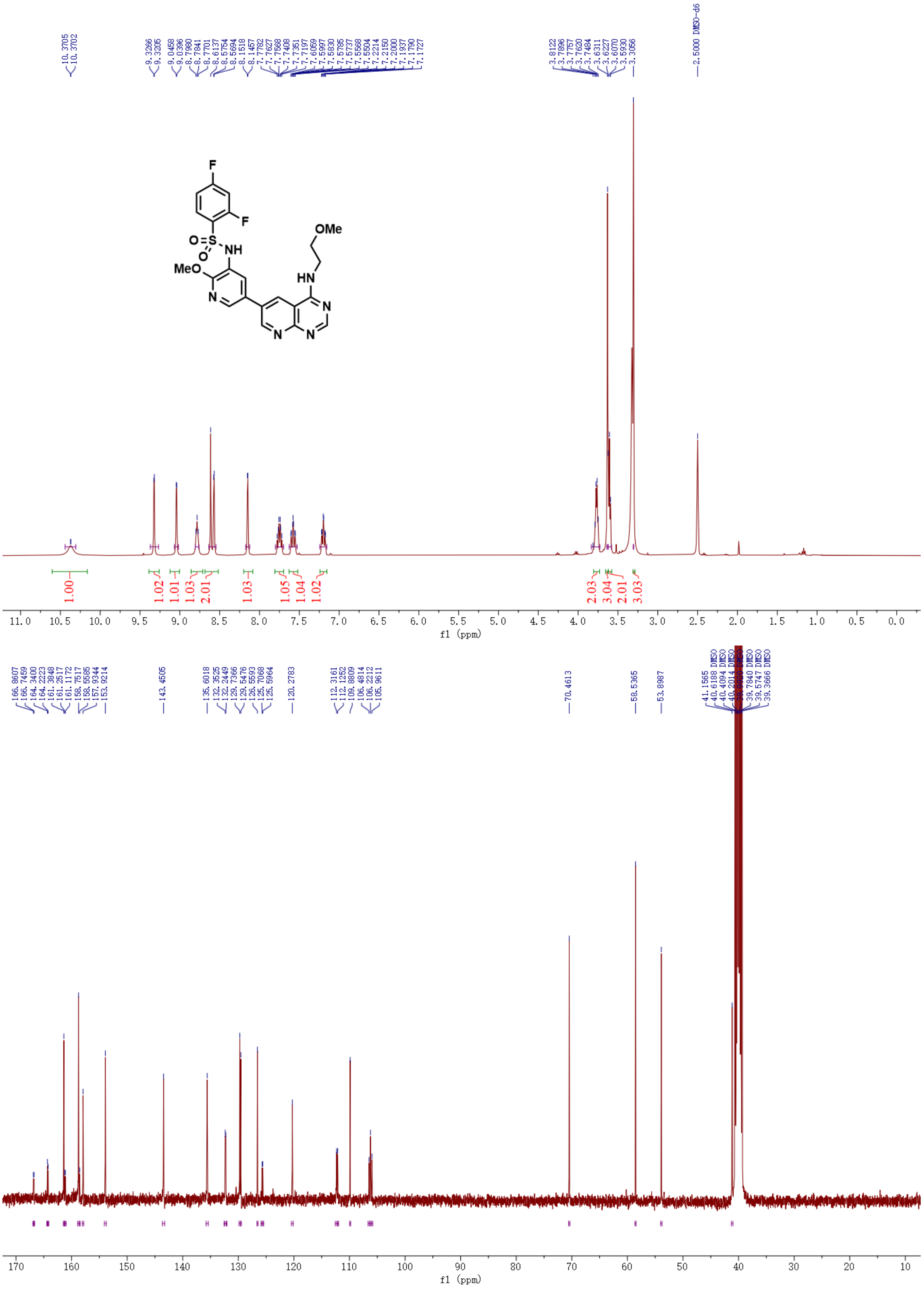

Figure S22. ^{1}H and ^{13}C NMR spectra of **17j** (DMSO-d6).

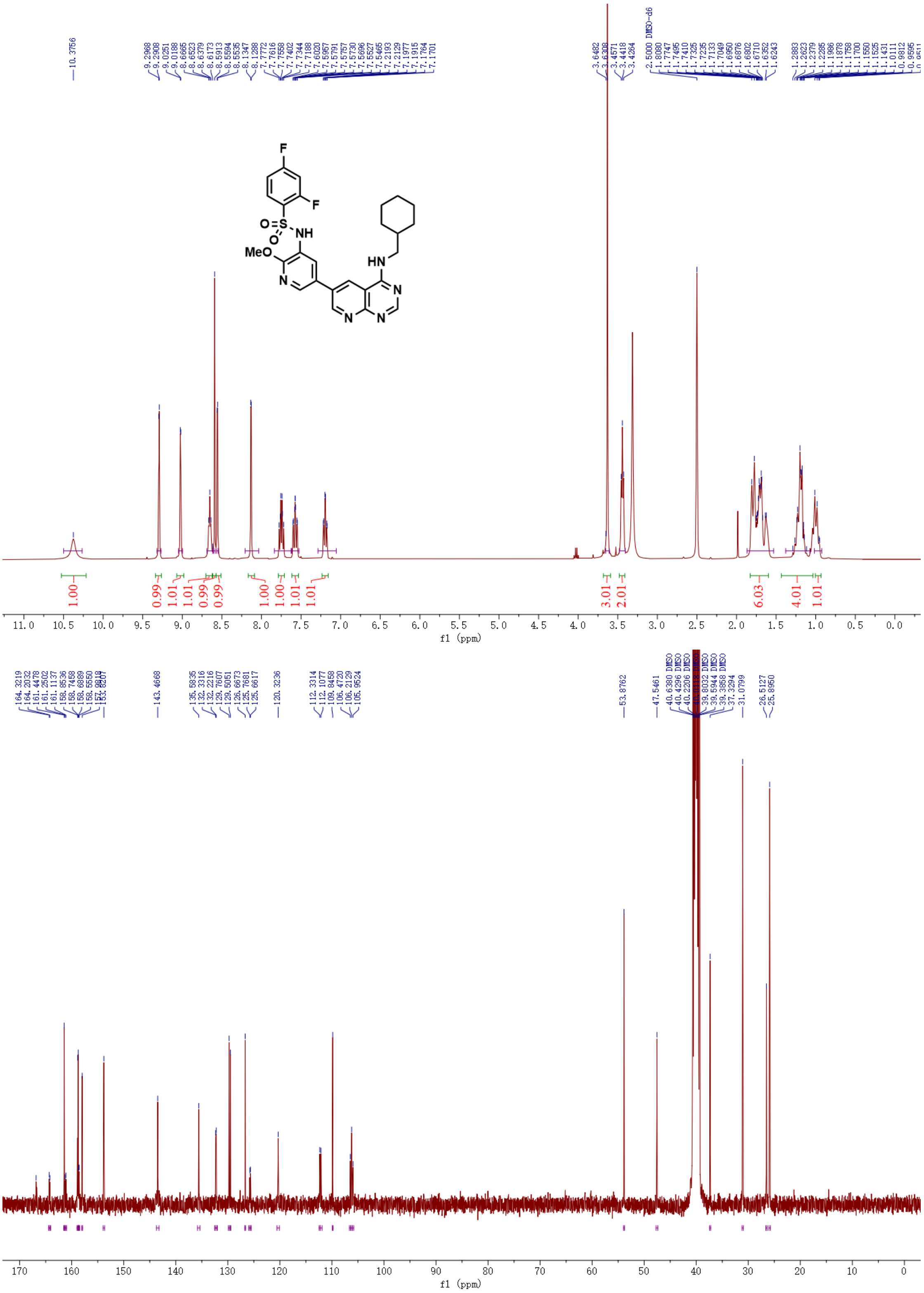

Figure S23. ^{1}H and ^{13}C NMR spectra of **17k** (DMSO-d6).

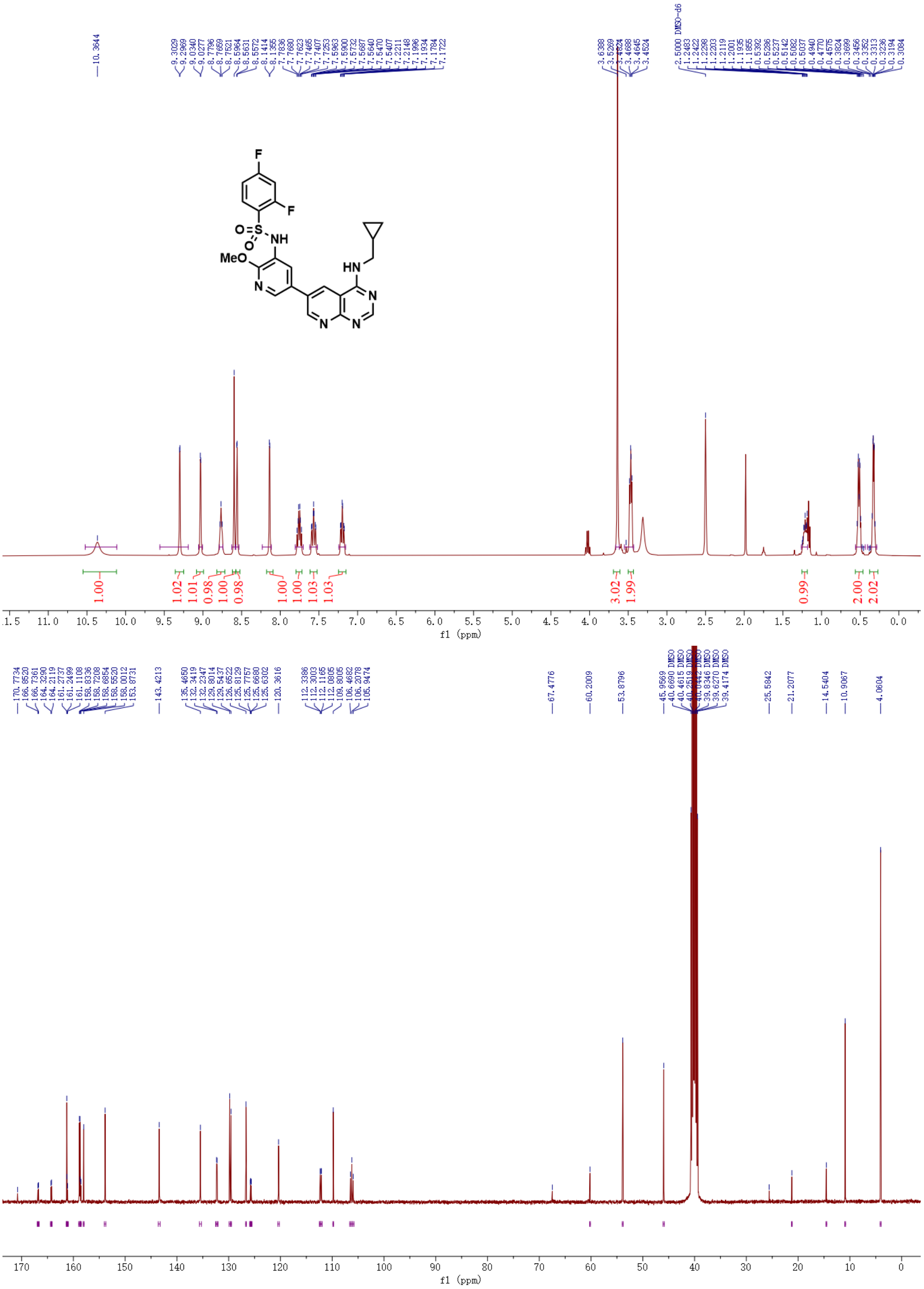

Figure S24. ^{1}H and ^{13}C NMR spectra of **17l** (DMSO-d6).

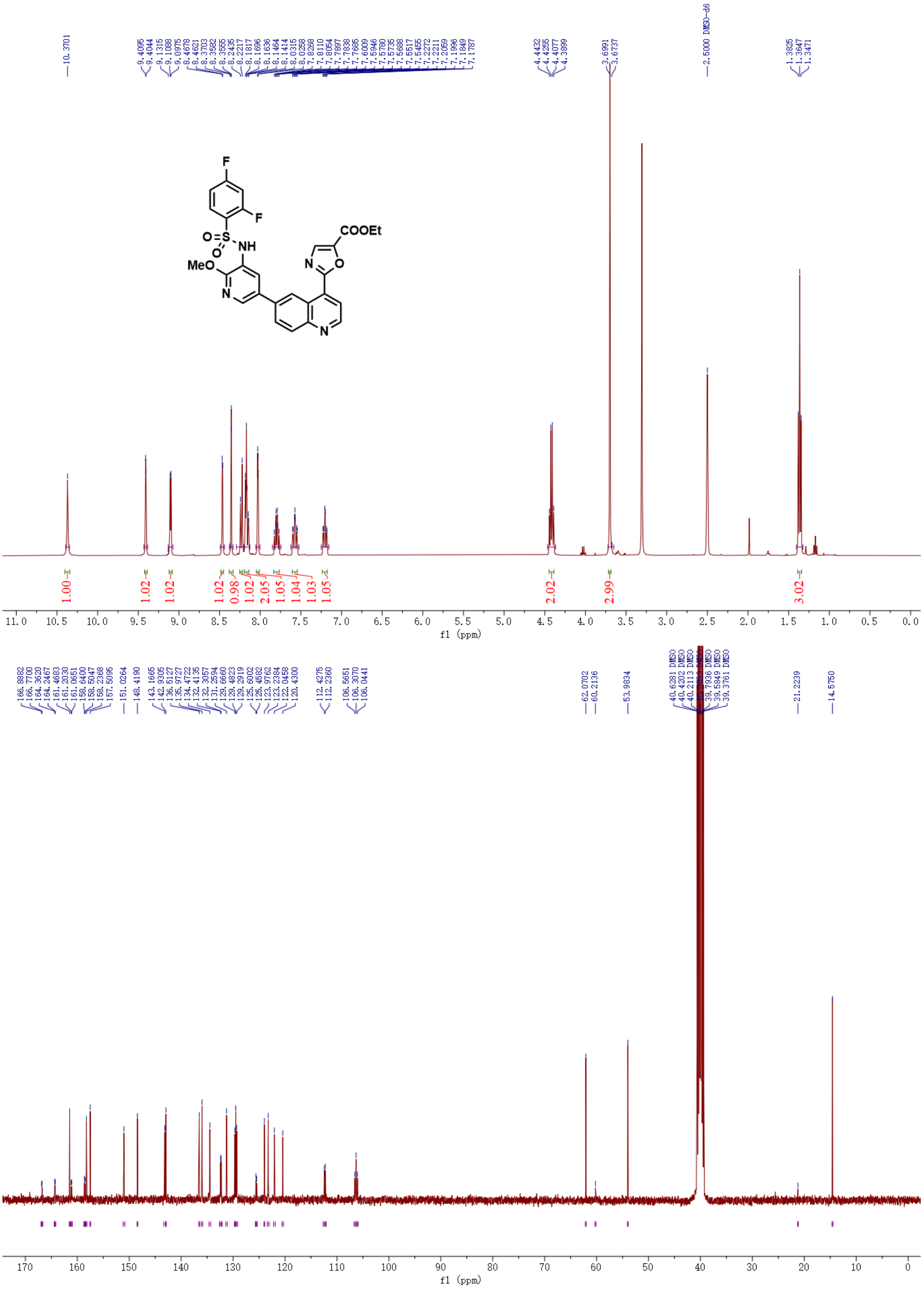

Figure S25. ^{1}H and ^{13}C NMR spectra of **22a** (DMSO-d6).

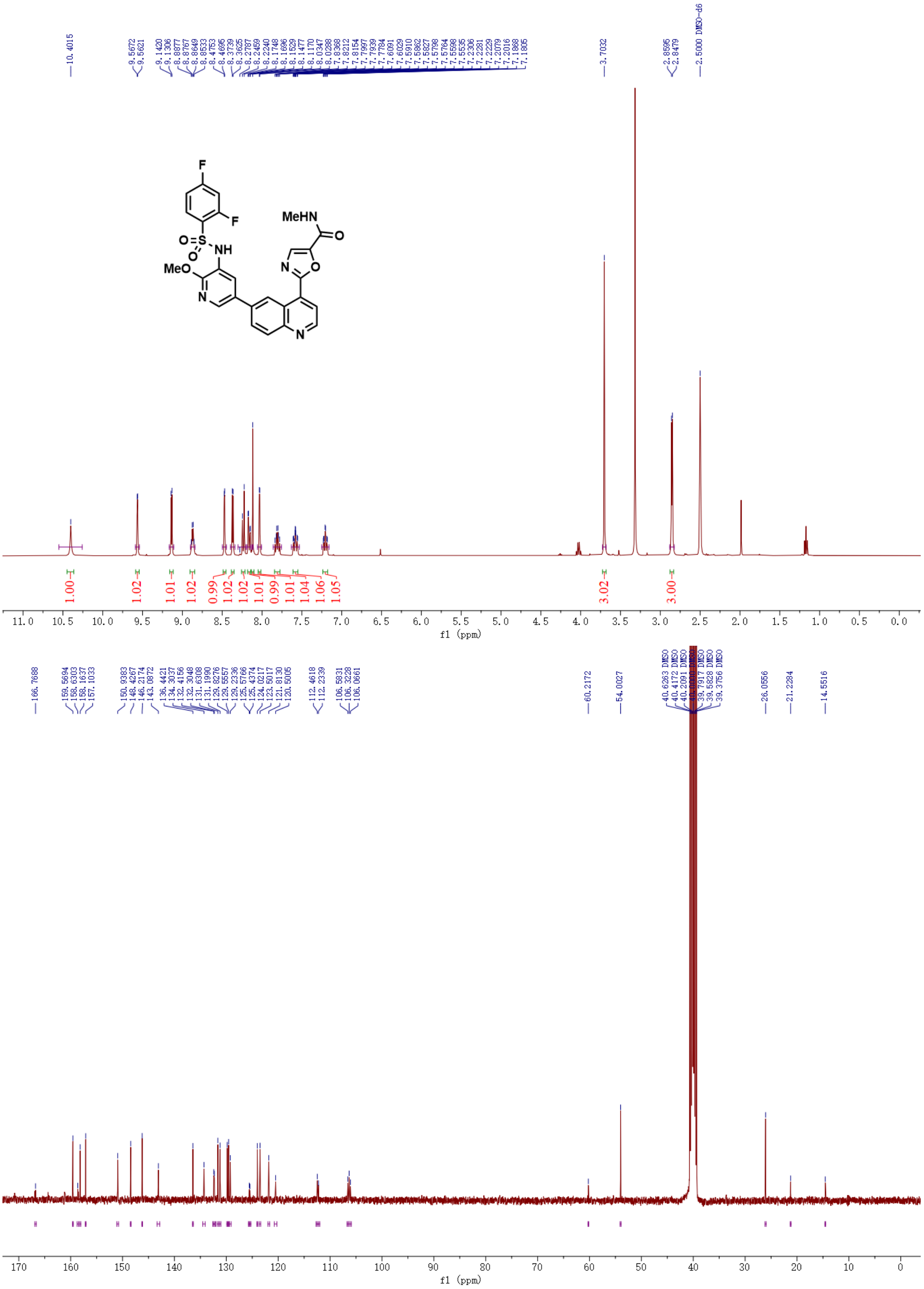

Figure S26. ^{1}H and ^{13}C NMR spectra of **22b** (DMSO-d6).

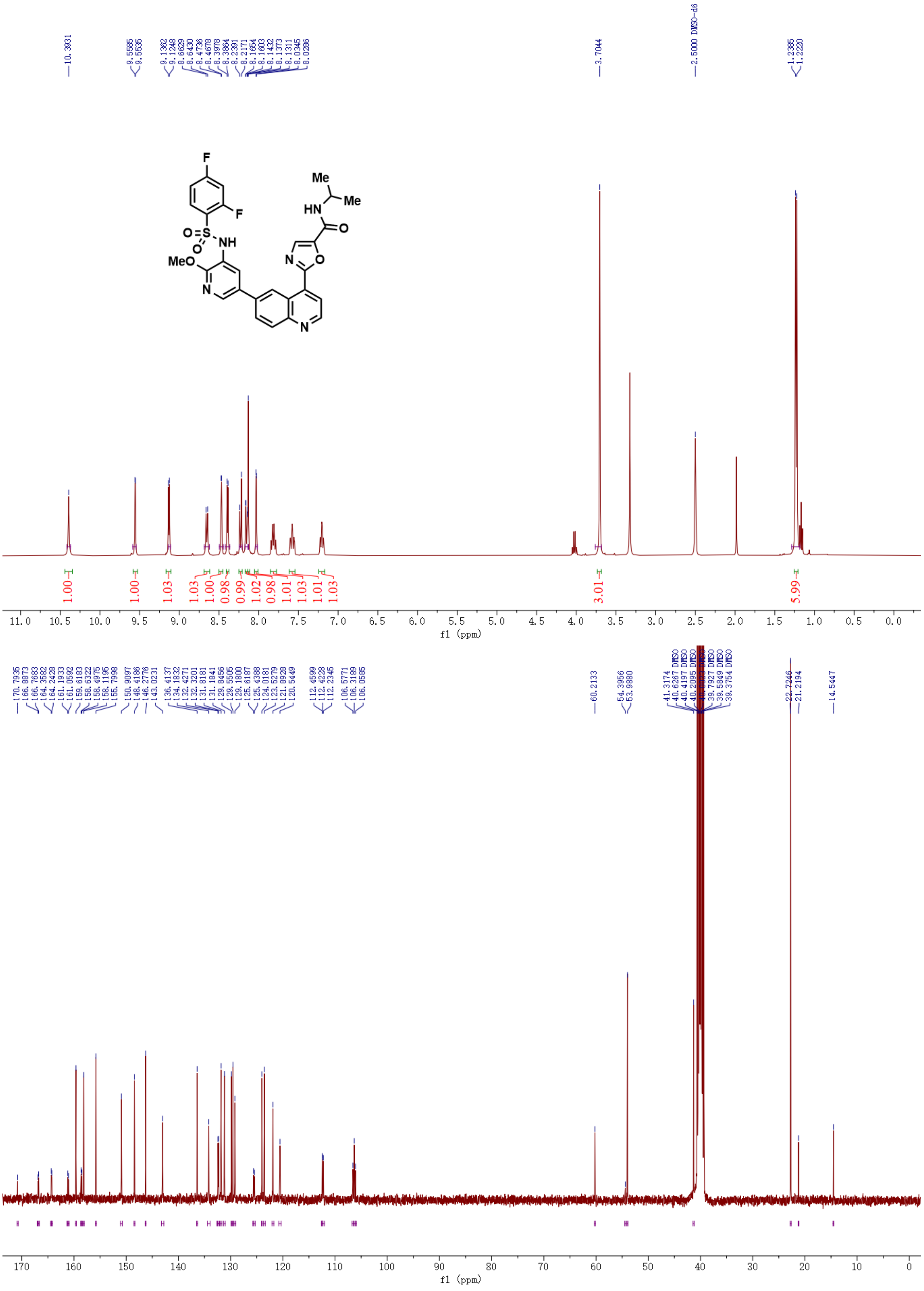

Figure S27. ^{1}H and ^{13}C NMR spectra of **22c** (DMSO-d6).

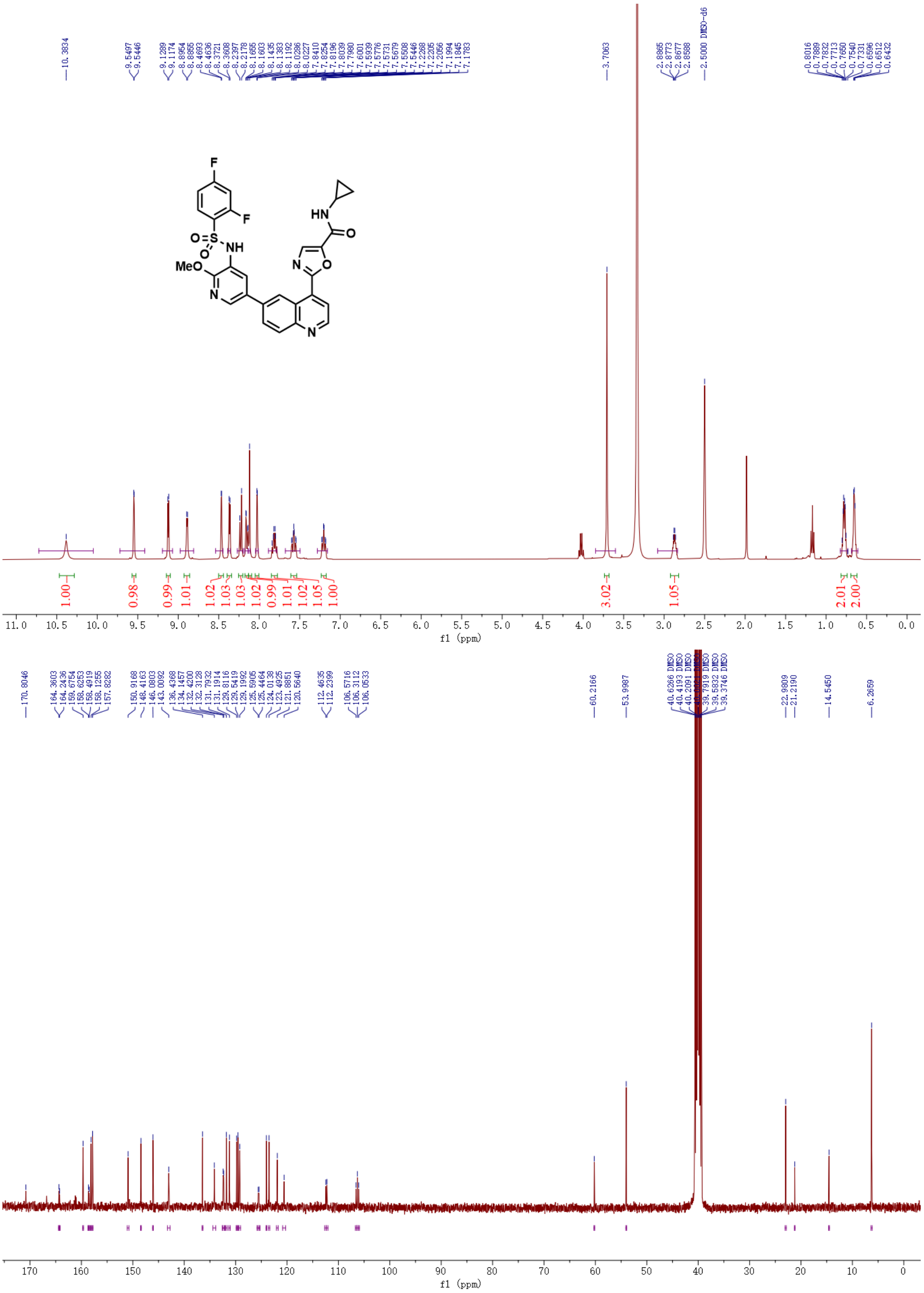

Figure S28. ^{1}H and ^{13}C NMR spectra of **22d** (DMSO-d6).

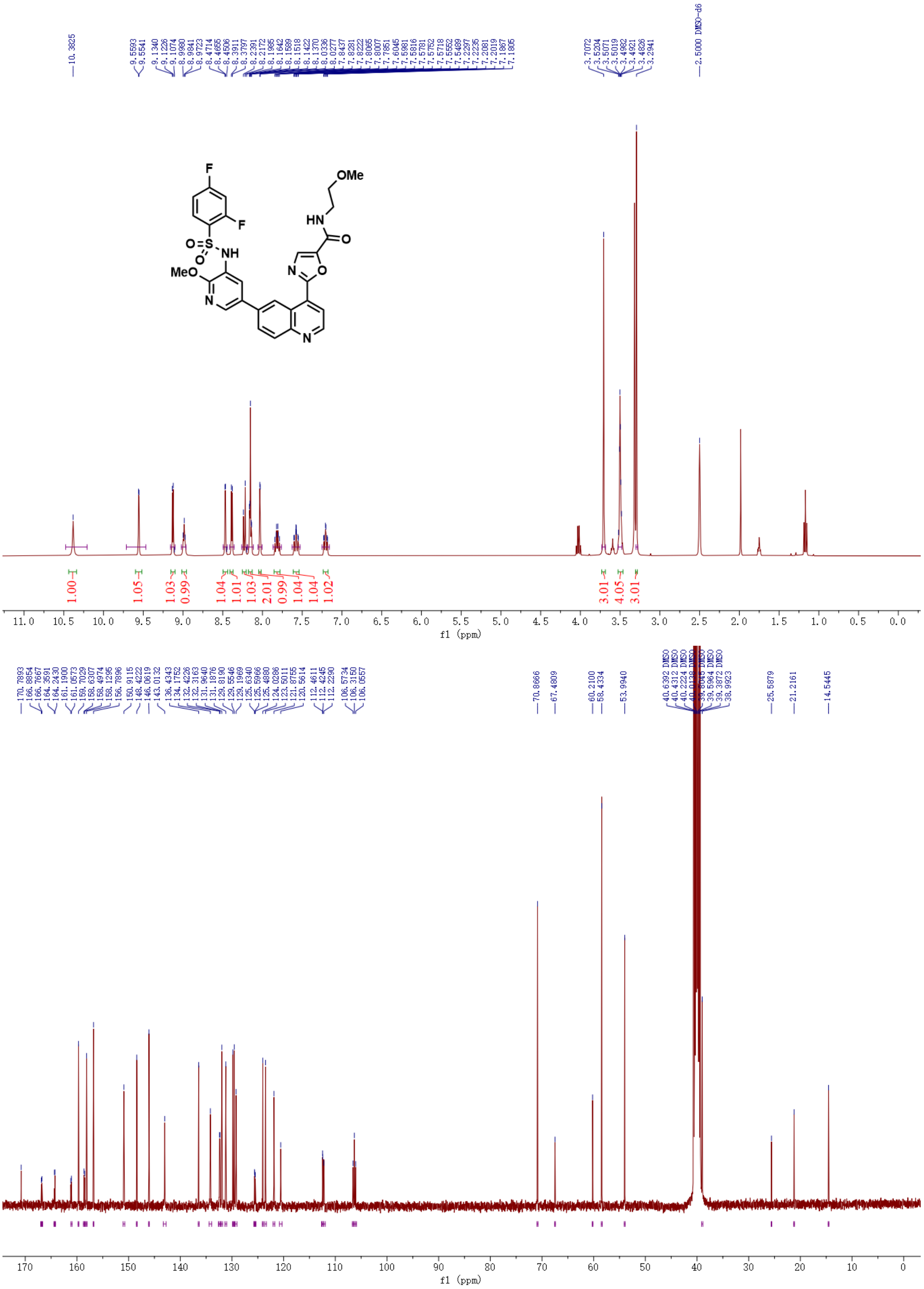

Figure S29. ^{1}H and ^{13}C NMR spectra of **22e** (DMSO-d6).

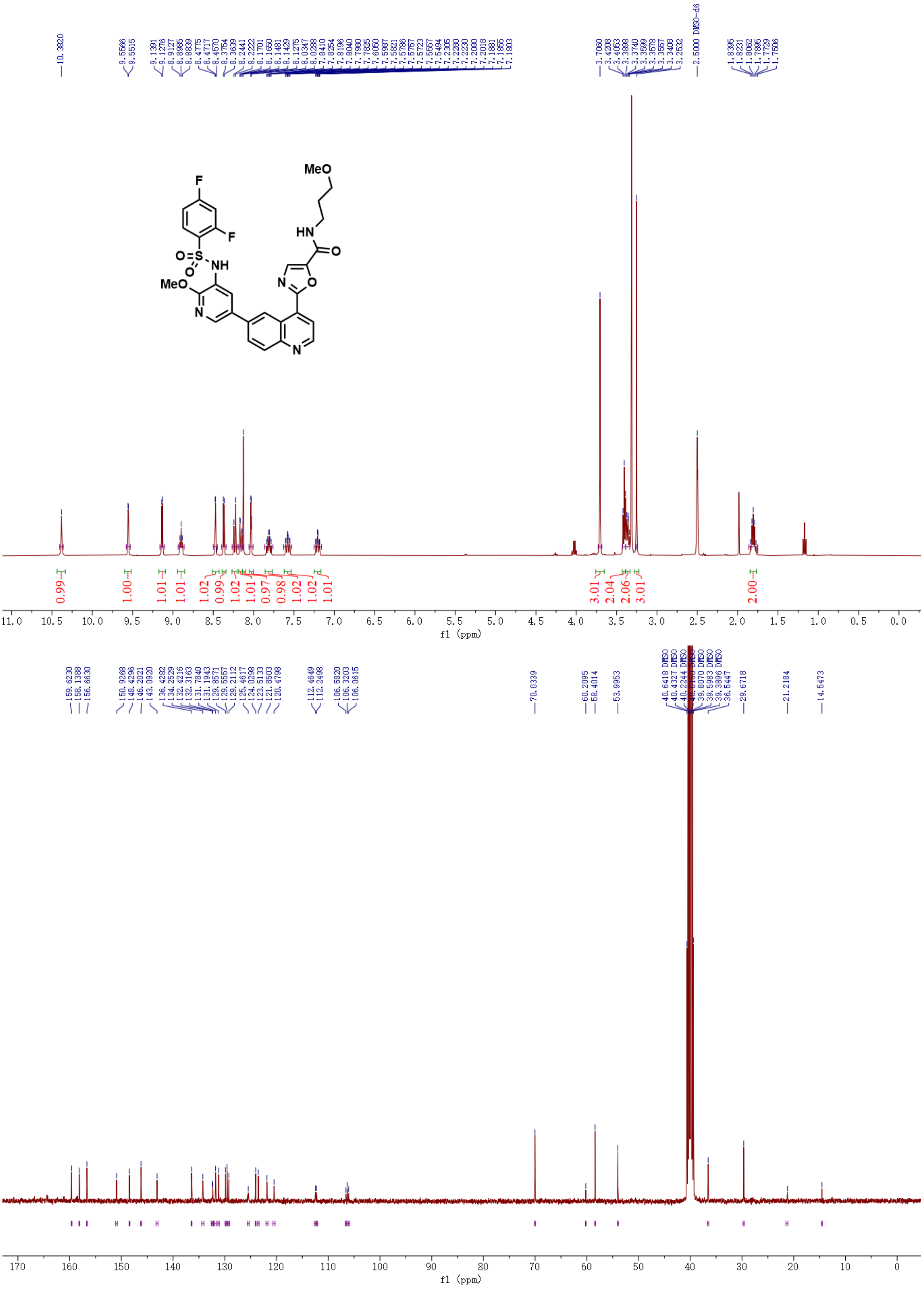

Figure S30. ^{1}H and ^{13}C NMR spectra of **22f** (DMSO-d6).

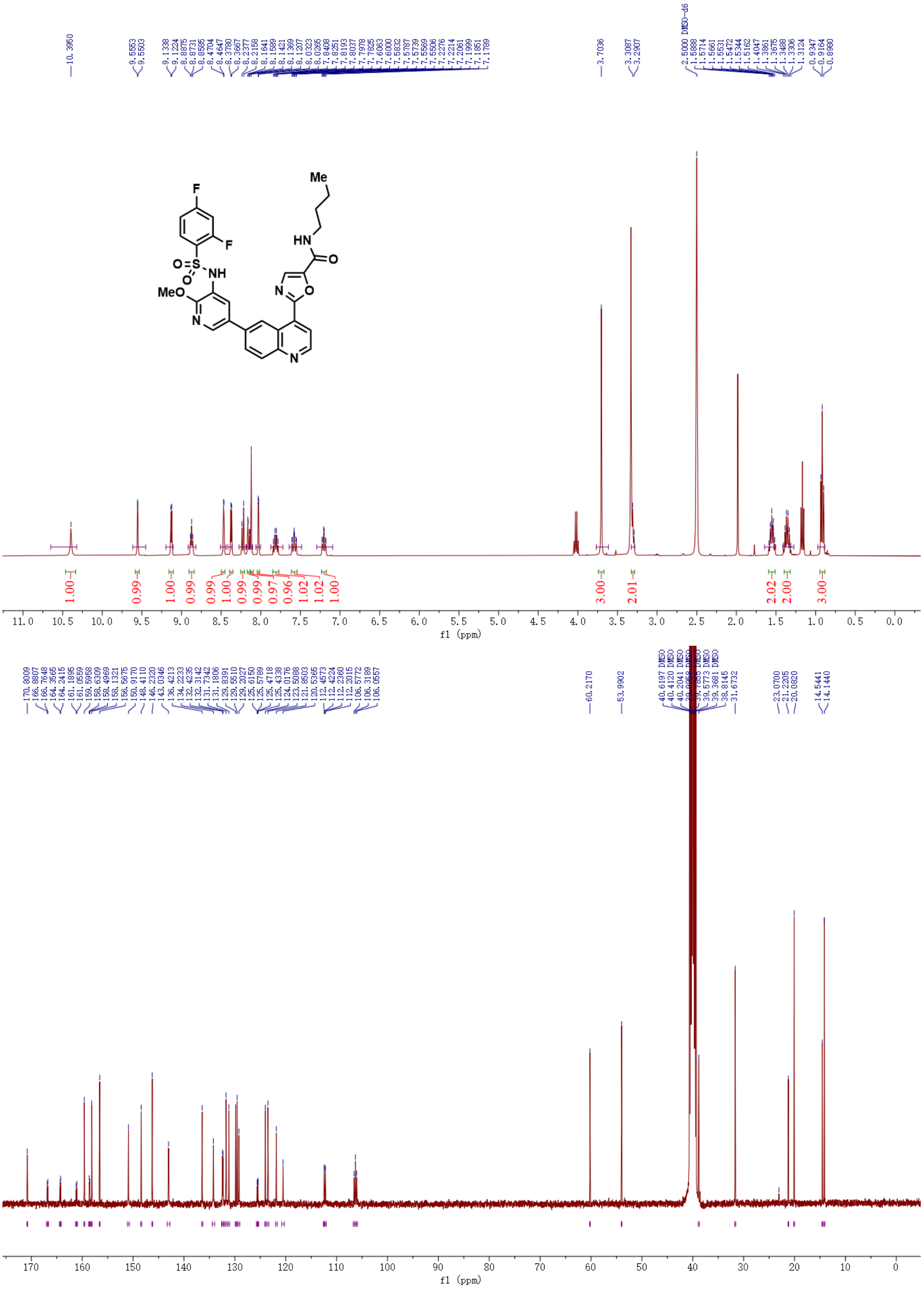

Figure S31. ^{1}H and ^{13}C NMR spectra of **22g** (DMSO-d6).

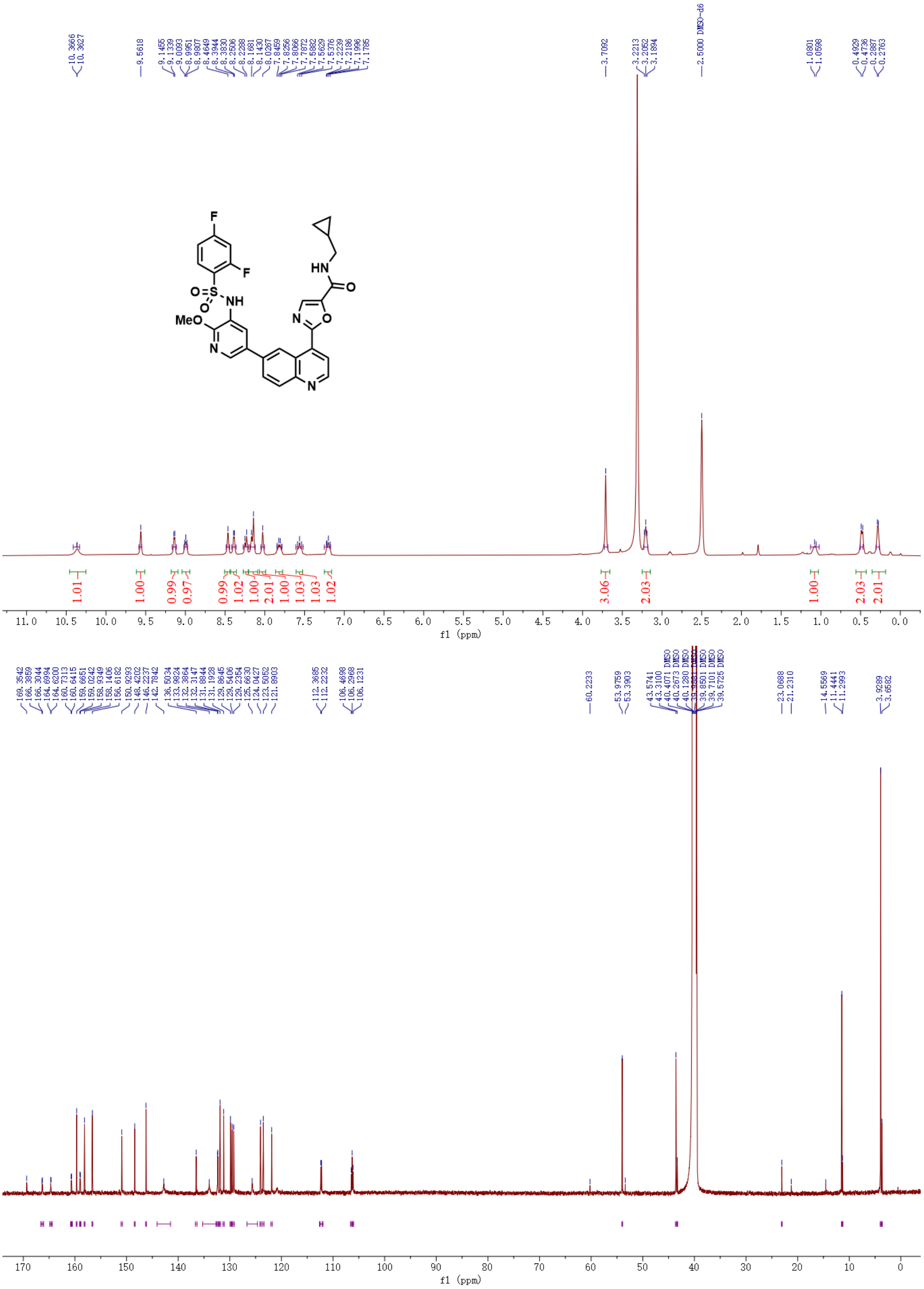

Figure S32. ^{1}H and ^{13}C NMR spectra of **22h** (DMSO-d6).

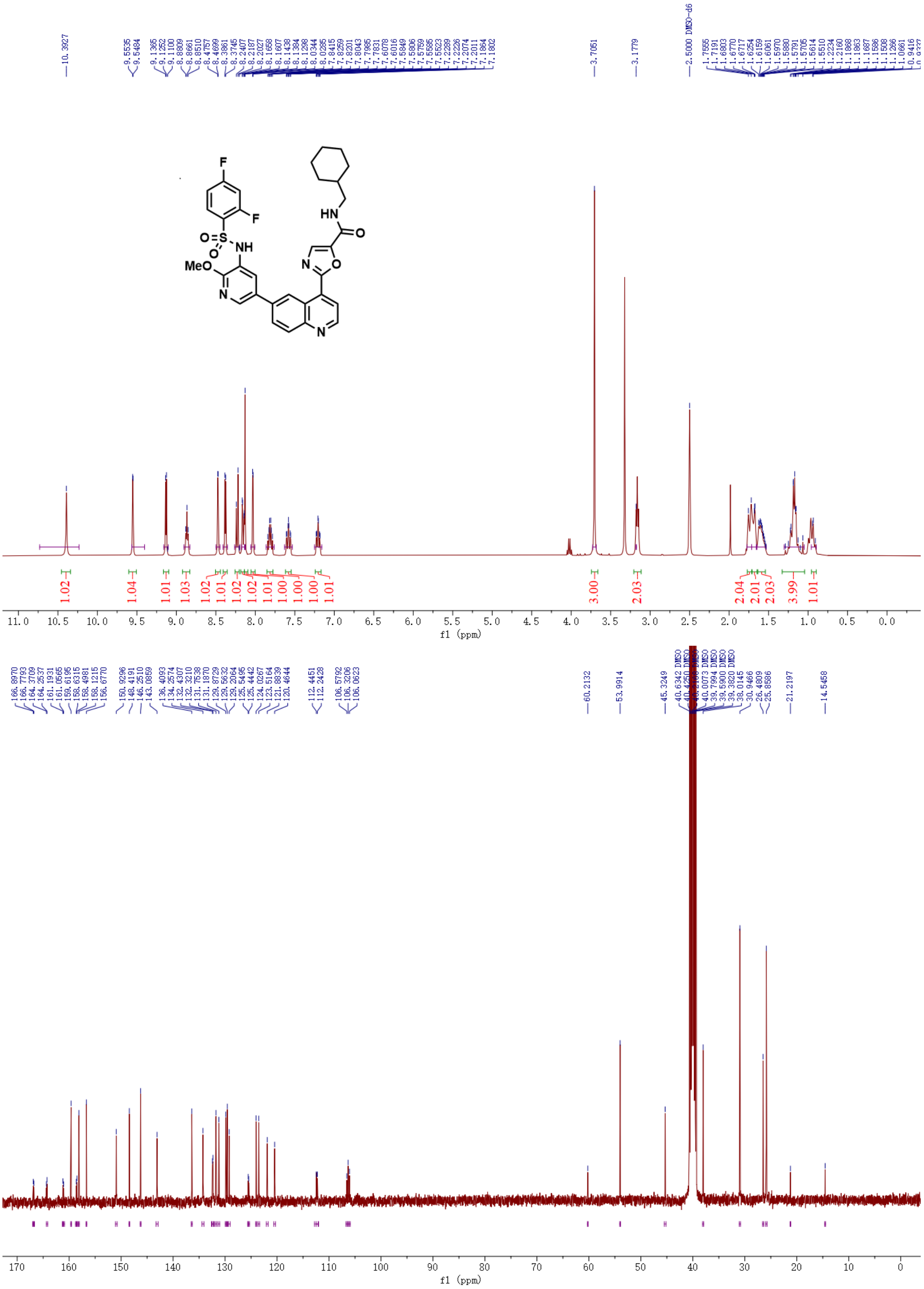

Figure S33. ^{1}H and ^{13}C NMR spectra of **22i** (DMSO-d6).

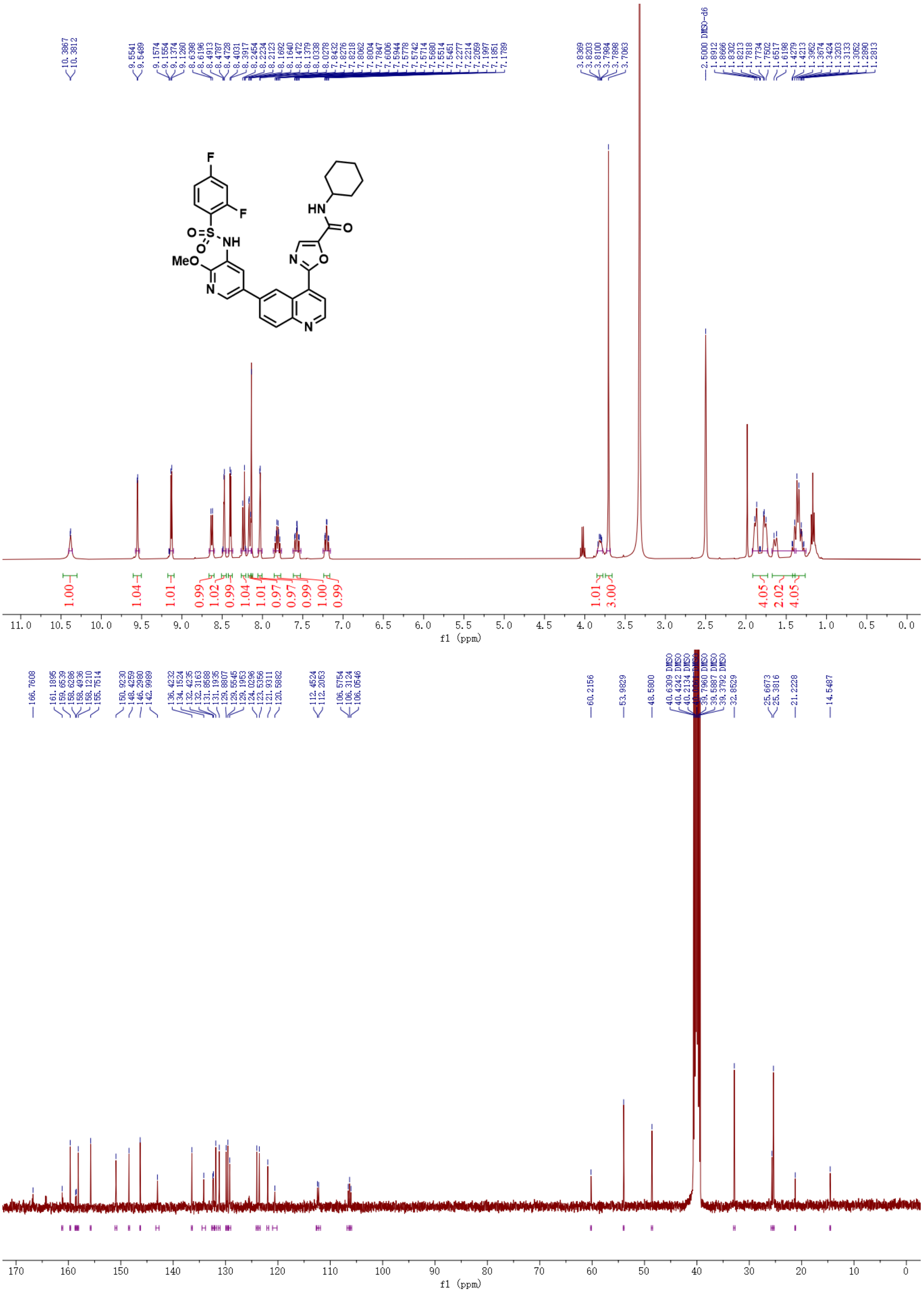

Figure S34. ^{1}H and ^{13}C NMR spectra of **22j** (DMSO-d6).

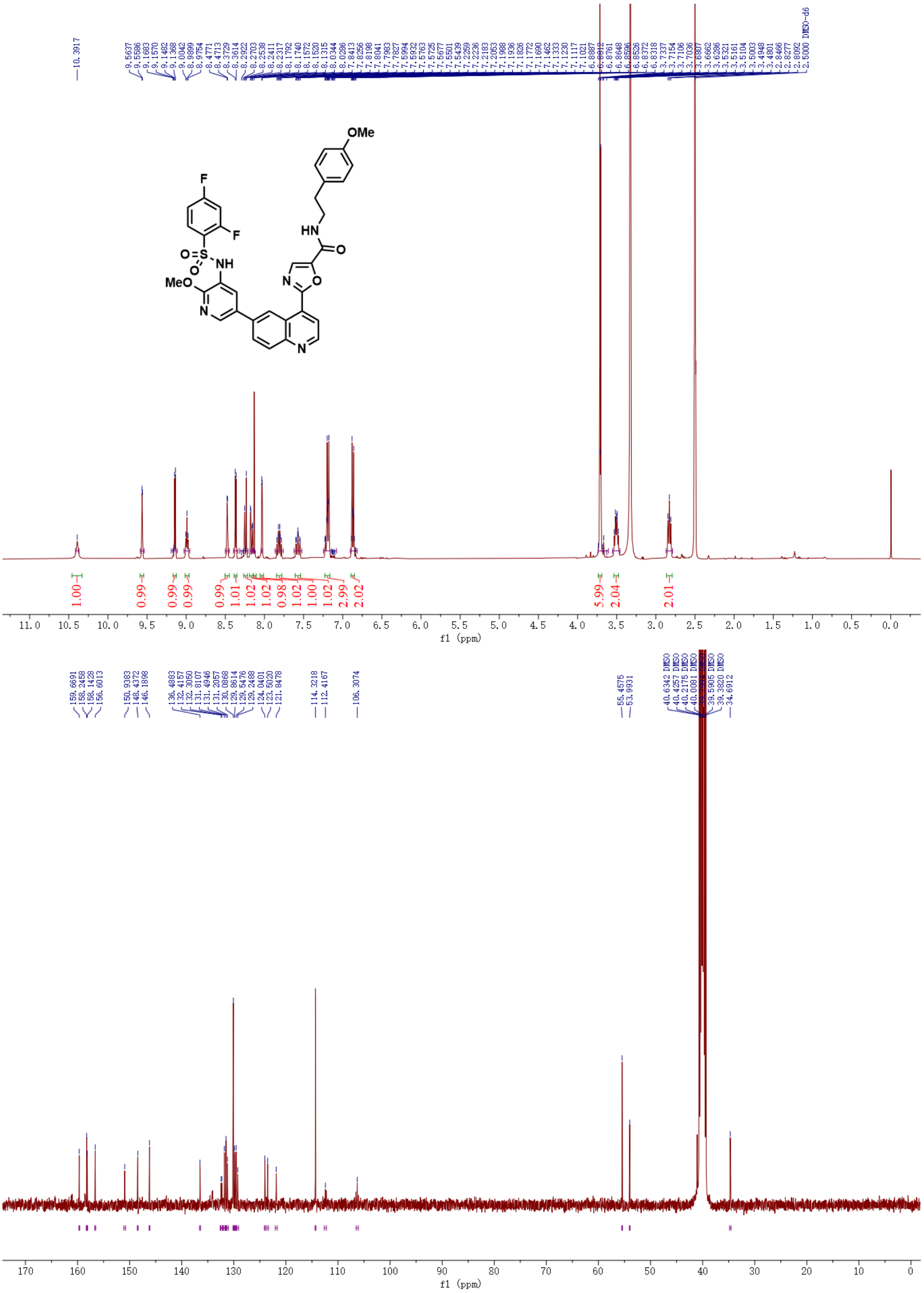

Figure S35. ^{1}H and ^{13}C NMR spectra of **22k** (DMSO-d6).

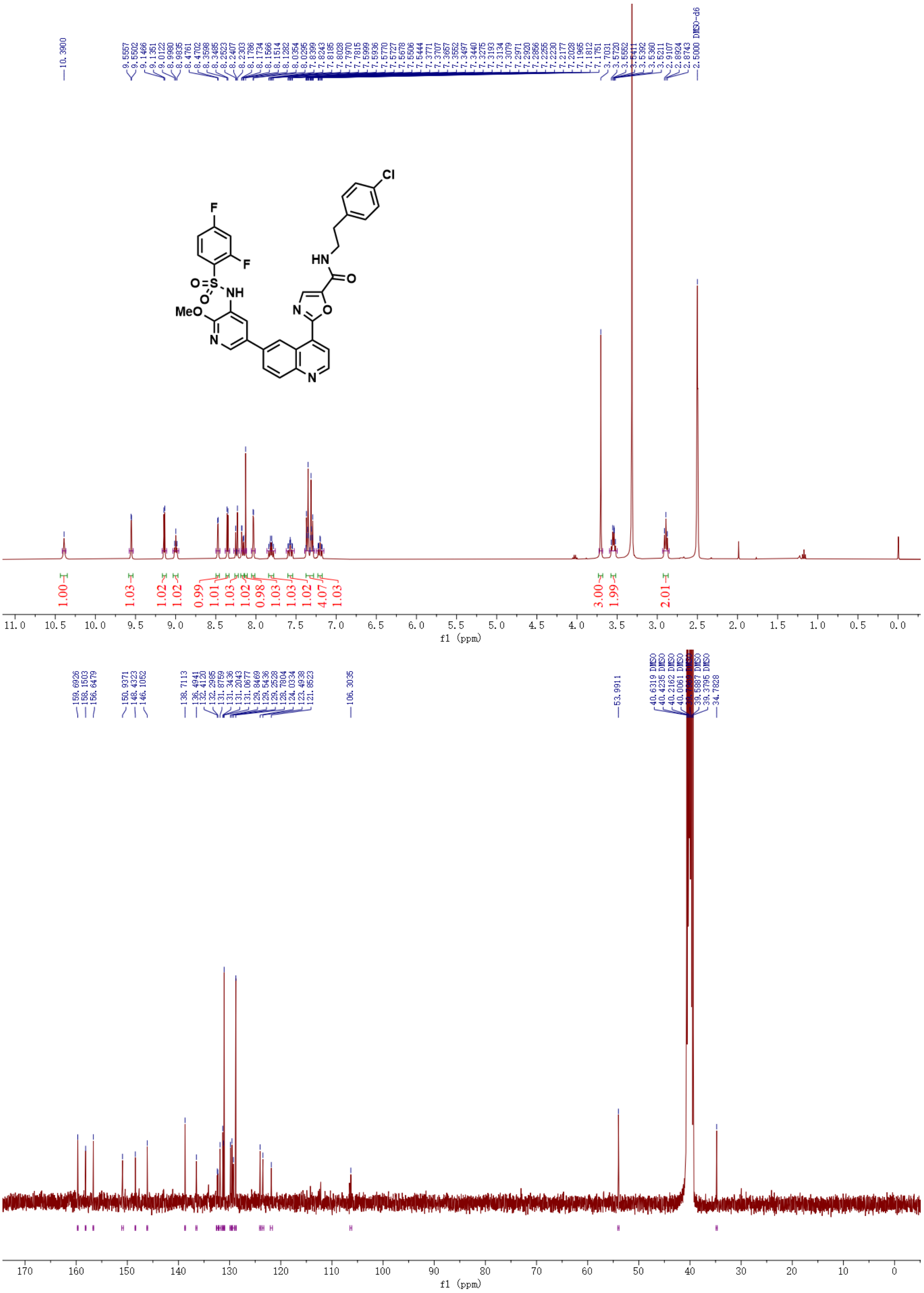

Figure S36. ^{1}H and ^{13}C NMR spectra of **22l** (DMSO-d6).
